# Drosophila species learn dialects through communal living

**DOI:** 10.1371/journal.pgen.1007430

**Published:** 2018-07-19

**Authors:** Balint Z. Kacsoh, Julianna Bozler, Giovanni Bosco

**Affiliations:** Department of Molecular and Systems Biology, Geisel School of Medicine at Dartmouth, Hanover, New Hampshire, United States of America; University of Arizona, UNITED STATES

## Abstract

Many species are able to share information about their environment by communicating through auditory, visual, and olfactory cues. In *Drosophila melanogaster*, exposure to parasitoid wasps leads to a decline in egg laying, and exposed females communicate this threat to naïve flies, which also depress egg laying. We find that species across the genus Drosophila respond to wasps by egg laying reduction, activate cleaved caspase in oocytes, and communicate the presence of wasps to naïve individuals. Communication within a species and between closely related species is efficient, while more distantly related species exhibit partial communication. Remarkably, partial communication between some species is enhanced after a cohabitation period that requires exchange of visual and olfactory signals. This interspecies “dialect learning” requires neuronal cAMP signaling in the mushroom body, suggesting neuronal plasticity facilitates dialect learning and memory. These observations establish Drosophila as genetic models for interspecies social communication and evolution of dialects.

## Author summary

In this study, we find that many different Drosophila species never having been exposed to parasitoid wasps can trigger caspase activation in the ovary and depress egg-laying when placed next to flies that had visual experience with wasps. Interestingly, when teacher flies of one species are placed with a student of a different species, communication exists, to varying degrees, which seems dependent on evolutionary relatedness. Cohabitation of two species that can partially communicate can learn each other’s “dialect”, yielding effective interspecies communication. There are various inputs involved in dialect learning, including the presence of visual and olfactory cues and memory functions, including genes implicated in social learning defects in murine models, such as PTEN. The neuroplasticity of adult Drosophila allows for learning of dialects, but the specific dialect learned is dependent on social interactions exclusive to a communal environmental context, which provides both visual and olfactory inputs. We find flies can communicate with one another about an anticipated danger, which is suggestive of a fly “language.” The presence of a neurologically plastic system, allowing for social learning, can subsequently lead to a dramatic physiological response, requiring active learning and memory formation through integration of multiple inputs.

## Introduction

The ability to interpret environmental information is a phenomenon found throughout all life forms. From bacteria to plants and to mammals, communication occurs within as well as between species. In some cases, information that is being shared can be highly specific, such as in the case of honeybees communicating instructions on where to find nectar[1–3]. In other cases, opportunistic bystanders can also benefit from general information. For example, predator alarm calls generated as a warning are observed, where multiple species participate in repeating the alarm throughout the community[4–8]. In all cases, the information that is shared can be dependent on local environmental cues and experiences and the manner in which information is communicated is strongly influenced by past experiences of each individual. For example, birds, which live in geographically distinct populations, manifest unique song variants or regional dialects that can last for decades, but these animals are nevertheless still able to communicate with others of their species[9–11]. Because dialects are learned and therefore influenced[12] by specific local environmental differences, it suggests that both social and non-social experiences can have dramatic effects on cognitive development[13].

It is proposed that a myriad of environmental cues, both social and non-social, are critical to animal development in determining the ability to convey and receive specific types of information. However, there are many outstanding questions as a result of this proposition: What cues are important? When are these cues important? How can environmental cues interact with genetically determined developmental programs? Although social communication is most extensively documented in more derived species such as mammals and birds, insects can also display a broad range of behavioral tasks. Bees are known to be able to learn from non-natural sources in order to obtain a reward through social learning. Such information can be passed on to naïve, student bees through the use of visual cues[14,15]. Insect social learning extends to the genetic model system of Drosophila, where student, observer flies learn from a trained, teacher-fly, using visual cues. This has been shown in communication involving food sources and predator threats[16,17].

Chemical cues can serve as intra- and interspecies signals, such as fox and guinea pig urine affecting not only conspecific behavior, but also the behavior of other animals[18–20]. Sound can also be used, such as in bats and bottlenose dolphins, which are able to distinguish members of the community through the use of echolocation pitch recognition[21,22]. Plants have a vast arsenal of responses to pathogens[23], including communicating a threat to neighboring plants through the use of volatile organic compounds[24]. Plant interspecies[25–31] and intraspecies[32–34] communication occurs both in laboratory settings and in the wild[30,35].

*Drosophila melanogaster* and other Drosophila species have provided insights into mechanisms of learning, memory, and complex behaviors[36,37]. However, these behaviors and phenotypes have been studied almost exclusively in domesticated *D*. *melanogaster* lab monocultures, while *D*. *melanogaster* wild populations are surrounded by a broad range of predators, microbes, and other Drosophilids, highlighting a communal component of the organism’s life cycle[38]. This raises the possibility of behavioral phenomenon that have yet to be discovered and analyzed in domesticated lab monocultures[39–41]. Given the vast range of environmental inputs on a wild Drosophilid, a fly must be able to discern important information from extraneous inputs, while interacting with conspecifics and a variety of other species [42–46].

Although modes of intra- and interspecies communication are likely to be genetically limited, there is also value in learning to interpret signals from variable, local environments that may provide immediate survival benefits. How do genetically constrained neurological features and variable environmental factors interact to produce context-dependent, meaningful information? Under which environmental factors would information sharing between different species occur and be beneficial? In this study, we sought to begin to address these questions in the Drosophila model system by using a pan-Drosophila predator known to elicit social communication [17,47]. *D*. *melanogaster* presented with parasitoid wasps have multiple behavioral responses, including a reduction in oviposition (egg laying) through an increase in ovarian apoptosis [17,48–51]. After removal of the wasp, a wasp-exposed “teacher” fly can instruct a naïve “student” fly about the presence of the wasp threat through the exclusive use of visual cues, such that students now reduce their own oviposition by triggering ovarian apoptosis. Using this fly-fly social communication paradigm we asked (1) whether social communication is conserved among other Drosophila species, (2) if Drosophilids engage in interspecies communication, and (3) what environmental and genetic factors are required for interspecies communication.

## Results

### Intra- and interspecies communication

**Fig 1:**
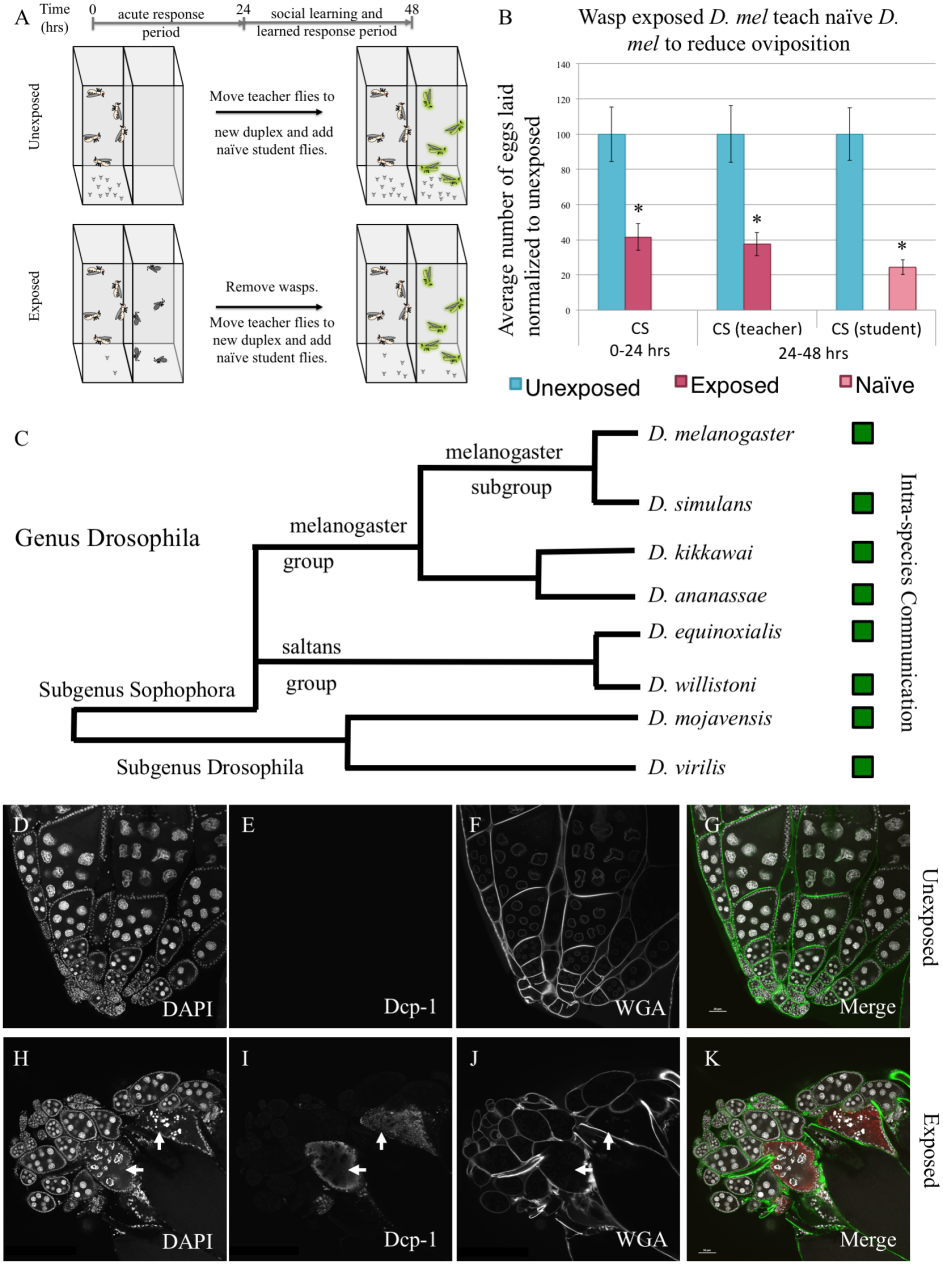
A predator threat is communicated through visual cues within species across the genus Drosophila, modulating reproductive behavior and caspase activation. (A) Standard experimental design. (B) Percentage of eggs laid by exposed flies normalized to eggs laid by unexposed flies is shown. Wild-type *D*. *melanogaster* (Canton S) exposed to wasps lay fewer eggs than unexposed flies. (C) Phylogeny of 8 species tested across the genus Drosophila that demonstrate the ability to communicate through visual cues. Green boxes indicate social learning is present in species tested. Representative ovary of control and wasp exposed Drosophila showing caspase activation (*D*. *melanogaster*). DAPI (D, H), activated Dcp-1 (E, I), WGA (F,J), and the merged images (G, K) are shown. Arrows denote apoptotic egg chambers. Error bars represent standard error (n = 12 biological replicates) (*p < 0.05).

We utilized the fly duplex, an apparatus with two transparent acrylic compartments to test whether different species respond to seeing predators (acute response) and if exposed “teacher” female flies can communicate this threat to naïve unexposed “student” female flies[17]. The duplex allows flies to see other flies or wasps in the adjacent compartment, without direct contact, making all communication only visual (Fig 1A). Ten female and two male flies are placed into one duplex compartment, with an adjacent compartment containing twenty female wasps. Following a 24-hour exposure, wasps are removed and acute response is measured by counting the number of eggs laid in the first 24-hour period in a blinded manner. Flies are shifted to a new duplex, with ten female and two male naïve student flies in the adjacent compartment (Fig 1A, see Methods). Following a second 24-hour period, all flies are removed and the response of both teacher and student is measured by counting the number of eggs laid in a blinded manner. The 24-48-hour period measures memory of teachers having seen the wasps and students having learned from the teachers. Using wild-type *D*. *melanogaster*, we find both an acute response and a memory response to the wasp in teacher flies and a learned response in naïve student flies (Fig 1B, S1A Fig, S1 File for all raw egg counts and p-values in this study) [17,50,51].

We then asked whether the acute, memory, and student social learning behaviors are conserved in other Drosophila species, with varying relatedness to *D*. *melanogaster* ranging from sister species, such as *D*. *simulans*, to very distantly related species, such as *D*. *virilis*. For each species, we tested a sister species as an additional way to validate our observations. Across a broad span of the genus Drosophila, we find the conservation of both the acute and memory responses in teacher flies in addition to the ability of teachers to communicate to conspecific student flies. (Fig 1C, S1B–S1H Fig). Some of these species have been previously shown to depress oviposition during wasp exposure [51]. Our experimental design allows for only visual cues to be detected from the wasps and from teachers to student flies. Thus, in all species tested, visual cues are sufficient for flies to detect wasps and for naïve flies to learn from wasp-exposed teacher flies. Conservation of these behaviors is especially impressive as the species tested are separated by millions of years of evolution, yet the basic behaviors observed in *D*. *melanogaster* are maintained. Moreover, this conservation further underscores the importance this innate behavior must have since even laboratory cultures that have not experienced wasp for many generations nevertheless exhibit a robust response. In particular, the conservation of the fly-fly communication behavior speaks to a presence of a conserved form of fly signaling and signal interpretation, which we suggest might be thought of as a “fly language” in this paradigm.

Oviposition reduction is modulated in part by the effector caspase Dcp-1[17]. In *D*. *melanogaster*, we observe overlapping staining of activated Dcp-1 with a punctate pattern of DNA staining with 4’, 6-diamidino-2-phenylindole (DAPI), indicative of oocyte specific apoptotic activity (Fig 1D–1K, S2 Fig). We performed immunofluorescence with antibodies specific to activated Dcp-1 across a broad range of Drosophila species, revealing cleaved caspase following wasp exposure in all 15 Drosophila species tested (S3–S16 Figs). We observed an increase in positive cleaved caspase oocytes following wasp exposure (S17 Fig), along with a decrease in total number of egg chambers (S18 Fig), suggestive of ovarian apoptosis and elimination of oocytes[17]. Phylogenetic trees shown are adapted from previous work [52].

**Fig 2:**
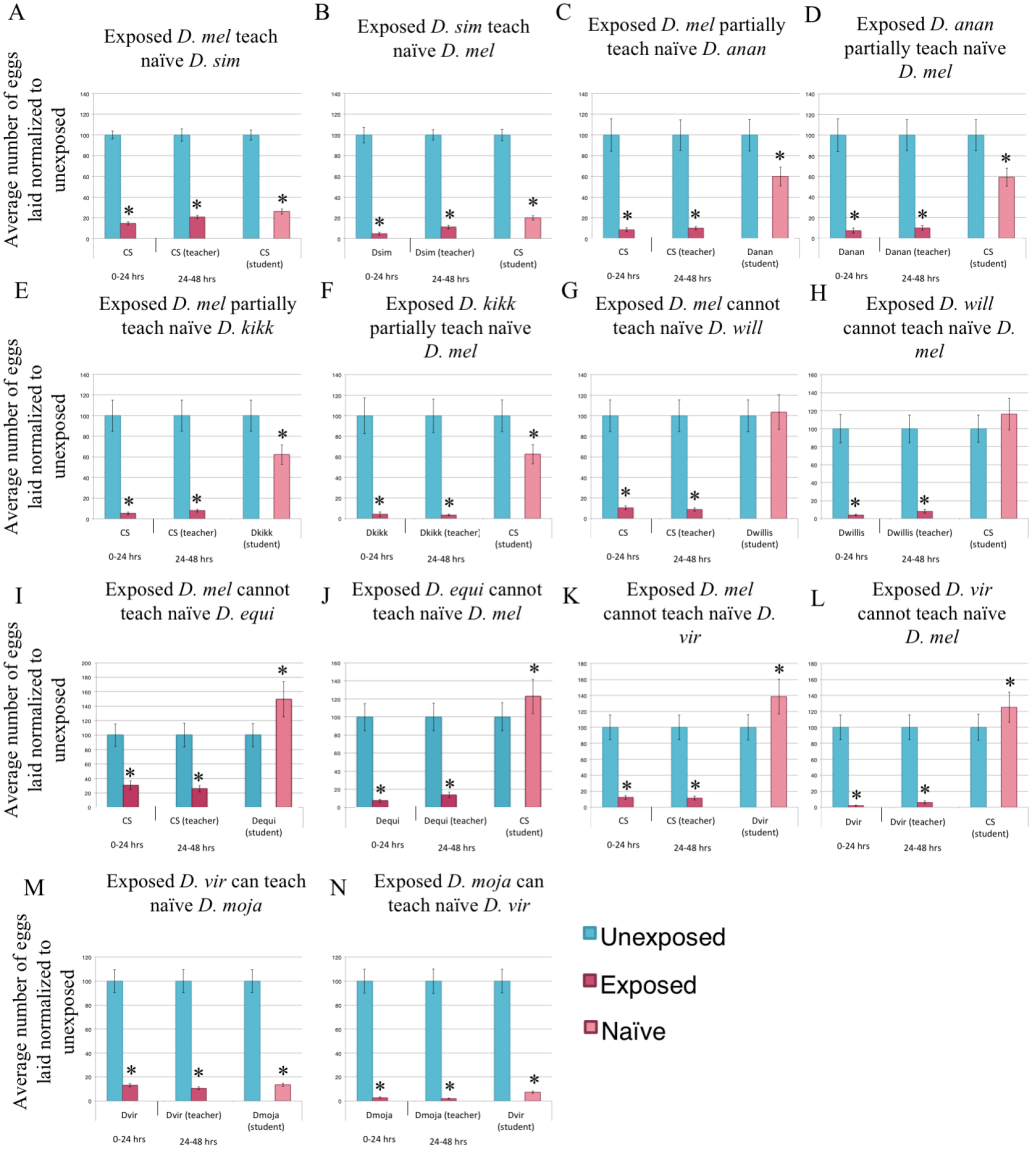
Interspecies communication of predator threats. Percentage of eggs laid by exposed flies normalized to eggs laid by unexposed flies is shown. Flies exposed to wasps lay fewer eggs than unexposed flies. Communication between *D*. *melanogaster* and: *D*. *simulans* (A, B), *D*. *ananassae* (C, D), *D*. *kikkawai* (E,F), *D*. *willistoni* (G,H), *D*. *equinoxialis* (I,J) and *D*. *virilis* (K,L) shows varying communication abilities. Communication between *D*. *virilis* and *D*. *mojavensis* occurs (M,N). Error bars represent standard error (n = 12 biological replicates) (*p < 0.05).

Following the observation that an acute response to wasps and intraspecies communication is conserved across the genus, we asked whether the wasp threat could be communicated between two different species. We utilized 15 Drosophila species that respond to wasps to answer this question (S3–S18 Figs). The species were selected to span the phylogeny with different degrees of relatedness to *D*. *melanogaster* [52]. We find that *D*. *melanogaster* are able to communicate the threat to and receive communications from closely related species, such as *D*. *simulans* and *D*. *yakuba*, with oviposition of students paired with wasp-exposed teachers being ~10–30% compared to unexposed (Fig 2A and 2B, S19A–S19F Fig). Interestingly, species more distantly related to *D*. *melanogaster*, such as *D*. *ananassae* and its sister species, elicit a partial communication phenotype, with oviposition depression of students paired with wasp-exposed teachers being ~50–65% of unexposed flies (Fig 2C–2F, S19G–S19J Fig). A second strain isolate of *D*. *ananassae* also show partial communication with *D*. *melanogaster* (Fig 2C–2F, S19G–S19J Fig). Species more distantly related to *D*. *melanogaster*, such as *D*. *willistoni*, *D*. *equinoxialis*, and *D*. *virilis*, cannot communicate with *D*. *melanogaster* (Fig 2G–2L, S19K–S19P Fig). We statistically characterized these category assignments based on the criteria of mean value and statistical significance compared to unexposed in order to define efficient, partially, and lack of communication (S2 File, Methods). Collectively, the data suggest that evolutionary distance contributes to the efficiency of interspecies communication. *D*. *ananassae* show varying communication phenotypes with other Drosophila species, though the pattern of communication is different. For example, *D*. *ananassae* exhibit partial communication with *D*. *simulans* (S20A and S20B Fig), strong communication with its sister *D*. *kikkawai* (S20C and S20D Fig), and partial communication with *D*. *equinoxialis* and *D*. *willistoni* (S20E–S20H Fig). *D*. *ananassae*, in addition to *D*. *melanogaster*, are unable to communicate with the distantly related *D*. *mojavensis* and *D*. *virilis* (Fig 2I and 2J, S20I–S20L Fig). Species such as *D*. *virilis*, which were unable to communicate with *D*. *melanogaster* and *D*. *ananassae*, can communicate with other species, such as its sister species *D*. *mojavensis* (Fig 2K and 2L). Thus, although all species tested are capable of intraspecies communication, there is a fundamental, species-specific difference in communication mode or “fly language” when communicating wasp predator threat.

### Fixed versus plastic behavior

We wished to assay whether the observed communication behavior is hardwired in the fly brain, or if it had a level of plasticity as a result of socialization. Known learning and memory mutants have shown defects in socialization assays [53,54]. Additionally, the cuticular hydrocarbon composition on flies changes as a function of social, but not sexual, experience [55], though sexual experience is also affected by isolation [56]. Thus, we sought to assay whether socialization, which has been shown to have behavioral affects in other assays [57] including egg laying behavior [44,58], has an effect on intraspecies communication.

**Fig 3:**
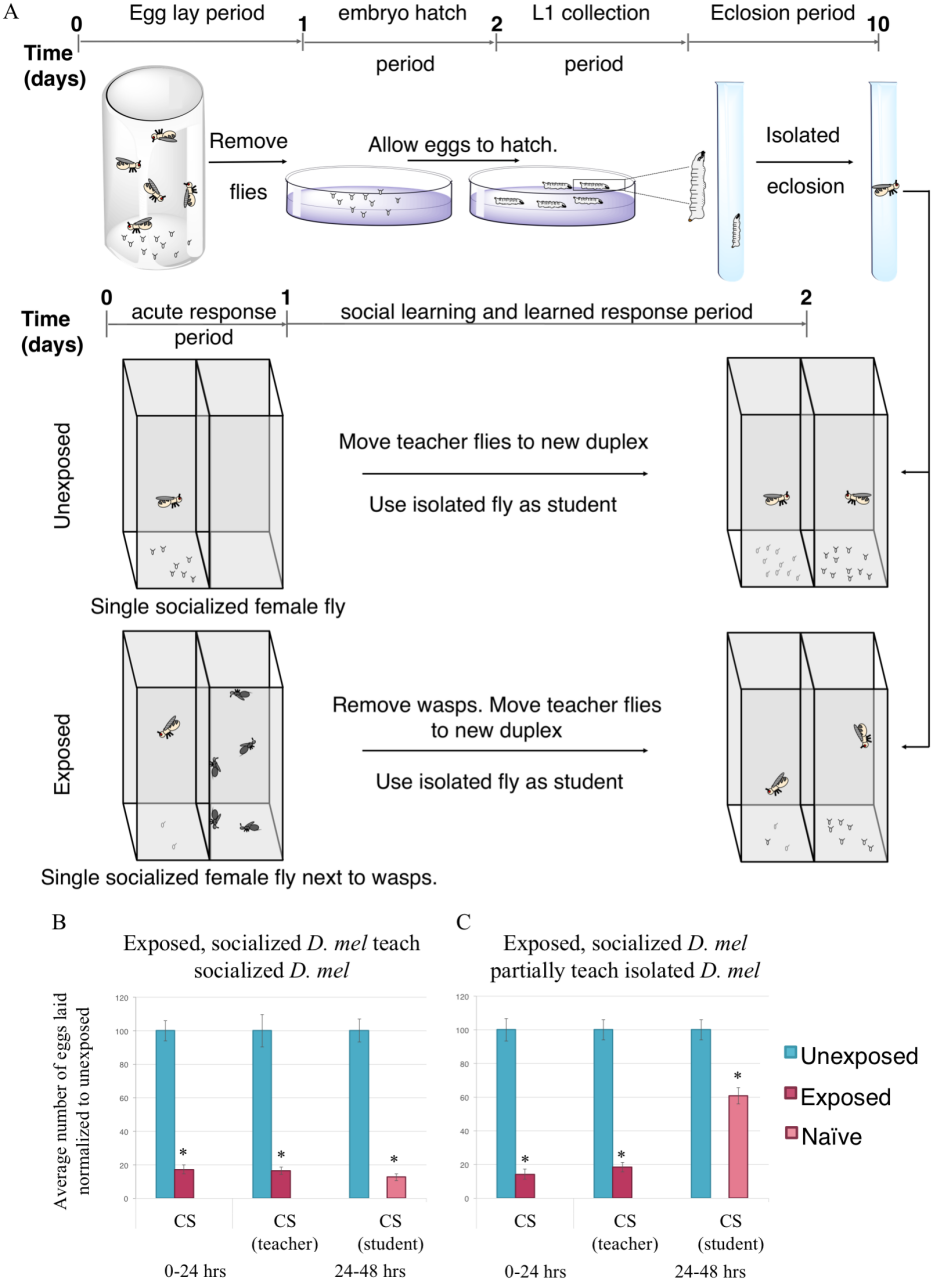
Flies raised in isolation have difficulty learning from socialized teachers. (A) Experimental design of isolation of L1 larvae. Flies are allowed to lay for 24 hours, after which the adults are removed. Eggs are allowed to hatch and L1 larvae are isolated in an individual tube and allowed to eclose. A single isolated female fly is paired with a single isolated male and used as students to a single male and single female fly teacher that are socialized throughout life. Percentage of eggs laid by exposed flies normalized to eggs laid by unexposed flies is shown. Communication between socialized *D*. *melanogaster* teachers and: *D*. *melanogaster*, socialized students showing strong communication (B) and *D*. *melanogaster* students raised in isolation showing partial communication ability (C). Error bars represent standard error (n = 24 biological replicates) (*p < 0.05).

To ask this question, we collected L1 larvae and isolated each larva in a Falcon round-bottom polypropylene tube containing 1 mL standard Drosophila media. Larvae were allowed to pupate and eclose in isolation. Each tube was kept separate such that no visual information could be transferred between individuals in tubes. Following eclosion, 1 female aged 3–5 days old was used as the student, paired with one socialized female teacher (Fig 3A). This 1:1 ratio was first tested with *D*. *melanogaster* where both teachers and students were previously socialized, observing typical strong communication (Fig 3B). Interestingly, the flies raised in isolation presented with a partial communication phenotype, similar to when normally socialized *D*. *melanogaster* and *D*. *ananassae* are paired (Fig 3C). Larval isolation has been previously shown to have effects on cooperative larval behavior, and thus, we cannot rule out the possibility that isolation of larvae translates to behavioral defects in adult flies [59]. However, given the observation of this partial communication phenotype, we suggest that while the ability to communicate is hardwired in the fly brain, there exists a degree of plasticity that is dependent on previous socialization.

### Dialect learning

**Fig 4:**
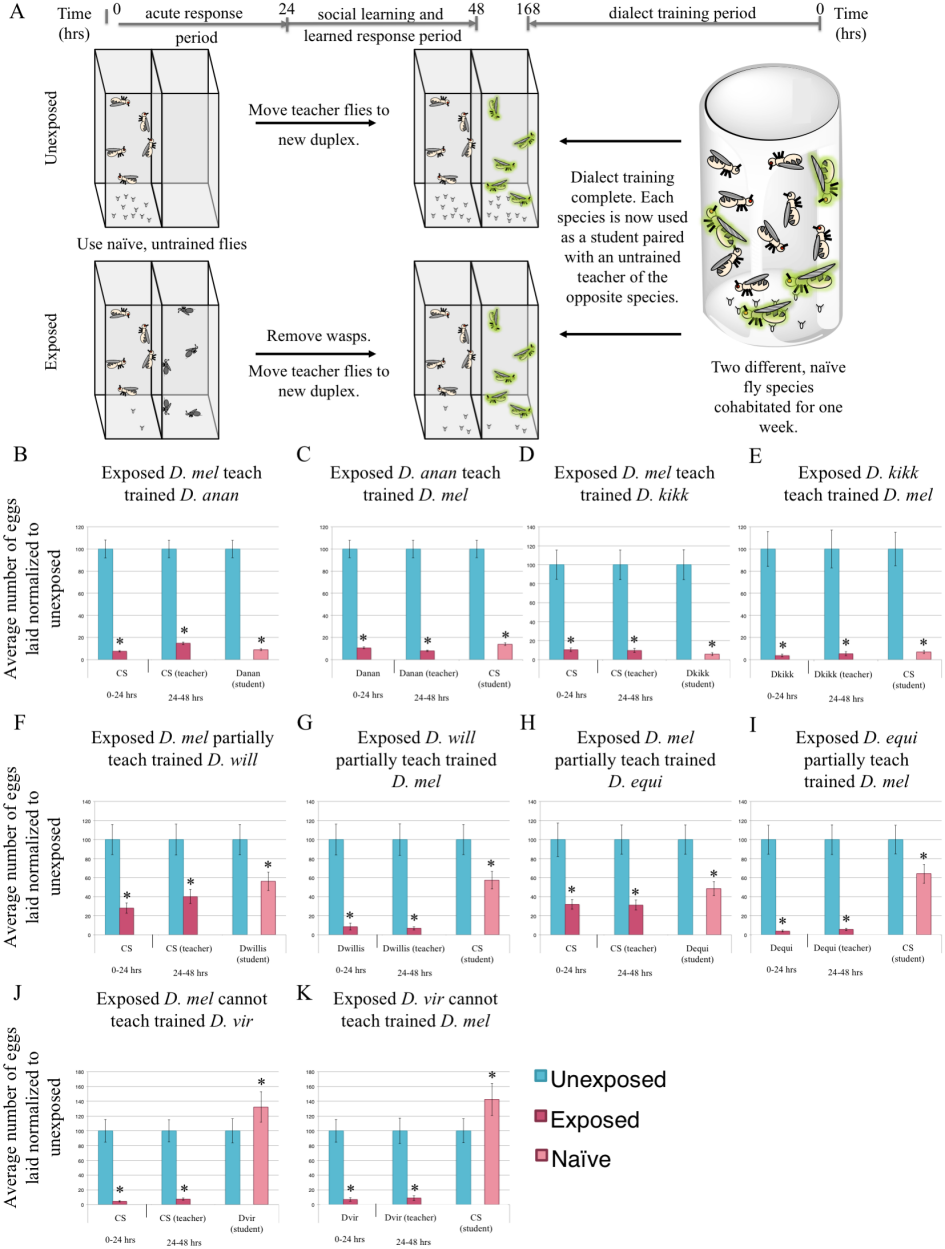
Species cohabitation enables inter-species communication. (A) Experimental design of dialect training for flies that are used as students. Two species are cohabitated for one week prior to being used as students for naive, untrained teacher flies of the opposite species. Percentage of eggs laid by exposed flies normalized to eggs laid by unexposed flies is shown. Communication between trained students *D*. *melanogaster* and: *D*. *ananassae* showing strong communication following cohabitation (B, C), *D*. *kikkawai* showing strong communication following co-incubation (D, E), *D*. *willistoni* showing partial communication following co-incubation (F,G), *D*. *equinoxialis* showing partial communication following co-incubation (H,I), and *D*. *virilis* showing no communication following co-incubation (J,K). Error bars represent standard error (n = 12 biological replicates except for (C), n = 24 replicates) (*p < 0.05).

Given that our isolation experiments demonstrate a level of plasticity dependent on socialization, we wished to explore the possibility that partial communication between species might be alleviated as a result of socialization between two different species. Since closely related species can communicate the threat of a wasp, we postulated that environmental factors contributing to interspecies communication for distantly related species may be partially dependent on socialization. To test this idea, *D*. *melanogaster* were cohabitated with species capable of only partial communication (*e*.*g*. *D*. *ananassae*) (Fig 2C and 2D). Cohabitation lasted for one week in a single container, allowing for frequent and multiple channels of sensory interactions. Following a weeklong cohabitation period, the two species were separated and used as students paired with teachers of the other species (Fig 4A). In all experiments teachers had existed only as a monoculture, kept separate from other species monocultures, while all flies experiencing an interspecies cohabitation period were subsequently used only as students.

If cohabitation of different species results in exchange of information that later facilitates communication between these two species, then we predict that species capable of only partial communication may then be capable of full or enhanced communication. We find that cohabitation can greatly enhance communication between some species, suggesting that some form of training occurs during this period. After cohabitation, *D*. *ananassae* students learn very efficiently from *D*. *melanogaster* teachers, demonstrating that cohabitation of two species yields an expanded communication repertoire (Fig 4, S21 Fig). This observation indicates that poorly communicating species are not limited by structural barriers such as wing shape or olfactory capacity. Instead this suggests that, similar to local dialects in bird songs, Drosophila species-specific cues can be learned simply by repeated exposure to the “dialect”, and provides further evidence for the role of socialization in Drosophila communication (Fig 3). Thus, there exists a variation of signal among populations of different species of Drosophila, even though there exists a conserved fly “language” to communicate the threat of a wasp. Thus, we suggest this to be analogous to “dialects” as it reflects natural variations between a common mode of communication, which can be alleviated through socialization between species. Hereafter we refer to this cohabitation as a “dialect learning” period.

We observed dialect learning in two different *D*. *ananassae* strain isolates, and two additional sister species (Fig 4B–4E, S21A–S21G Fig), indicating that dialect learning is likely to be a wide-spread phenomenon in Drosophila. Interestingly, some distantly related species that were unable to communicate with *D*. *melanogaster* (i.e. *D*. *willistoni*, *D*. *equinoxialis*) acquired the ability to partially communicate following a cohabitation-training period (Fig 4F–4I, S21H and S21I Fig). This was not the case for very distantly related species (i.e. *D*. *virilis*, *D*. *mojavensis*), which showed no ability to communicate with *D*. *melanogaster* even after a week-long cohabitation (Fig 4J and 4K, S21J–S21M Fig). We also tested a transgenic *D*. *melanogaster*, to see if it was capable of teaching and dialect learning, and find such flies can teach their dialect to and learn the dialect from *D*. *ananassae* (S21N and S21O Fig).

Additionally, we tested whether *D*. *ananassae* communication could benefit from cohabitation-training with species other than *D*. *melanogaster*. We find efficient communication between *D*. *simulans* (S22A and S22B Fig), *D*. *equinoxialis* (S22C and S22D Fig), and *D*. *mojavensis* (S22E and S22F Fig) with *D*. *ananassae* following a cohabitation-training period. In contrast to the *D*. *melanogaster* results, we find communication with more distantly related species is altered after dialect training. With *D*. *virilis* and *D*. *mojavensis*, in the untrained states, we observe no ability to communicate (S20I–S20L Fig), but find a partial communication phenotype following cohabitation (S22G–S22J Fig). *D*. *virilis* and *D*. *mojavensis*, although capable of interspecies communication and dialect learning, cannot learn the *D*. *melanogaster* dialect, but can learn *D*. *ananassae* dialect. These results suggest that some interspecies communication barriers do exist while others can be overcome by a period of dialect training during cohabitation.

**Fig 5:**
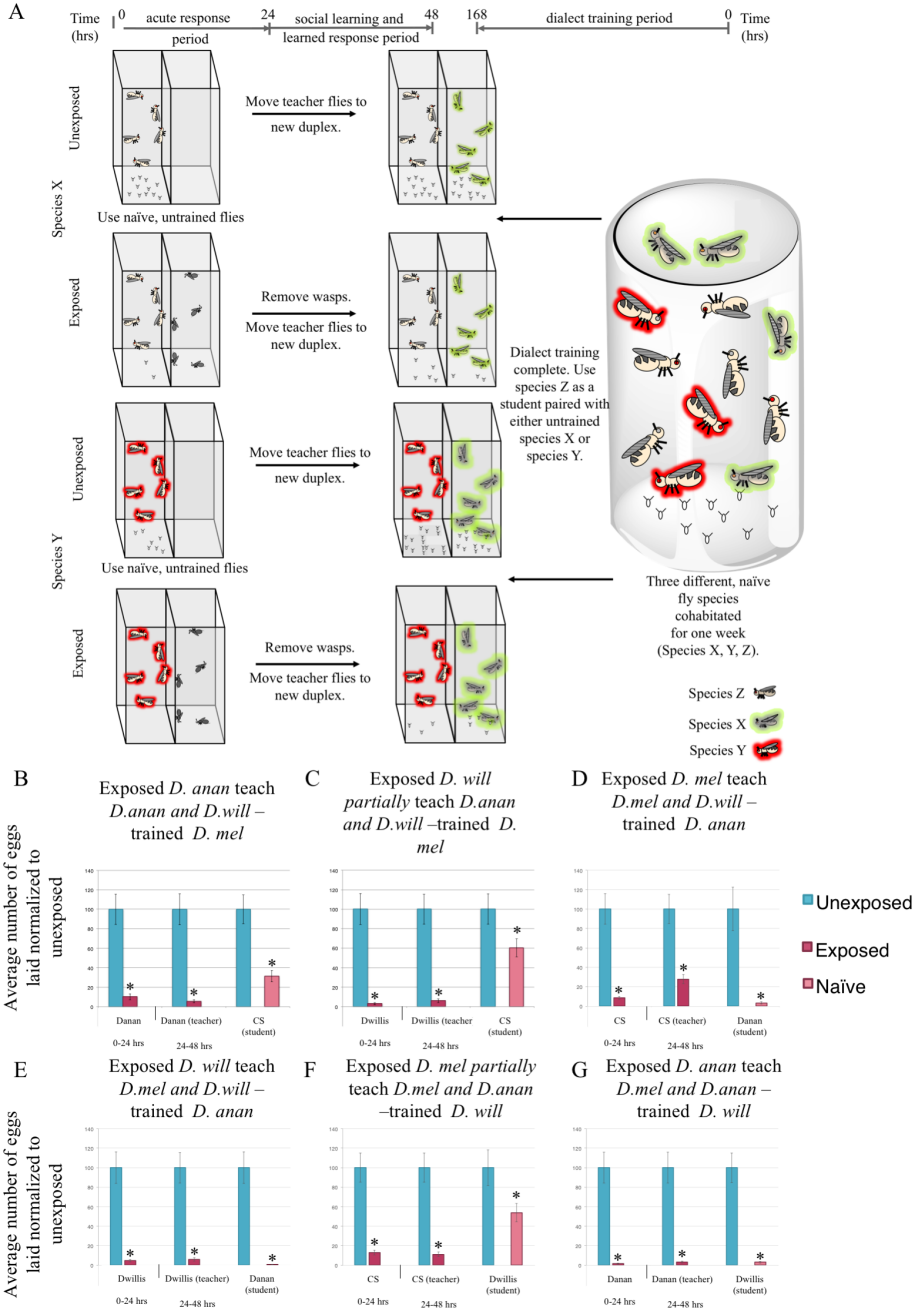
Flies can learn multiple dialects. (A) Experimental design of dialect training for flies that are used as students using multiple three unique species. Percentage of eggs laid by exposed flies normalized to eggs laid by unexposed flies is shown. Communication between *D*. *melanogaster* students trained by *D*. *ananassae* and *D*. *willistoni*, shows that *D*. *melanogaster* learn each species dialect even in the presence of more than one species (B, C). Communication between *D*. *ananassae* students trained by *D*. *melanogaster* and *D*. *willistoni*, shows that *D*. *ananassae* learn each species dialect even in the presence of more than one species (D, E). Communication between *D*. *willistoni* students trained by *D*. *melanogaster* and *D*. *ananassae*, shows that *D*. *willistoni* learn each species dialect even in the presence of more than one species (F, G). Error bars represent standard error (n = 12 biological replicates) (*p < 0.05).

Given our observation that two species can learn dialects following a cohabitation-training period, we wondered whether having more species present during the dialect training period influences dialect learning. In nature, flies encounter many different species of Drosophila, and given this knowledge, we hypothesized that neuronal plasticity exists in the fly brain to allow flies to learn multiple dialects from a given training period that includes multiple species as inputs. To probe this question, *D*. *melanogaster* were cohabitated with species capable of only partial communication or no communication in the untrained state, but show efficient and partial communication after dialect training (*i*.*e*. *D*. *ananassae* and *D*. *willistoni*, respectively). These three species were cohabitated for one week in a single container. We then used the trained *D*. *melanogaster* as students with untrained *D*. *ananassae* and *D*. *willistoni* teachers (Fig 5A). We find that trained *D*. *melanogaster* are able to efficiently communicate with *D*. *ananassae* and partially communicate with *D*. *willistoni* (Fig 5B and 5C). These results mirror assays where these species were individually trained (Fig 4B, 4C, 4F and 4G), suggesting that flies can simultaneously make use of multiple inputs from multiple species and be able to learn and remember each unique dialect they encounter. Additionally, we tested *D*. *ananassae* and *D*. *willistoni* as students that were cohabitated with *D*. *melanogaster*. We find that *D*. *ananassae* can communicate efficiently with *D*. *melanogaster* and *D*. *willistoni* (Fig 5D and 5E), and that *D*. *willistoni* can partially communicate with *D*. *melanogaster* and effectively communicate with *D*. *ananassae* (Fig 5F and 5G). These data also mirror individual training (Fig 4B, 4C, 4F and 4G, S22E and S22F Fig). Collectively, these data demonstrate that a fly can have vast communication repertoires consisting of multiple dialects that it acquires.

Given the result above with multiple species being able to learn multiple dialects, we wondered the level of specificity and the level of generalization of dialect learning as a means to provide insight into the identity of the “signal” being transferred between species. To test this, we performed cohabitation of *D*. *melanogaster* and *D*. *kikkawai*, a sister species to *D*. *ananassae*. We then assayed the communication ability of *D*. *melanogaster* with either *D*. *ananassae* or *D*. *willistoni* (S23A Fig). We find that *D*. *kikkawai* trained *D*. *melanogaster* are able to effectively communicate with *D*. *ananassae*, suggesting that there is a generalizability to dialect learning (S23B Fig). We tested the ability of these flies to communicate with *D*. *willistoni*, as *D*. *ananassae* has an ability to communicate with *D*. *willistoni* in the naïve state, while *D*. *melanogaster* does not, allowing further analysis into the generalizability of the signal. We find that *D*. *kikkawai* trained *D*. *melanogaster* are unable to communicate *with D*. *willistoni*, suggesting that while dialect learning is generalizable in some instances, it also has a layer of specificity (S23C Fig).

### Dialect learning inputs

**Fig 6:**
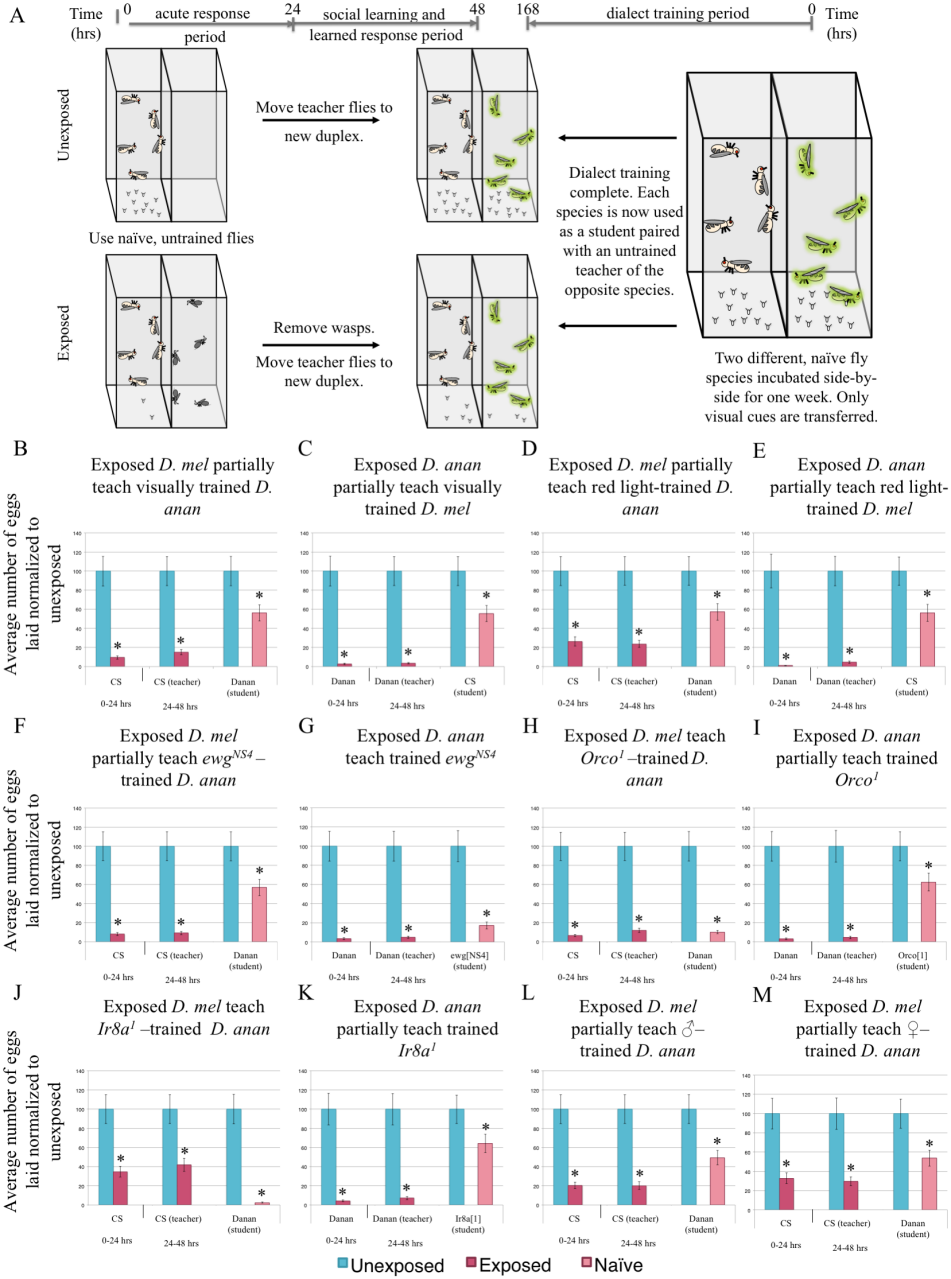
Dialect training requires multiple sensory inputs. (A) Experimental design of dialect training for flies that are used as students using only visual cues (panels B,C). Flies only see each other through the duplex, with no direct interaction. Two species are co-incubated for one week prior to being used as students. Percentage of eggs laid by exposed flies normalized to eggs laid by unexposed flies is shown. Communication between trained students *D*. *melanogaster* and *D*. *ananassae* with training through visual cues only, shows that visual cues are not sufficient (B, C). Communication between trained students *D*. *melanogaster* and *D*. *ananassae* with training through monochromatic, red light only, shows a lack of dialect training (D, E). Communication between trained students *ewg*^*NS4*^, mutant flies, and *D*. *ananassae* shows that moving wings are necessary (F, G). Communication between trained students *Orco*^*1*^ and *D*. *ananassae* shows that olfactory cues are necessary (H, I). Communication between trained students *Ir8a*^*1*^ and *D*. *ananassae* shows that *Ir8a* is a necessary receptor (J, K). Communication between trained students *D*. *melanogaster* and *D*. *ananassae* with training by male *D*. *melanogaster* only or by female *D*. *melanogaster* only, is not sufficient for dialect training (L,M). Error bars represent standard error (n = 12 biological replicates) (*p < 0.05).

In order to better understand dialect learning, we tested the roles of sensory cues and genetic factors during the dialect learning period. We measured dialect learning by quantifying improvement in interspecies partial communication between *D*. *melanogaster* and *D*. *ananassae* that normally exhibit efficient communication only after cohabitation. Given that in *D*. *melanogaster*, and in other species tested, we found visual cues to be sufficient for the teacher-student dynamic (Fig 1) [17], we asked if visual cues are sufficient and/or necessary for dialect learning. We approached this question by performing the dialect training in the fly duplex, such that the two species could only see each other (Fig 6A), or by performing the training in the dark, so that the two species could physically interact, but lacked visual cues (S24A Fig). We find that visual cues alone are not sufficient (Fig 6B and 6C), but are necessary (S24B and S24C Fig) for dialect learning. The observation that visual cues are necessary but not sufficient makes the dialect learning process different from the teacher-student dynamic that requires only visual cues[17]. Furthermore, we wondered if seeing another species altered the behavior of a fly to facilitate dialect learning. This hypothesis addresses the possibility that flies are passively acquiring information through eavesdropping and that the communication ability gained could be unidirectional. Blind *D*. *melanogaster ninaB* mutants do not function as students. Surprisingly, *D*. *ananassae* cohabitated with blind *D*. *melanogaster* do not learn the *D*. *melanogaster* dialect (S24D and S24E Fig). This result is striking because it suggests that there is an active learning component and a bidirectional exchange of information between fly species and not simply eavesdropping or mimicry. We also performed cohabitation training under two different, monochromatic light sources, and this resulted in only a partial communication between *D*. *melanogaster* and *D*. *ananassae*, (Fig 6D and 6E, S24F and S24G Fig). To exclude the possibility of a dimmer light source inhibiting dialect training under monochromatic settings, we repeated cohabitation-dialect-training in a full spectrum, lower light intensity setting, and found both species were able to learn the dialect (S24H and S24I Fig). Thus, full spectrum light is essential in dialect learning. Importantly, the observation that blind *D*. *melanogaster* do not allow wild-type *D*. *ananassae* to dialect learn suggests that visual inputs are critical to altering behavioral/chemical outputs required to facilitate dialect learning. This also suggests that during the dialect learning period, transfer of information may occur bidirectionally, if the visual input that is required is indeed provided by the other species.

Wing movement was shown to be required for teacher flies to instruct students in the teacher-student dynamic[17], raising the possibility that wing movement was also important for dialect learning. Therefore, we tested flies mutant in the erect wing gene (*ewg*), which impairs wing movement while maintaining morphologically normal wings. The allele tested has wild-type EWG protein expression in the nervous system, thus is only deficient in its non-neuronal functions, such as flight muscles [60]. We find that *D*. *ananassae* cannot dialect learn from *ewg*^*NS4*^ flies (Fig 6F), although *ewg*^*NS4*^ mutants have no dialect learning impairment (Fig 6G). This suggests that dialect learning by *D*. *ananassae* requires *D*. *melanogaster* to have mobile wings.

To test if olfactory cues play a role in dialect learning, we utilized *D*. *melanogaster* mutants defective in chemosensory signaling. The majority of olfactory receptors require a co-receptor for wild-type function, including *Orco* (Or83b) for odorant receptors [61] and *Ir8a* or *Ir25a* for ionotropic receptors [62]. Ir8a olfactory sensory neurons (OSNs) primarily detect acids and Ir25a OSNs detect amines, allowing us to probe specificity of detection. We find that *D*. *ananassae* are able to learn dialect from *Orco*^*1*^, *Ir8a*^*1*^, *Ir25a*^*2*^, single and *Ir8a*^*1*^;*Ir25a*^*2*^;*Orco*^*1*^ triple mutants and RNAi expressing *D*. *melanogaster* targeting each of these gene products (Fig 6H and 6J, S25A–S25L Fig). By contrast only *Ir25a*^*2*^ mutant and RNAi knockdown *D*. *melanogaster* were able to learn the *D*. *ananassae* dialect (Fig 6I and 6K, S25A–S25L Fig). These data demonstrate that Orco- and Ir8a-mediated olfactory inputs are required for dialect learning. This further suggests that multiple olfactory cues play important roles in the dialect learning period. We also find that *D*. *melanogaster* males and females are both required for dialect training *D*. *ananassae* (Fig 6L–6M, S25M and S25N Fig) and that the length of the training period is also critical, as 24 hours is insufficient a period for dialect learning (S25O and S25P Fig). Thus, although the exact olfactory molecule(s) critical during a dialect learning period are yet to be identified, we speculate that dialect learning is a complex process requiring visual, olfactory and sex specific cues.

**Fig 7:**
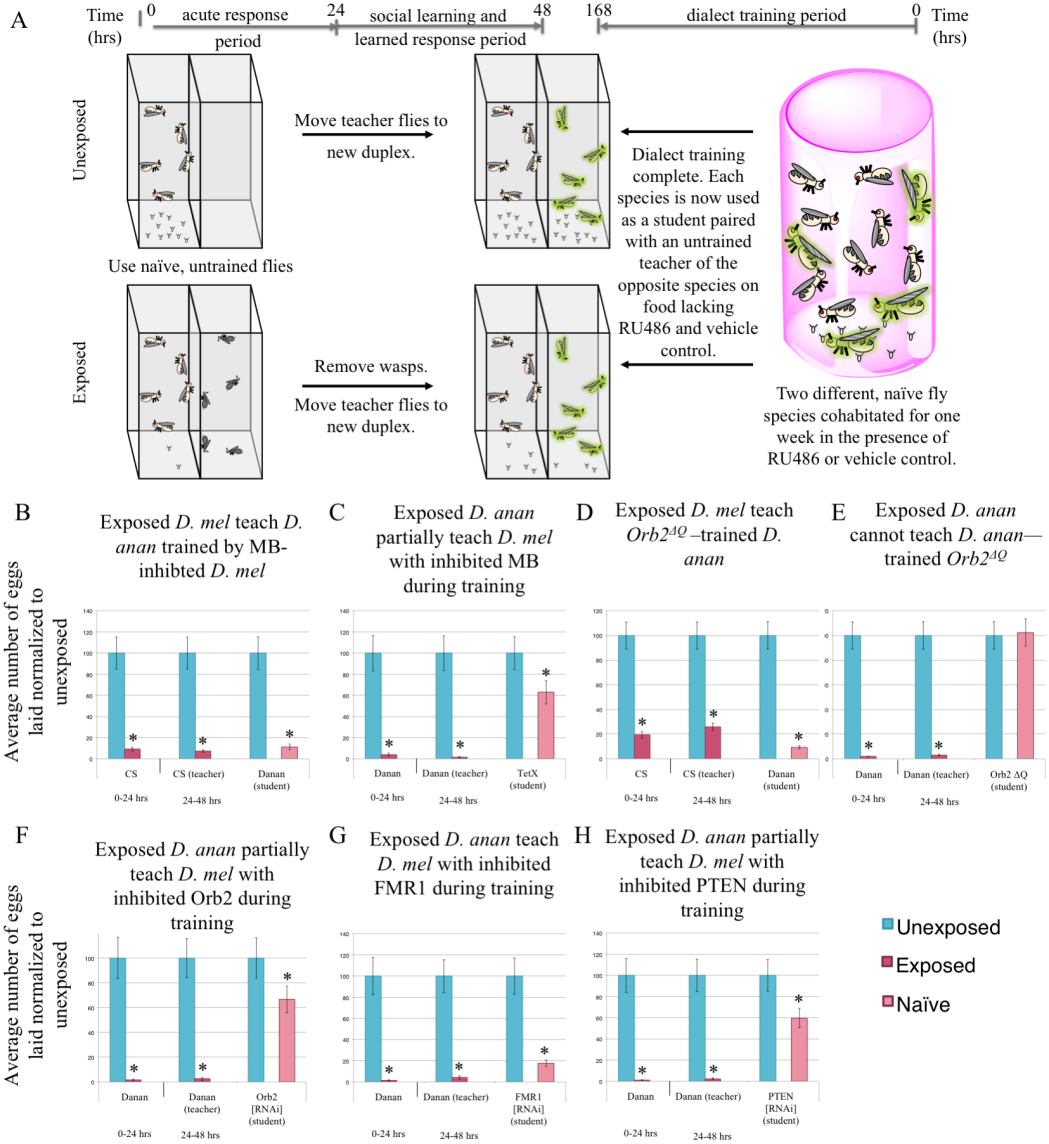
Genetic perturbations reveal a critical role of the mushroom body and memory proteins for dialect learning. (A) Experimental design of dialect training for flies being fed RU486 or methanol that are used as students. Both species are fed either RU486 or methanol during dialect training. Two species are co-incubated for one week prior to being used as students for naive, untrained teacher flies of the opposite species. Standard Drosophila media is used once the training period is over. Percentage of eggs laid by exposed flies normalized to eggs laid by unexposed flies is shown. Communication between trained students *D*. *melanogaster* and *D*. *ananassae* trained by flies expressing tetanus toxin (UAS-TeTx) in the mushroom body (MB) shows that the MB serves a critical role during the training period. *D*. *ananassae* learn from *D*. *melanogaster* with an inhibited MB, demonstrating that a functional MB is not needed to confer information during the training period (B, C). Communication between trained students *Orb2*^*ΔQ*^ and *D*. *ananassae* shows that Orb2 is required in students, but is dispensable for teachers to *D*. *ananassae* (D, E). Communication between *D*. *ananassae* and students co-incubated with *D*. *ananassae* that have RNAi-mediated Orb2 knockdown in the MB through RU486 feeding shows that the MB requires Orb2 during the training period (F). Communication between *D*. *ananassae* and students co-incubated with *D*. *ananassae* that have RNAi-mediated FMR1 knockdown (strain #24944) in the MB through RU486 feeding shows that FMR1 is not required in the MB during the training period (G). Communication between *D*. *ananassae* and students co-incubated with *D*. *ananassae* that have RNAi-mediated PTEN knockdown in the MB through RU486 feeding shows that PTEN is required in the MB during the training period (H). Error bars represent standard error (n = 12 biological replicates) (*p < 0.05).

To examine the possibility that dialect training involves active learning mediated by neurons of the mushroom body, we utilized the GAL4 Gene-Switch system to transiently express a transgene specifically in the mushroom body (MB). Using the GAL4 Gene-Switch ligand system, RU486 [63] activates the GAL4 transcription factor, while administration of the vehicle (methanol) does not [63]. RU486 was administered during the cohabitation period (or methanol for control), but not when flies were used as students, post-dialect training (Fig 7A). Feeding of RU486 to the MB switch driver line does not impair dialect learning (S26A Fig). We expressed the Tetanus toxin light chain (UAS-TeTx) specifically in the MB of *D*. *melanogaster* (to inhibit synaptic transmission during dialect training). We find that *D*. *ananassae* are able to learn the dialect of these MB inhibited flies (Fig 7B). However, *D*. *melanogaster* in which MB synaptic transmission is inhibited during the training period are unable to learn the *D*. *ananassae* dialect (Fig 7C). Control methanol-only conditions (i.e. no RU486 ligand) with flies of identical genotypes do not show this defect (S26B Fig). These data collectively indicate that visual and olfactory cues are required and possibly relayed to the MB, either directly or indirectly through a currently unknown circuit, to facilitate dialect learning. By contrast MB function does not appear to be important for *D*. *melanogaster* behavior(s) that enable *D*. *ananassae* to learn a dialect (S6B Fig). Consistent with this idea, although *Orb2*^*ΔQ*^ mutants cannot function as students (Fig 7E) [17], *D*. *ananassae* nevertheless learns the *D*. *melanogaster* dialect from *Orb2*^*ΔQ*^ mutants (Fig 7D).

Because MB function is necessary for dialect learning during dialect training, we tested the long-term memory proteins Orb2, FMR1, and phosphatase and tensin homolog (PTEN) [64,65] that are known to be required in the MB for memory formation. PTEN has been implicated in murine social learning models, though it has not been tested in a social learning assay in Drosophila [66]. We used the MB Gene-Switch to knockdown expression only during the cohabitation period, after which expression was allowed to resume. *D*. *ananassae* learn the dialect of each of these three knockdown lines, again suggesting that MB mediated processes in *D*. *melanogaster* are not necessary for *D*. *ananassae* dialect training (S26C–S26G Fig). However, under these conditions we find that functional Orb2 and PTEN are required for dialect learning in *D*. *melanogaster*, but FMR1 is dispensable (Fig 7F–7H). Orb2 and FMR1 were previously shown to be important in the teacher-student transmission of a wasp threat, and knockdown of either gene completely ablated students learning from teacher flies [17]. In this case, partial communication between *D*. *ananassae* teachers and *D*. *melanogaster* students can occur because Orb2 and PTEN expression is restored after the dialect training period, thus functioning as wild-type *D*. *melanogaster*. *D*. *melanogaster* flies having undergone knockdown of Orb2 and PTEN only during dialect training are able to function as students to wild-type *D*. *melanogaster* after the cohabitation period is completed, suggesting the partial communication phenotype observed with *D*. *ananassae* teachers is a result of gene knockdown during cohabitation and not a by-product of irreversible cellular damage or death caused by the RNAi treatments (S26H–S26L Fig). Collectively, these data show critical gene products are required to function in the MB for dialect learning during the training period. Importantly, although visual inputs are necessary MB function and active learning are not necessary in *D*. *melanogaster* in order to in turn provide cues enabling dialect learning by a wild-type *D*. *ananassae* student.

## Discussion

In this study, we present an evolutionarily conserved response to predatory wasps across the genus Drosophila, manifesting as egg laying depression coincident with an activated effector caspase, Dcp-1. These endoparasitoid wasps are ubiquitous keystone species in many ecosystems around the world, which prey on Drosophila larvae, with infections rates as high as 90% in natural populations [67–69]. We have shown that flies communicate a wasp threat through visual cues. We used a known generalist wasp species, *Leptopilina heterotoma* [70,71], suggesting that the communication observed may constitute a form of “social protection” against a pan-threat. Given the geographical distribution of this generalist wasp, species tested in this study have a high likelihood of wasp encounter [72–75]. The effects of other larval and pupal generalists, in addition to specialist wasps, are currently unknown, but may provide a fruitful avenue of study. The high infection rate and prevalence of parasitoids in nature suggest to us that other wasp strains and species may also induce intra- and interspecies communication.

**Fig 8:**
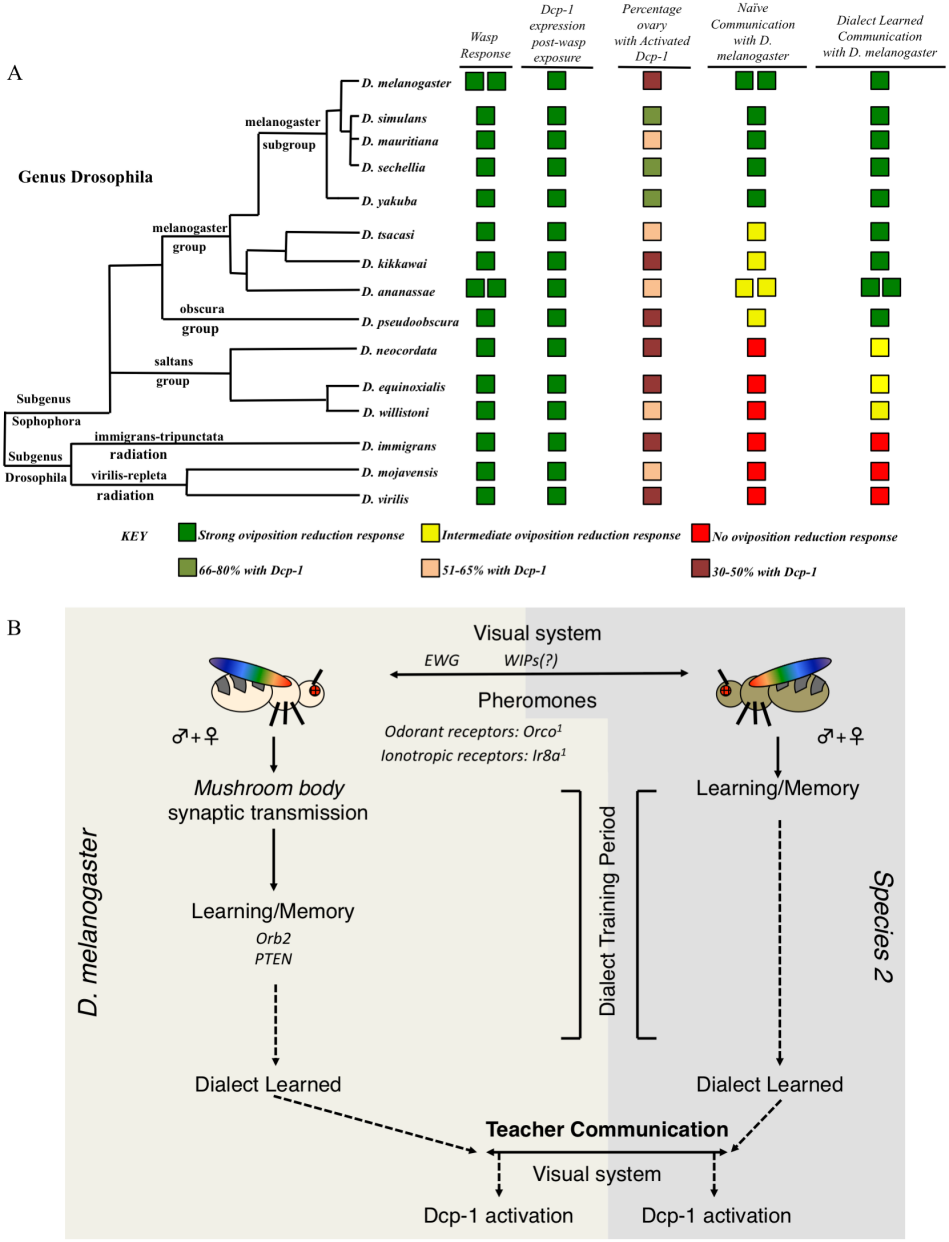
Phylogenetic summary of dialect learning and pathway model for interspecies social learning. Utilizing species across the genus Drosophila (A) demonstrates conservation of oviposition depression following wasp exposure, mediated by activated Dcp-1 to varying degrees and with varying expression patterns. The ability to communicate with *D*. *melanogaster* and the ability to demonstrate interspecies communication varies across the genus, with species closely related to *D*. *melanogaster* able to communicate without barriers. More distantly related species have difficulty communicating, though the barrier can be alleviated with dialect training. Finally, some species are too distantly related to communicate even after dialect training. Double boxes in a given row and column indicate multiple wild-type strains were tested. Interspecies communication is dependent on the presence of both male and female flies, the visual and olfactory systems, the mushroom body, and various long-term memory gene products (B). This model is based of the use of *D*. *melanogaster* and *D*. *ananasse*. Alleles tested in (B) are: Orco[1], Ir8a[1];Ir25a[2];Orco[1], Ir8a[1], Ir25a[2], ninaB[P315], Orb2ΔQ, ewg[NS4], Orb2[RNAi], PTEN[RNAi], FMR1[RNAi], and UAS-TeTx.

Interspecies communication occurs to varying degrees, likely dependent on evolutionary relatedness. Closely related species, such as *D*. *melanogaster* and *D*. *simulans*, *D*. *ananassae* and *D*. *kikkawai*, and *D*. *mojavensis* and *D*. *virilis*, communicate as effectively as conspecifics. Species more distantly related to *D*. *melanogaster* exhibit only partial communication or lack the ability to confer predator information with *D*. *melanogaster*. Because of this natural variation in ability to communicate we suggest a useful analogy to language “dialects” that may hinder efficient communication between two dissimilar dialects of a common language. When two species are only able to partially communicate, they can learn each other’s dialect after a period of cohabitation, yielding interspecies communication enhanced to levels normally observed among conspecifics. Such signals benefiting two individuals has been modeled to be honest, and evolutionarily stable [76]. Although dialect learning facilitates interspecies communication across broad evolutionary distances, the ability to learn a specific dialect is dependent on relatedness of the two species (Fig 8A). This observation of the role of phylogenetic distance influencing dialect learning is true in cases both utilizing *D*. *melanogaster* and *D*. *ananassae* in combination with other species tested (Fig 8A, S27 Fig). The observation that different strains of the same species exhibit this partial communication that can then be enhanced by cohabitation, suggests that both social communication and dialect learning are innate behaviors conserved among all Drosophilids tested here (Fig 8A, S27 Fig). Multiple strains of *D*. *melanogaster* reared in the laboratory for many decades exhibit this behavior, supporting the idea that this is an innate behavior. However, flies reared in isolation from the larval stage result in compromised communication ability, suggesting that while the ability to communicate is hardwired, or innate, there is a socialization dependent input the facilitates efficient communication, even between conspecifics. Thus, adult Drosophila neuronal plasticity allows for learning of both the communication between conspecifics and of dialects, but the specific dialect learned is dependent on social interactions specific to a communal environmental context that provides both visual and olfactory inputs. This same plasticity allows for the learning of multiple dialects in a given environment. It is remarkable that communal rearing of two species can enhance communication about a predator that is yet to be experienced by either species. Furthermore, dialect learning does not trigger Dcp-1 activation and oviposition depression, suggesting that social communication about predator presence is different from social interactions that enable dialect learning that later enhances predator presence communication.

Understanding memory formation, storage and retrieval requires knowledge of the underlying neuronal circuits. In Drosophila, the mushroom body (MB) is the major site of learning and memory and we find that the MB is necessary for dialect learning [77,78]. We hypothesize that, given the large number of inputs required in dialect learning (olfactory, ionotropic, and visual cues), which are then relayed to the MB, the “dialect” may be implemented in the MB via several neuronal classes that are activated and deactivated [77]. We suspect that there are increases in MB output neurons (MBONs) that reinforce the memory following a sufficient amount of time of stimulation (i.e. greater than 24 hours in our assay). At the same time, we suspect there may be a decrease in inhibitory MBONs that may be responsible for ignoring other species. This increase/decrease would promote interactions and learning between species. Following this MBONs changing in synaptic strength, we suspect that dopaminergic neurons (DAN(s)) reinforce these signals in the appropriate MB lobes, similar to olfactory memories in other assays. We propose this given the need for olfactory reinforcement during dialect training, in addition to other necessary cues, which emulate the known MB circuitry [77].

We propose dialect learning to be a novel behavior requiring visual and olfactory inputs, perhaps integrated in and relayed through the MB, resulting in the ability to more efficiently receive information about a common predator. Without dialect learning, this information would otherwise be lost in translation or muddled, resulting in an inefficient behavioral response with significant survival disadvantages. Inhibiting synaptic transmission and knockdown of key learning and memory genes in the MB demonstrates that these inputs must be processed and consolidated in the MB, although input neuronal signaling is initiated from the visual and olfactory systems (Fig 8B). Given the need for multiple sensory inputs, dialect learning is fundamentally different from the previously described teacher-student paradigm, where visual cues are necessary and sufficient for information exchange[17]. Additionally, we suggest that this study also points to previously unappreciated functions of the Drosophila MB in integrating information from multiple olfactory and visual inputs [77]. Such cognitive plasticity that allows for dialect learning from many different species hints that adult behaviors could only emerge in a manner that is dependent on previous social experiences where relevant ecological pressures are ever present and multiple species co-exist in nature. Thus, there is a real benefit to cognitive plasticity, where sharing of information directly, or by coincident bystanders, could result in behavioral immunity to pan-specific threats.

The specific information shared by different species during dialect learning is not known. This study, however, provides important clues as the complex suite of sensory systems and cues that may be required for efficient dialect learning. We have presented an example of how interspecies social communication and dialect learning in Drosophila can lead to changes in germline physiology and reproductive behavior. What other ethological behaviors are modulated by MB functions and social interactions typically not revealed in laboratory monocultures? We suggest that the Drosophila MB may integrate a myriad of social and environmental cues in order to produce ethologically relevant behavior that is responsive and useful to local environmental conditions.

## Materials and methods

### Insect species/strains

The *D*. *melanogaster* strains Canton-S (CS), Oregon-R (OR), white^1118^(*w*^*1118*^), and transgenic flies carrying Histone H2AvD-RFP (His-RFP) were used as wild-type strains. Experiments were primarily performed using CS as wild type flies except where otherwise indicated. *Orco*^*1*^(*Or83b*^*1*^), UAS-TeTx, UAS-Orb2^RNAi^, UAS-FMR1^RNAi^, UAS-FMR1^RNAi^, UAS-PTEN^RNAi^, UAS-Ir8a^RNAi^, UAS-Ir25a^RNAi^, *ninaB*^P315^ were acquired from the Bloomington Drosophila Stock Center (stock numbers 23129, 28838, 27050, 27484, 34944, 25841, 25813, 43985, and 24776 respectively). Drosophila species were acquired from the Drosophila Species Stock Center (DSSC) at the University of California, San Diego. Flies and their respective stock numbers are listed: *D*. *simulans* (14021–0251.196), *D*. *mauritiana* (14021–0241.01), *D*. *sechellia* (14021–0248.25), *D*. *yakuba* (14021–0261.01), *D*. *tsacasi* (14028–0701.00), *D*. *kikkawai* (14028–0561.00), *D*. *ananassae* (14024–0371.13 and 14024–0371.11), *D*. *pseudoobscura* (14011–0121.00), *D*. *neocordata* (14041–0831.00), *D*. *equinoxialis* (14030–0741.00), *D*. *willistoni* (14030–0811.00), *D*. *immigrans* (15111–1731.08), *D*. *mojavensis* (15081–1352.22), and *D*. *virilis* (15010–1051.87). All experiments with *D*. *ananassae* used strain number 14024–0371.13 unless otherwise noted (S1 Table). All stocks were kept separate to prevent visual transfer of information that could confound experiments.

The *ewg*^*NS4*^ mutant line was kindly provided by Yashi Ahmed (Geisel School of Medicine at Dartmouth). The mushroom body Gene-Switch line was kindly provided by Greg Roman (Baylor College of Medicine). *Ir8a*^*1*^, *Ir25a*^*2*^, *Ir8a>GAL4*, *Ir25a>GAL4* and *Ir8a*^*1*^;*Ir25a*^*2*^;*Orco*^*1*^ lines were kindly provided by Greg S. B. Suh (Skirball Institute at NYU). Flies aged 3–6 days post-eclosion on fresh Drosophila media were used in all experiments. Flies were maintained at room temperature with approximately 30% humidity. All species and strains used were maintained in fly bottles (Genesse catalog number 32–130) containing 50 mL of standard Drosophila media. Bottles were supplemented with 3 Kimwipes rolled together and placed into the center of the food. Drosophila media was also scored to promote oviposition. Fly species stocks were kept separate to account for visual cues that could be conferred if the stocks were kept side-by-side.

The Figitid larval endoparasitoid *Leptopilina heterotoma* (strain Lh14) was used in all experiments. *L*. *heterotoma* strain Lh14 originated from a single female collected in Winters, California in 2002. In order to propagate wasp stocks, we used adult *D*. *virilis* in batches of 40 females and 15 males per each vial (Genesse catalog number 32–116). Adult flies were allowed to lay eggs in standard Drosophila vials containing 5 mL standard Drosophila media supplemented with live yeast (approximately 25 granules) for 4–6 days before being replaced by adult wasps, using 15 female and 6 male wasps, for infections. These wasps deposit eggs in developing fly larvae, and we gave them access specifically to the L2 stage of *D*. *virilis* larvae. Wasp containing vials were supplemented with approximately 500 μL of a 50% honey/water solution applied to the inside of the cotton vial plugs. Organic honey was used as a supplement. Wasps aged 3–7 days post eclosion were used for all infections and experiments. Wasps were never reused for experiments. If wasps were used for an experiment, they were subsequently disposed of and not used to propagate the stock.

### Fly duplexes

Briefly, fly duplexes were constructed (Desco, Norfolk, MA) by using three standard 25mm x 75mm pieces of acrylic that were adhered between two 75mm x 50mm x 3mm pieces of acrylic. Clear acrylic sealant was used to glue these pieces together, making two compartments separated by one 3mm thick acrylic piece. Following sealant curing, each duplex was soaked in water and Sparkleen detergent (Fisherbrand catalog number 04-320-4) overnight, then soaked in distilled water overnight and finally air-dried. This same cleaning protocol is used following usage of a duplex. The interior dimensions of each of the two units measured approximately 23.5mm (wide) x 25mm (deep) x 75mm (tall).

For experiments using Fly Duplexes (teacher-student interaction), bead boxes (6 slot jewelers bead storage box watch part organizer sold by FindingKing) were used to accommodate 12 replicates of each treatment group. Each compartment measures 32 x 114 mm with the tray in total measuring 21 x 12 x 3.5 mm. Each compartment holds 2 duplexes, and the tray in total holds 12 duplexes. Each bead box was soaked in water and Sparkleen detergent (Fisherbrand catalog number 04-320-4) overnight, then soaked in distilled water overnight and finally air-dried every time before and after use. Empty duplexes were placed into the bead box compartments. 50 mL standard Drosophila media in a standard Drosophila bottle (Genesse catalog number 32–130) was microwaved for 39 seconds. This heated media was allowed to cool for 2 minutes on ice before being dispensed. Each duplex unit was then filled with 5 mL of the media and further allowed to cool until solidification. The open end of the Fly Duplex was plugged with a cotton plug (Genesse catalog number 51-102B) to prevent insect escape. 10 female flies and 2 male flies were placed into one chamber of the Fly Duplex in the control, while 20 female Lh14 wasps were placed next to the flies in the experimental setting for 24 hours. After the 24-hour exposure, flies and wasps were removed by anesthetizing flies and wasps in the Fly Duplexes. Control flies underwent the same anesthetization. Wasps were removed and replaced with 10 female and two male “student” flies. All flies were placed into new clean duplexes for the second 24-hour period, containing 5 mL Drosophila media in a new bead box. For fly duplexes containing a subset of species, specifically *D*. *mojavensis*, *D*. *immigrans*, and *D*. *virilis*, 10 yeast granules were added to the standard Drosophila media after solidification of the food. This activated yeast was added to promote oviposition. Flies showed minimal oviposition in food lacking yeast. We speculate this was observed due to the fly food being optimized for *D*. *melanogaster*, which could be creating sensitized species to wasp presence. Plugs used to keep insects in the duplex were replaced every 24 hours to prevent odorant deposition on plugs that could influence behavior. The oviposition bead box from each treatment was replaced 24 hours after the start of the experiment, and the second bead box was removed 48 hours after the start of the experiment. Fly egg counts from each bead box were made at the 0–24 and 24-48-hour time points. 12 biological replicates were performed except where otherwise indicated.

All experimental treatments were run at 25°C with a 12:12 light:dark cycle at light intensity 16_7_, using twelve replicates at 40% humidity unless otherwise noted. Light intensity was measured using a Sekonic L-308DC light meter. The light meter measures incident light and was set at shutter speed 120, sensitivity at iso8000, with a 1/10 step measurement value (f-stop). Fly duplexes and bead boxes soaked with distilled water mixed with Sparkleen after every use for and subsequently rinsed with distilled water and air-dried in the manner described above. To avoid bias, all egg plates were coded and scoring was blind as the individual counting eggs was not aware of treatments or genotypes/species.

### Dialect exposure

Species were cohabitated in standard Drosophila bottles (Genesee catalog number 32–130) containing 50 mL standard Drosophila media. Three Kimwipes were rolled together and placed into the center of the food. Batches of 3 bottles were made per treatment. Two species were incubated in each bottle with 100 female and 20 males of each species per bottle. Every two days, flies were placed into new bottles prepared in the identical manner. Flies were cohabitation for approximately 168 hours (7 days), unless otherwise noted. Following cohabitation, flies were anesthetized and the two species were separated. The flies were then used as students to wasp or mock exposure teachers of the opposite species. For example, we cohabitated *D*. *melanogaster* and *D*. *ananassae* for one week. Following the weeklong cohabitation, we separated the dialect trained flies. Trained *D*. *melanogaster* were placed in duplexes next to *D*. *ananassae* either mock or wasp exposed. Trained *D*. *ananassae* were placed in duplexes next to *D*. *melanogaster* either mock treated or wasp exposed.

For experiments utilizing more than two species for dialect learning, species were cohabitated in standard Drosophila bottles (Genesee catalog number 32–130) containing 50 mL standard Drosophila media. Three Kimwipes were rolled together and placed into the center of the food. Batches of 3 bottles were made per treatment. The three species were incubated in each bottle with 100 female and 20 males of each species per bottle. Every two days, flies were placed into new bottles prepared in the identical manner. The three-fly species were cohabitation for approximately 168 hours (7 days), unless otherwise noted. Following cohabitation, flies were anesthetized and one of the three species was tested by pairing them with teachers of the other two species. For example, we cohabitated *D*. *melanogaster*, *D*. *ananassae*, and *D*. *willistoni* for one week. Following the weeklong cohabitation, we separated the dialect trained flies. Trained *D*. *melanogaster* were placed in duplexes next to either *D*. *ananassae* or *D*. *willistoni*, mock or wasp exposed.

For cohabitation experiments where two species were allowed visual only cues, the Fly Duplex was utilized. The two species were co-incubated side-by-side with 100 female and 20 males of each species per unit using the two chambers of the fly duplex such that the flies could only see each other. The fly duplex was placed into bead boxes, with each unit of the duplex containing 5 mL of standard Drosophila media. Every two days, flies were placed into new fly duplexes with fresh 5 mL standard Drosophila media. Following the weeklong co-incubation, flies were anesthetized and the two species were separated. The flies were then used as students to wasp or mock exposure teachers of the opposite species.

For cohabitation experiments where the two species did not have visual cues, the two species were incubated in bottles with 100 female and 20 males of each species per bottle in complete darkness. The only difference between this method and other training sessions was the lack of light—meaning flies were subject to 25°C with 40% humidity. Every two days, flies were placed into new bottles prepared in the identical manner. Flies were exposed to light for less than 30 seconds, during which they were placed into a new bottle, and immediately returned to the dark. Following the weeklong dark-cohabitation, flies were anesthetized and the two species were separated. The flies were then used as students to wasp or mock exposure teachers of the opposite species.

For cohabitation experiments under monochromatic light settings, batches of 3 bottles with 100 female and 20 males of each species were placed into 27.9cm x 16.8cm x 13.7cm plastic boxes (Sterilite 1962 Medium Clip Box with Blue Aquarium Latches sold by Flikis). These boxes were externally wrapped with colored cellophane wrap, allowing only a certain wavelength of light to be transmitted into the boxes. Red and blue cellophane wraps were purchased from Amscam (Amscan Party Supplies for Any Occasion Functional Cellophane Wrap, 16' x 30", Rose Red and Spanish Blue). Cellophane wrapped boxes with bottles containing flies were subject to 25°C with 40% humidity under the same light intensity as previous experiments. Light intensity within the red wrapped box was 11_2_ and within the blue wrapped box was 11_5_ measured using the Sekonic L-308DC light meter. Every two days, flies were placed into new bottles prepared in the manner described previously. Flies were exposed to broad-spectrum light for less than 30 seconds, during which they were placed into a new bottle, and immediately returned to monochromatic light. Following the weeklong monochromatic-light-cohabitation, flies were anesthetized and the two species were separated. The flies were then used as students to wasp or mock exposure teachers of the opposite species.

For the one-day cohabitation experiments, batches of 3 bottles with 100 females and 20 males of each species were placed at 25°C with 40% humidity for 24 hours. Following the 24-hour cohabitation, flies were anesthetized and the two species were separated. The flies were then used as students to wasp or mock exposure teachers of the opposite species.

### Isolation experiment

In order to ask whether socialization is needed for learning ability between *D*. *melanogaster*, we performed isolation experiments (Fig 3). In order to acquire isolated flies, we performed a 24-hour egg lay using approximately 100 females and 20 males of 3-5-day old *Canton S* at 25°C with 40% humidity on grape juice agar plates. Grape juice plates were made in aliquots of 30 plates, containing a total of 100 mL. We mixed: Dextrose (5.8 g), Sucrose (3.0 g), Agar (2.2 g), and Yeast (2.2 g). We added 86 mL distilled water and 12 mL grape juice concentrate (welches brand) to these solids. This solution was brought to a boil in a microwave, and allowed to pour and solidify. Plates were used immediately upon cooling.

Following the 24-hour egg lay, flies were removed and the egg lay plate was placed at 25°C with 40% humidity with a 12:12 light: dark cycle for a second 24-hour period, after which, L1 larvae were collected and placed into a Falcon round-bottom polypropylene tube (catalog number 352063) containing 1 mL standard Drosophila media. Larvae were allowed to pupate and eclose in isolation. Each tube was kept separate such that no visual information could be transferred between tubes. Following eclosion, 3–5 day old flies were used as students. 1 female and 1 male isolated *Canton S* were used as students, paired with 1 female, 1 male *Canton S* raised under typical socialized conditions. Social conditions were achieved by performing the same egg lay protocol as above, but 100 L1 larvae were transferred to standard Drosophila bottles (Genesee catalog number 32–130) containing 50 mL standard Drosophila media and allowed to pupate and eclose at 25°C with 40% humidity.

### RU486 feeding

RU486 (Mifepristone) was used from Sigma (Lot number SLBG0210V) as the ligand for Gene-Switch experiments. Dialect training bottles were prepared by directly pipetting an RU486 solution onto the 3 Kimwipes in the bottle. The solution was prepared by dissolving 3.575 mg of RU486 in 800μL methanol (Fisher Scientific Lot number 141313). This solution was added to 15.2 mL of distilled water. The total solution (16 mL) was thoroughly mixed and 4000 μL was pipetted onto the Kimwipe in each bottle. For bottles containing no RU486 (methanol only) 800μL methanol was mixed with 15.2 mL of distilled water. The total solution (16 mL) was thoroughly mixed and 4000 μL were pipetted onto the Kimwipe in each bottle. Flies were shifted to new bottles prepared in the exact same manner every two days. Flies were cohabitated for approximately 7 days. Following cohabitation, flies were anesthetized and the two species were separated. The flies were then used as students to wasp or mock exposure teachers of the opposite species.

### Immunofluorescence

Ovaries were collected from flies that were placed in vials along with female wasps for experimental or no wasps for control settings. Flies were placed in batches into standard vials (Genesee catalog number 32–116) of 20 females, 2 males along with 20 female wasps for exposed vials, or simple placing 20 female and 2 male flies in vials for the unexposed treatments. Three vials were prepared to produce three replicates to account for batch effects. We observed no batch effects so each of the 12 ovaries imaged from each treatment were then counted as a replicate, thus providing an n of 36. Ovaries that were prepared for immunofluorescence were fixed in 4% methanol-free formaldehyde in PBS with 0.001% Triton-X for approximately five minutes. The samples were then washed in PBS with 0.1% Triton-X, and blocked with 2% normal goat serum (NGS) for two hours. The primary antibody, cleaved Drosophila Dcp-1 (Asp216) (Cell Signaling number 9578) at a concentration of 1:100, was used to incubate the ovaries overnight at 4° C in 2% normal goat serum (NGS). The secondary antibody used was Fluorescein isothiocyanate (FITC) conjugated (Jackson Immunoresearch), and used at a concentration of 1:200 for a two-hour incubation at room temperature. This was followed by a 10-minute nuclear stain with 4', 6-diamidino-2-phenylindole (DAPI). For confocal imaging of *D*. *melanogaster* ovaries, wheat germ agglutinin (WGA) was also used as a membrane marker (Fig 1F and 1J, S2 Fig). All egg chambers were counted to acquire total egg chamber number and egg chambers showing Dcp-1 signal were counted as positive for Dcp-1. All ovary quantifications were performed in a blinded manner such that the counter did not know the condition (exposed v unexposed) or species of the Drosophila ovaries being counted.

### Imaging

A Nikon A1R SI Confocal microscope was used for imaging activated Dcp-1 caspase staining in *D*. *melanogaster* (Fig 1D–1K, S2 Fig). Image averaging of 4x during image capture was used for all images. A Nikon E800 Epifluorescence microscope with Olympus DP software was used to image Dcp-1 caspase staining on all other Drosophila species tested (S3–S16 Figs). This microscope was also used to quantify egg chambers with Dcp-1 signal and total number of egg chambers in all species tested (S17 and S18 Figs).

### Statistical analysis

Statistical tests on exposed v unexposed/teacher v student interactions were performed in Microsoft Excel. Welch’s two-tailed t-tests were performed for data. P-values reported were calculated for comparisons between paired treatment-group and unexposed and are included in S1 File.

Categorization assignments were made based on the criteria of mean value and statistical significance compared to unexposed. ‘No communication’ is assigned in instances where there was not a statistically significant decrease of the exposed group. ‘Partial communication’, requires a statistically significant decrease of the exposed group, with an exposed mean above 50%. ‘Full communication’, criteria are a statistically significant decrease of the exposed group, along with a mean below 50%. Direct comparisons between partial and full communication groups would require analysis of data collected at different times and between genotypes, rendering any such p-values invalid. However, to satisfy the desire for p-values associated with the partial/full threshold, one sample one tailed t tests were performed on exposed samples that were statistically less than unexposed (S2 File). Corresponding p-values asses if the exposed group is statistically less than 50%. Statistical comparisons were performed in R (version 3.0.2 “Frisbee Sailing”).

## Supporting information

S1 Fig Intraspecies communication is present across the genus Drosophila. Percentage of eggs laid by exposed flies normalized to eggs laid by unexposed flies is shown. Species shown are (A) *D*. *melanogaster* (Oregon-R), (B) *D*. *simulans*, (C) *D*. *ananassae*, (D) *D*. *kikkawai*, (E) *D*. *willistoni*, (F) *D*. *equinoxialis*, (G) *D*. *mojavensis*, and (H) *D*. *virilis*. Error bars represent standard error (n = 12 biological replicates) (*p < 0.05).
(TIFF)

S2 Fig Activated Dcp-1 is indicative of apoptotic events in *D*. *melanogaster*. Magnified images from Fig 1(H)–1(K) showing apoptotic egg chamber displaying activated caspase. DAPI (A), activated Dcp-1 (B), WGA (C) and merge are shown (D). Additional representative ovaries of unexposed and wasp-exposed *D*. *melanogaster* are shown. DAPI (E, I, M, Q, U), activated Dcp-1 (F, J, N, R,V), WGA (G, K, O, S, W), and the merged images (H, L, P, T, X) are shown. Arrows denote apoptotic egg chambers.
(TIFF)

S3 Fig Increases in activated caspase are observed in the ovary in *D*. *simulans* following wasp exposure. Representative images of unexposed (A-F) and wasp-exposed (G-L) ovaries stained for activated Dcp-1 are shown. DAPI (A,D,G,J), Dcp-1 (B,E,H,K), and the merged images (C,F,I,L) are shown.
(TIFF)

S4 Fig Increases in activated caspase are observed in the ovary in *D*. *mauritiana* following wasp exposure. Representative images of unexposed (A-F) and wasp-exposed (G-L) ovaries stained for activated Dcp-1 are shown. DAPI (A,D,G,J), Dcp-1 (B,E,H,K), and the merged images (C,F,I,L) are shown.
(TIFF)

S5 Fig Increases in activated caspase are observed in the ovary in *D*. *sechellia* following wasp exposure. Representative images of unexposed (A-F) and wasp-exposed (G-L) ovaries stained for activated Dcp-1 are shown. DAPI (A,D,G,J), Dcp-1 (B,E,H,K), and the merged images (C,F,I,L) are shown.
(TIFF)

S6 Fig Increases in activated caspase are observed in the ovary in *D*. *yakuba* following wasp exposure. Representative images of unexposed (A-F) and wasp-exposed (G-L) ovaries stained for activated Dcp-1 are shown. DAPI (A,D,G,J), Dcp-1 (B,E,H,K), and the merged images (C,F,I,L) are shown.
(TIFF)

S7 Fig Increases in activated caspase are observed in the ovary in *D*. *tsacasi* following wasp exposure. Representative images of unexposed (A-F) and wasp-exposed (G-L) ovaries stained for activated Dcp-1 are shown. DAPI (A,D,G,J), Dcp-1 (B,E,H,K), and the merged images (C,F,I,L) are shown.
(TIFF)

S8 Fig Increases in activated caspase are observed in the ovary in *D*. *kikkawai* following wasp exposure. Representative images of unexposed (A-F) and wasp-exposed (G-L) ovaries stained for activated Dcp-1 are shown. DAPI (A,D,G,J), Dcp-1 (B,E,H,K), and the merged images (C,F,I,L) are shown.
(TIFF)

S9 Fig Increases in activated caspase are observed in the ovary in *D*. *ananassae* following wasp exposure. Representative images of unexposed (A-F) and wasp-exposed (G-L) ovaries stained for activated Dcp-1 are shown. DAPI (A,D,G,J), Dcp-1 (B,E,H,K), and the merged images (C,F,I,L) are shown.
(TIFF)

S10 Fig Increases in activated caspase are observed in the ovary in *D*. *pseudoobscura* following wasp exposure. Representative images of unexposed (A-F) and wasp-exposed (G-L) ovaries stained for activated Dcp-1 are shown. DAPI (A,D,G,J), Dcp-1 (B,E,H,K), and the merged images (C,F,I,L) are shown.
(TIFF)

S11 Fig Increases in activated caspase are observed in the ovary in *D*. *neocordata* following wasp exposure. Representative images of unexposed (A-F) and wasp-exposed (G-L) ovaries stained for activated Dcp-1 are shown. DAPI (A,D,G,J), Dcp-1 (B,E,H,K), and the merged images (C,F,I,L) are shown.
(TIFF)

S12 Fig Increases in activated caspase are observed in the ovary in *D*. *equinoxialis* following wasp exposure. Representative images of unexposed (A-F) and wasp-exposed (G-L) ovaries stained for activated Dcp-1 are shown. DAPI (A,D,G,J), Dcp-1 (B,E,H,K), and the merged images (C,F,I,L) are shown.
(TIFF)

S13 Fig Increases in activated caspase are observed in the ovary in *D*. *willistoni* following wasp exposure. Representative images of unexposed (A-F) and wasp-exposed (G-L) ovaries stained for activated Dcp-1 are shown. DAPI (A,D,G,J), Dcp-1 (B,E,H,K), and the merged images (C,F,I,L) are shown.
(TIFF)

S14 Fig Increases in activated caspase are observed in the ovary in *D*. *immigrans* following wasp exposure. Representative images of unexposed (A-F) and wasp-exposed (G-L) ovaries stained for activated Dcp-1 are shown. DAPI (A,D,G,J), Dcp-1 (B,E,H,K), and the merged images (C,F,I,L) are shown.
(TIFF)

S15 Fig Increases in activated caspase are observed in the ovary in *D*. *mojavensis* following wasp exposure. Representative images of unexposed (A-F) and wasp-exposed (G-L) ovaries stained for activated Dcp-1 are shown. DAPI (A,D,G,J), Dcp-1 (B,E,H,K), and the merged images (C,F,I,L) are shown.
(TIFF)

S16 Fig Increases in activated caspase are observed in the ovary in *D*. *virilis* following wasp exposure. Representative images of unexposed (A-F) and wasp-exposed (G-L) ovaries stained for activated Dcp-1 are shown. DAPI (A,D,G,J), Dcp-1 (B,E,H,K), and the merged images (C,F,I,L) are shown.
(TIFF)

S17 Fig Increases in activated caspase are quantified in the ovary across the genus Drosophila following wasp exposure. Proportion of egg chambers with Dcp-1 signal shown for (A) *D*. *melanogaster*, (B) *D*. *simulans*, (C) *D*. *mauritiana*, (D) *D*. *sechellia*, (E) *D*. *yakuba*, (F) *D*. *tsacasi*, (G) *D*. *kikkawai*, (H) *D*. *ananassae*, (I) *D*. *pseudoobscura*, (J) *D*. *neocordata*, (K) *D*. *equinoxialis*, (L) *D*. *willistoni*, (M) *D*. *immigrans*, (N) *D*. *mojavensis*, and (O) *D*. *virilis*. Error bars represent standard error (n = 36 ovaries) (*p < 0.05).
(TIFF)

S18 Fig A decrease in egg chamber numbers are quantified in the ovary across the genus Drosophila following wasp exposure. Total number of egg chambers shown for (A) *D*. *melanogaster*, (B) *D*. *simulans*, (C) *D*. *mauritiana*, (D) *D*. *sechellia*, (E) *D*. *yakuba*, (F) *D*. *tsacasi*, (G) *D*. *kikkawai*, (H) *D*. *ananassae*, (I) *D*. *pseudoobscura*, (J) *D*. *neocordata*, (K) *D*. *equinoxialis*, (L) *D*. *willistoni*, (M) *D*. *immigrans*, (N) *D*. *mojavensis*, and (O) *D*. *virilis*. Error bars represent standard error (n = 36 ovaries) (*p < 0.05).
(TIFF)

S19 Fig Interspecies communication of predator threats. Percentage of eggs laid by exposed flies normalized to eggs laid by unexposed flies is shown. Communication between *D*. *melanogaster* and: *D*. *sechellia* (A, B), *D*. *mauritianna* (C, D), *D*. *yakuba* (E, F), *D*. *tsacasi* (G, H), *D*. *pseudoobscura* (I, J), *D*. *neocordata* (K, L), *D*. *immigrans* (M, N), and *D*. *mojavensis* (O, P), shows varying communication abilities. Error bars represent standard error (n = 12 biological replicates except for (O), n = 24 replicates) (*p < 0.05).
(TIFF)

S20 Fig Interspecies communication of predator threats using *D*. *ananassae*. Percentage of eggs laid by exposed flies normalized to eggs laid by unexposed flies is shown. Communication between *D*. *ananassae* and: *D*. *simulans* (A, B), *D*. *kikkawai* (C, D), *D*. *equinoxialis* (E, F), *D*. *willistoni* (G, H), *D*. *mojavensis* (I, J), and *D*. *virilis* (K, L), shows varying communication abilities. Error bars represent standard error (n = 12 biological replicates) (*p < 0.05).
(TIFF)

S21 Fig Cohabitation of additional species with *D*. *melanogaster* allows for interspecies communication. Percentage of eggs laid by exposed flies normalized to eggs laid by unexposed flies is shown. Communication between trained students *D*. *melanogaster* and: *D*. *ananassae* (second line) (A-C), *D*. *tsacasi* (D, E), *D*. *pseudoobscura* (F, G), *D*. *neocordata* (H, I), *D*. *immigrans* (J, K), and *D*. *mojavensis* (L, M). (C) An additional *D*. *melanogaster* line (*w*^*1118*^) learns from *w*^*1118*^ trained *D*. *ananassae*. Communication between *D*. *ananassae* and a transgenic *D*. *melanogaster* (Histone-RFP) occurs following training period (N, O). Error bars represent standard error (n = 12 biological replicates except for (N,O), n = 24 replicates) (*p < 0.05).
(TIFF)

S22 Fig Cohabitation of additional species with *D*. *ananassae* allows for interspecies communication. Percentage of eggs laid by exposed flies normalized to eggs laid by unexposed flies is shown. Communication between trained students *D*. *ananassae* and: *D*. *simulans* (A,B), *D*. *equinoxialis* (C,D), *D*. *willistoni* (E,F), *D*. *mojavensis* (G,H), and *D*. *virilis* (I,J). Error bars represent standard error (n = 12 biological replicates) (*p < 0.05).
(TIFF)

S23 Fig Dialect is generalizable to related species, but has specificity. (A) Experimental design of testing specificity and generalizability of dialect. *D*. *melanogaster* were dialect trained with the sister species of *D*. *ananassae*, *D*. *kikkawai*. Following dialect training, *D*. *melanogaster* were used as students to either *D*. *ananassae* or *D*. *willistoni* teachers. Percentage of eggs laid by exposed flies normalized to eggs laid by unexposed flies is shown. Communication between *D*. *kikkawai—trained D*. *melanogaster* students and: *D*. *ananassae* teachers show strong communication (B), demonstrating a generalizability of the dialect signal; when paired with *D*. *willistoni* teachers, no communication is observed (C), demonstrating a signal specificity. Error bars represent standard error (n = 12 biological replicates) (*p < 0.05).
(TIFF)

S24 Fig Additional evidence demonstrating that dialect training requires visual cues. (A) Experimental design of dialect training for flies that are used as students using no visual cues by running the dialect training period in the dark (B,C). Flies do not see each other, but still interact and innervate other sensory inputs. The two species are co-incubated for one week prior to being used as students for naive, untrained teacher flies of the opposite species. Percentage of eggs laid by exposed flies normalized to eggs laid by unexposed flies is shown. Communication between trained students *D*. *melanogaster* and *D*. *ananassae* with training involving no visual cues (dark-trained), shows that visual cues necessary for dialect learning (B, C). Communication between trained students *D*. *ananassae* and the mutant *ninaB* (D,E). Communication between trained students *D*. *melanogaster* and *D*. *ananassae*, with training in monochromatic blue light only, shows a lack of dialect training (F, G). Communication between trained students of *D*. *ananassae* and *D*. *melanogaster* at 4.0_8_ light intensity shows communication (H,I). Error bars represent standard error (n = 12 biological replicates) (*p < 0.05).
(TIFF)

S25 Fig Further evidence demonstrating that dialect training requires multiple sensory inputs including olfactory cues and duration of training. Percentage of eggs laid by exposed flies normalized to eggs laid by unexposed flies is shown. Communication between naïve *D*. *ananassae* and *Ir8a*^*1*^ mutant flies shows partial communication (A). Communication between naive students of *Ir8a* knockdown in Ir8a-expressing neurons and *D*. *ananassae* shows partial communication (B). Communication between trained students *Ir8a*^*RNAi*^ knockdown in Ir8a expressing neurons and *D*. *ananassae* shows that IR8a receptor-mediated cues are necessary (C, D). Communication between naïve *Ir25a*^*2*^ mutants and *D*. *ananassae* shows partial communication (E). Communication between trained students *Ir25a*^*2*^ mutants and *D*. *ananassae* shows communication suggesting that IR25a receptors are not required for dialect training (F,G). Communication between naïve *Ir25a* knockdown in Ir25a-expressing neurons and *D*. *ananassae* shows partial communication (H). Communication between trained students *Ir25a*^*RNAi*^ knockdown in Ir25a-expressing neurons and *D*. *ananassae* shows communication suggesting that IR25a receptors are not required for dialect training (I, J). Communication between trained *Ir8a*^*1*^;*Ir25a*^*2*^;*Orco*^*1*^ students and *D*. *ananassae* shows that olfactory and IR-receptor mediated cues are necessary (K, L). Communication between students *D*. *melanogaster* and *D*. *ananassae*, with training by males only or by females only, shows partial communication, suggesting that both male and female flies are required for dialect learning (M, N). Communication between trained students *D*. *melanogaster* and *D*. *ananassae*, with training for only one day, shows that 24 hours is not sufficient for dialect training (O, P). Error bars represent standard error (n = 12 biological replicates) (*p < 0.05).
(TIFF)

S26 Fig Further evidence showing a critical role for the mushroom body and memory proteins for dialect learning. Percentage of eggs laid by exposed flies normalized to eggs laid by unexposed flies is shown. Communication between trained *D*. *melanogaster*, MBswitch/+ (outcrossed to *Canton S*) students and *D*. *ananassae* teachers fed RU486 during the training period shows communication between the two species, demonstrating that RU486 feeding does not perturb dialect learning (A). Communication between trained students *D*. *melanogaster* and *D*. *ananassae*, with training by flies not expressing tetanus toxin (UAS-TeTx) in the mushroom body (MB) (i.e. methanol fed), shows communication between the species (B). Communication between *D*. *ananassae* and students trained with *D*. *ananassae* with no RNAi-mediated Orb2 knockdown in the MB (i.e. methanol fed) shows communication between the species (C). Communication between *D*. *ananassae* and students trained with *D*. *ananassae* with RNAi-mediated FMR1 knockdown (strain #34944) in the MB (i.e. RU486 fed) shows that FMR1 is not required in the MB during the training period (D). Communication between *D*. *ananassae* and students trained with *D*. *ananassae* with no FMR1 knockdown (strain #24944) in the MB (i.e. methanol fed) shows wild-type behavior (E). Communication between *D*. *ananassae* and students trained with *D*. *ananassae* with no FMR1 knockdown (strain #34944) in the MB (i.e. methanol fed) shows wild-type behavior (F). Communication between *D*. *ananassae* and students trained with *D*. *ananassae* with no PTEN knockdown in the MB (i.e. methanol fed) shows wild-type behavior (G). Error bars represent standard error (n = 12 biological replicates) (*p < 0.05). Communication between various *D*. *melanogaster* lines trained by *D*. *ananassae* show wild-type communication with *D*. *melanogaster* (*Canton S*). Lines shown are MB switch expressing TeTx (H), Orb2^RNAi^ (I), FMR1^RNAi^ (strain number 27484) (J), FMR1^RNAi^ (strain number 34944) (K), PTEN^RNAi^ (L), and were fed RU486 during cohabitation with *D*. *ananassae*.
(TIFF)

S27 Fig Phylogenetic summary of dialect learning for *D*. *ananassae*. We utilize species across the genus Drosophila to show communication ability of *D*. *ananassae* (A). We observe the ability to demonstrate interspecies communication, which varies across the genus, with species closely related to *D*. *ananassae* able to communicate without barriers. More distantly related species have difficulty communicating, though the barrier can be alleviated with dialect training. Double boxes in a given row/column indicate multiple wild-type strains tested.
(TIFF)

S1 Table Fly lines and species used in this study. (TIFF)

S1 File Raw egg counts and p values for figures used in this study. (XLSX)

S2 File P values for all comparisons before and after dialect training. (XLSX)

## References

[1] Gould JL. Honey bee communication. Nature 1974.

[2] Wenner AM. Sound production during the waggle dance of the honey bee. Anim Behav 1962;10(1):79–95.

[3] Winston ML. The biology of the honey bee.: harvard university press; 1991.

[4] Goodale E, Beauchamp G, Magrath RD, Nieh JC, Ruxton GD. Interspecific information transfer influences animal community structure. Trends in ecology & evolution 2010;25(6):354–361. 20153073 10.1016/j.tree.2010.01.002

[5] Westrip JR, Bell MB. Breaking down the species boundaries: selective pressures behind interspecific communication in vertebrates. Ethology 2015;121(8):725–732.

[6] Elgar MA, Nash DR, Pierce NE. Eavesdropping on cooperative communication within an ant-butterfly mutualism. The Science of Nature 2016;103(9–10):84 10.1007/s00114-016-1409-5 27679457

[7] Virant-Doberlet M, Mazzoni V, De Groot M, Polajnar J, Lucchi A, Symondson WO, et al Vibrational communication networks: eavesdropping and biotic noise Studying vibrational communication: Springer; 2014 p. 93–123.

[8] Lima SL. Predators and the breeding bird: behavioral and reproductive flexibility under the risk of predation. Biological reviews 2009;84(3):485–513. 10.1111/j.1469-185X.2009.00085.x 19659887

[9] Harbison H, Nelson DA, Hahn TP. Long-term persistence of song dialects in the mountain white-crowned sparrow. Condor 1999:133–148.

[10] Koetz AH, Westcott DA, Congdon BC. Spatial pattern of song element sharing and its implications for song learning in the chowchilla, Orthonyx spaldingii. Anim Behav 2007;74(4):1019–1028.

[11] MARLER P, TAMURA M. Culturally Transmitted Patterns of Vocal Behavior in Sparrows. Science 1964 12 11;146(3650):1483–1486. 14208581 10.1126/science.146.3650.1483

[12] Soha JA, Nelson DA, Parker PG. Genetic analysis of song dialect populations in Puget Sound white-crowned sparrows. Behav Ecol 2004;15(4):636–646.

[13] Baptista LF, Morton ML. Song learning in montane white-crowned sparrows: from whom and when. Anim Behav 1988;36(6):1753–1764.

[14] Alem S, Perry CJ, Zhu X, Loukola OJ, Ingraham T, Søvik E, et al Associative mechanisms allow for social learning and cultural transmission of string pulling in an insect. PLoS Biol 2016;14(10):e1002564 10.1371/journal.pbio.1002564 27701411 PMC5049772

[15] Loukola OJ, Perry CJ, Coscos L, Chittka L. Bumblebees show cognitive flexibility by improving on an observed complex behavior. Science 2017 2 24;355(6327):833–836. 10.1126/science.aag2360 28232576

[16] Battesti M, Moreno C, Joly D, Mery F. Spread of social information and dynamics of social transmission within Drosophila groups. Current Biology 2012;22(4):309–313. 10.1016/j.cub.2011.12.050 22264604

[17] Kacsoh BZ, Bozler J, Ramaswami M, Bosco G. Social communication of predator-induced changes in Drosophila behavior and germ line physiology. Elife 2015 5 13;4: 10.7554/eLife.07423 25970035 PMC4456452

[18] Beruter J, Beauchamp GK, Muetterties EL. Complexity of chemical communication in mammals: Urinary components mediating sex discrimination by male guinea pigs. Biochem Biophys Res Commun 1973;53(1):264–271. 4741548 10.1016/0006-291x(73)91429-0

[19] Apfelbach R, Parsons MH, Soini HA, Novotny MV. Are single odorous components of a predator sufficient to elicit defensive behaviors in prey species? Front Neurosci 2015 7 29;9:263 10.3389/fnins.2015.00263 26283903 PMC4518157

[20] Voznessenskaya VV. Influence of cat odor on reproductive behavior and physiology in the house mouse (Mus musculus) Neurobiology of Chemical Communication (Frontiers in Neuroscience Book Series), Musignat-Caretta C. (Ed), CRC Press 2014:389–405. 24830030

[21] Schuchmann M, Siemers BM. Behavioral evidence for community-wide species discrimination from echolocation calls in bats. Am Nat 2010;176(1):72–82. 10.1086/652993 20459322

[22] Herzingl D, Johnsonz C. Interspecific interactions between Atlantic spotted dolphins (Stenella fr0ntalis)'and bottlenose dolphins (T ursiops truncatus) in the. Aquat Mamm 1997;23:85–99.

[23] Meinke DW, Cherry JM, Dean C, Rounsley SD, Koornneef M. Arabidopsis thaliana: a model plant for genome analysis. Science 1998;282(5389):662–682. 9784120 10.1126/science.282.5389.662

[24] Heil M, Karban R. Explaining evolution of plant communication by airborne signals. Trends in ecology & evolution 2010;25(3):137–144. 19837476 10.1016/j.tree.2009.09.010

[25] Bruin J, Dicke M, Sabelis M. Plants are better protected against spider-mites after exposure to volatiles from infested conspecifics. Experientia 1992;48(5):525–529.

[26] Karban R, Shiojiri K, Ishizaki S. An air transfer experiment confirms the role of volatile cues in communication between plants. Am Nat 2010;176(3):381–384. 10.1086/655222 20635861

[27] Karban R, Baldwin IT. Induced responses to herbivory.: University of Chicago Press; 2007.

[28] Karban R, Baldwin I, Baxter K, Laue G, Felton G. Communication between plants: induced resistance in wild tobacco plants following clipping of neighboring sagebrush. Oecologia 2000;125(1):66–71. 10.1007/PL00008892 28308223

[29] Kost C, Heil M. Herbivore‐induced plant volatiles induce an indirect defence in neighbouring plants. J Ecol 2006;94(3):619–628.

[30] RHOADES DF. Responses of alder and willow to attack by tent caterpillars and webworms: evidence for pheromonal sensitivity of willows.; 1983.

[31] Shulaev V, Silverman P, Raskin I. Airborne signalling by methyl salicylate in plant pathogen resistance.[Erratum: 4 17, 1997, v. 386 (6626), p. 738]. Nature 1997.

[32] Karban R, Shiojiri K, Huntzinger M, McCall AC. Damage-induced resistance in sagebrush: volatiles are key to intra-and interplant communication. Ecology 2006;87(4):922–930. 16676536 10.1890/0012-9658(2006)87[922:drisva]2.0.co;2

[33] Farmer EE, Ryan CA. Interplant communication: airborne methyl jasmonate induces synthesis of proteinase inhibitors in plant leaves. Proceedings of the National Academy of Sciences 1990;87(19):7713–7716. 10.1073/pnas.87.19.7713 PMC54818 11607107

[34] Glinwood R, Ninkovic V, Pettersson J, Ahmed E. Barley exposed to aerial allelopathy from thistles (Cirsium spp.) becomes less acceptable to aphids. Ecol Entomol 2004;29(2):188–195.

[35] Fowler SV, Lawton JH. Rapidly induced defenses and talking trees: the devil’s advocate position. Am Nat 1985:181–195.

[36] Helfand SL, Rogina B. Genetics of aging in the fruit fly, Drosophila melanogaster. Annu Rev Genet 2003;37(1):329–348. 14616064 10.1146/annurev.genet.37.040103.095211

[37] Bier E. Drosophila, the golden bug, emerges as a tool for human genetics. Nature Reviews Genetics 2005;6(1):9–23. 10.1038/nrg1503 15630418

[38] Markow TA. The secret lives of Drosophila flies. Elife 2015 6 4;4: 10.7554/eLife.06793 26041333 PMC4454838

[39] Markow T, Beall S, Castrezana S. The wild side of life: reproductive biology of Drosophila in nature. Fly 2012;6:98–101. 22627903 10.4161/fly.19552

[40] Markow TA. “Cost” of virginity in wild Drosophila melanogaster females. Ecology and evolution 2011;1(4):596–600. 10.1002/ece3.54 22393526 PMC3287337

[41] Markow TA. Forced Matings in Natural Populations of Drosophila. Am Nat 2000 7;156(1):100–103. 10.1086/303368 10824025

[42] Saltz JB, Foley BR. Natural genetic variation in social niche construction: social effects of aggression drive disruptive sexual selection in Drosophila melanogaster. Am Nat 2011;177(5):645–654. 10.1086/659631 21508610

[43] Sandnabba NK. Selective breeding for isolation-induced intermale aggression in mice: associated responses and environmental influences. Behav Genet 1996;26(5):477–488. 8917946 10.1007/BF02359752

[44] Sarin S, Dukas R. Social learning about egg-laying substrates in fruitflies. Proc Biol Sci 2009 12 22;276(1677):4323–4328. 10.1098/rspb.2009.1294 19759037 PMC2817106

[45] Anderson BB, Scott A, Dukas R. Social behavior and activity are decoupled in larval and adult fruit flies. Behav Ecol 2015;27(3):820–828.

[46] Simon AF, Chou M, Salazar ED, Nicholson T, Saini N, Metchev S, et al A simple assay to study social behavior in Drosophila: measurement of social space within a group. Genes, Brain and Behavior 2012;11(2):243–252. 10.1111/j.1601-183X.2011.00740.x PMC3268943 22010812

[47] Schlenke TA, Morales J, Govind S, Clark AG. Contrasting infection strategies in generalist and specialist wasp parasitoids of Drosophila melanogaster. PLoS Pathog 2007;3(10):e158. 10.1371/journal.ppat.0030158 PMC2042021 17967061

[48] Kacsoh BZ, Bozler J, Hodge S, Ramaswami M, Bosco G. A novel paradigm for nonassociative long-term memory in Drosophila: predator-induced changes in oviposition behavior. Genetics 2015 4;199(4):1143–1157. 10.1534/genetics.114.172221 25633088 PMC4391557

[49] Kacsoh BZ, Lynch ZR, Mortimer NT, Schlenke TA. Fruit flies medicate offspring after seeing parasites. Science 2013 2 22;339(6122):947–950. 10.1126/science.1229625 23430653 PMC3760715

[50] Lefevre T, de Roode JC, Kacsoh BZ, Schlenke TA. Defence strategies against a parasitoid wasp in Drosophila: fight or flight? Biol Lett 2012 4 23;8(2):230–233. 10.1098/rsbl.2011.0725 21865240 PMC3297374

[51] Lynch ZR, Schlenke TA, Roode J. Evolution of behavioural and cellular defences against parasitoid wasps in the Drosophila melanogaster subgroup. J Evol Biol 2016. 10.1111/jeb.12842 26859227

[52] Van Der Linde K, Houle D, Spicer GS, Steppan SJ. A supermatrix-based molecular phylogeny of the family Drosophilidae. Genetics research 2010;92(1):25 10.1017/S001667231000008X 20433773

[53] Ganguly-Fitzgerald I, Donlea J, Shaw PJ. Waking experience affects sleep need in Drosophila. Science 2006 9 22;313(5794):1775–1781. 10.1126/science.1130408 16990546

[54] van Swinderen B. Fly memory: a mushroom body story in parts. Current Biology 2009;19(18):R855–R857. 10.1016/j.cub.2009.07.064 19788880

[55] Farine J, Ferveur J, Everaerts C. Volatile Drosophila cuticular pheromones are affected by social but not sexual experience. PLoS One 2012;7(7):e40396 10.1371/journal.pone.0040396 22808151 PMC3394786

[56] Tauber E, Roe H, Costa R, Hennessy JM, Kyriacou CP. Temporal mating isolation driven by a behavioral gene in Drosophila. Current Biology 2003;13(2):140–145. 12546788 10.1016/s0960-9822(03)00004-6

[57] Scott AM, Dworkin I, Dukas R. Sociability in Fruit Flies: Genetic Variation, Heritability and Plasticity. Behav Genet 2018:1–12. 29682673 10.1007/s10519-018-9901-7

[58] Battesti M, Moreno C, Joly D, Mery F. Spread of social information and dynamics of social transmission within Drosophila groups. Current biology 2012;22(4):309–313. 10.1016/j.cub.2011.12.050 22264604

[59] Dombrovski M, Poussard L, Moalem K, Kmecova L, Hogan N, Schott E, et al Cooperative Behavior Emerges among Drosophila Larvae. Current Biology 2017;27(18):2821–2826. e2 10.1016/j.cub.2017.07.054 28918946

[60] DeSimone S, Coelho C, Roy S, VijayRaghavan K, White K. ERECT WING, the Drosophila member of a family of DNA binding proteins is required in imaginal myoblasts for flight muscle development. Development 1996 1;122(1):31–39. 8565844 10.1242/dev.122.1.31

[61] Larsson MC, Domingos AI, Jones WD, Chiappe ME, Amrein H, Vosshall LB. Or83b encodes a broadly expressed odorant receptor essential for Drosophila olfaction. Neuron 2004;43(5):703–714. 10.1016/j.neuron.2004.08.019 15339651

[62] Benton R, Vannice KS, Gomez-Diaz C, Vosshall LB. Variant ionotropic glutamate receptors as chemosensory receptors in Drosophila. Cell 2009;136(1):149–162. 10.1016/j.cell.2008.12.001 19135896 PMC2709536

[63] Mao Z, Roman G, Zong L, Davis RL. Pharmacogenetic rescue in time and space of the rutabaga memory impairment by using Gene-Switch. Proc Natl Acad Sci U S A 2004 1 6;101(1):198–203. 10.1073/pnas.0306128101 14684832 PMC314162

[64] Keleman K, Krüttner S, Alenius M, Dickson BJ. Function of the Drosophila CPEB protein Orb2 in long-term courtship memory. Nat Neurosci 2007;10(12):1587–1593. 10.1038/nn1996 17965711

[65] Chiang HC, Wang L, Xie Z, Yau A, Zhong Y. PI3 kinase signaling is involved in Abeta-induced memory loss in Drosophila. Proc Natl Acad Sci U S A 2010 4 13;107(15):7060–7065. 10.1073/pnas.0909314107 20351282 PMC2872421

[66] Silverman JL, Yang M, Lord C, Crawley JN. Behavioural phenotyping assays for mouse models of autism. Nature Reviews Neuroscience 2010;11(7):490–502. 10.1038/nrn2851 20559336 PMC3087436

[67] Fleury F, Ris N, Allemand R, Fouillet P, Carton Y, Boulétreau M. Ecological and Genetic Interactions in Drosophila–parasitoids Communities: A Case Study with D. Melanogaster, D. Simulans and their Common Leptopilina Parasitoids in Southe-astern France. Genetica 2004;120(1–3):181–194. 15088657 10.1023/b:gene.0000017640.78087.9e

[68] Driessen G, Hemerik L, Van Alphen JJ. Drosophila species, breeding in the stinkhorn (Phallus impudicus Pers.) and their larval parasitoids. Neth J Zool 1989;40(3):409–427.

[69] Janssen A, Driessen G, De Haan M, Roodbol N. The impact of parasitoids on natural populations of temperate woodland Drosophila. Neth J Zool 1987;38(1):61–73.

[70] Kacsoh BZ, Schlenke TA. High hemocyte load is associated with increased resistance against parasitoids in Drosophila suzukii, a relative of D. melanogaster. PloS one 2012;7(4):e34721 10.1371/journal.pone.0034721 22529929 PMC3328493

[71] Schlenke TA, Morales J, Govind S, Clark AG. Contrasting infection strategies in generalist and specialist wasp parasitoids of Drosophila melanogaster. PLoS Pathog 2007;3(10):e158. 10.1371/journal.ppat.0030158 PMC2042021 17967061

[72] Mitsui H, Van Achterberg K, Nordlander G, Kimura MT. Geographical distributions and host associations of larval parasitoids of frugivorous Drosophilidae in Japan. J Nat Hist 2007;41(25–28):1731–1738.

[73] Fleury F, Ris N, Allemand R, Fouillet P, Carton Y, Boulétreau M. Ecological and genetic interactions in Drosophila-parasitoids communities: a case study with D. melanogaster, D. simulans and their common Leptopilina parasitoids in south-eastern France Drosophila melanogaster, Drosophila simulans: So Similar, So Different: Springer; 2004 p. 181–194. 10.1023/b:gene.0000017640.78087.9e 15088657

[74] Kraaijeveld AR, Van Alphen JJ. Geographical variation in encapsulation ability ofDrosophila melanogaster larvae and evidence for parasitoid-specific components. Evol Ecol 1995;9(1):10–17.

[75] Kraaijeveld AR, Godfray HCJ. Geographic patterns in the evolution of resistance and virulence in Drosophila and its parasitoids. Am Nat 1999;153(S5):S61–S74. 10.1086/303212 29578778

[76] Szamado S. Honesty needs no cost: beneficial signals can be honest and evolutionarily stable. bioRxiv 2018:256248.

[77] Takemura SY, Aso Y, Hige T, Wong A, Lu Z, Xu CS, et al A connectome of a learning and memory center in the adult Drosophila brain. Elife 2017 7 18;6: 10.7554/eLife.26975 28718765 PMC5550281

[78] Aso Y, Hattori D, Yu Y, Johnston RM, Iyer NA, Ngo TT, et al The neuronal architecture of the mushroom body provides a logic for associative learning. Elife 2014 12 23;3:e04577 10.7554/eLife.04577 25535793 PMC4273437

