# Correction: Drosophila species learn dialects through communal living

**DOI:** 10.1371/journal.pgen.1007825

**Published:** 2018-11-27

**Authors:** Balint Z. Kacsoh, Julianna Bozler, Giovanni Bosco

There is an instance of data duplication in the Supporting Information item S1 File. The underlying numerical data supplied for Figs 2A and 2B are identical.

Please view the corrected version of S1 File below.

## Supporting information

S1 File Raw egg counts and p values for figures used in this study. (XLSX)
