# Supplementary figures and images for: Drosophila species learn dialects through communal living
Source: PLoS Genet. 2018 Jul 19;14(7):e1007430. doi: 10.1371/journal.pgen.1007430 (PMC6053138; doi:10.1371/journal.pgen.1007430)

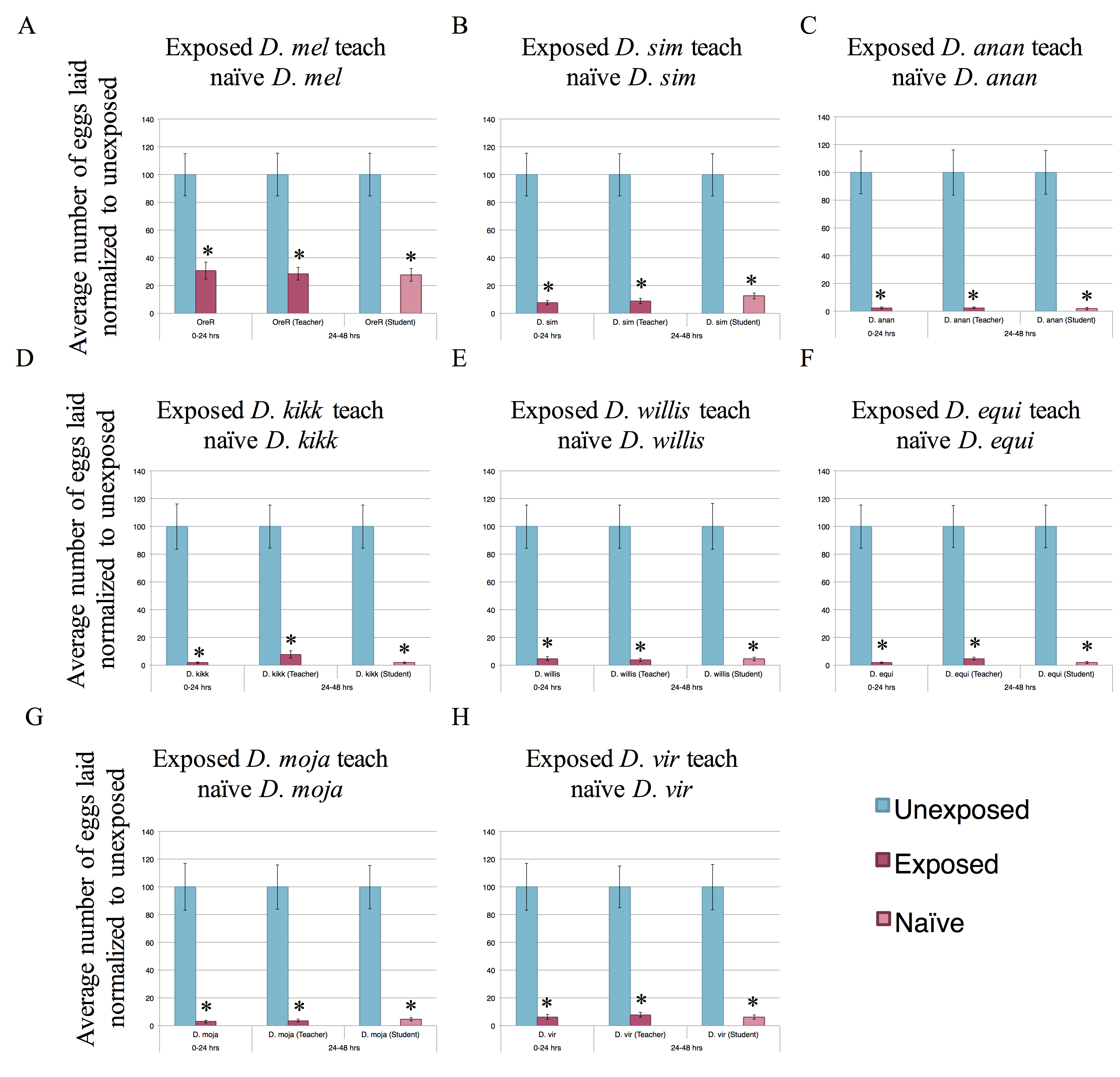

Supplement: S1 Fig — Percentage of eggs laid by exposed flies normalized to eggs laid by unexposed flies is shown. Species shown are (A) D. melanogaster (Oregon-R), (B) D. simulans, (C) D. ananassae, (D) D. kikkawai, (E) D. willistoni, (F) D. equinoxialis, (G) D. mojavensis, and (H) D. virilis. Error bars represent standard error (n = 12 biological replicates) (*p < 0.05). (TIFF) [file pgen.1007430.s001.tiff]

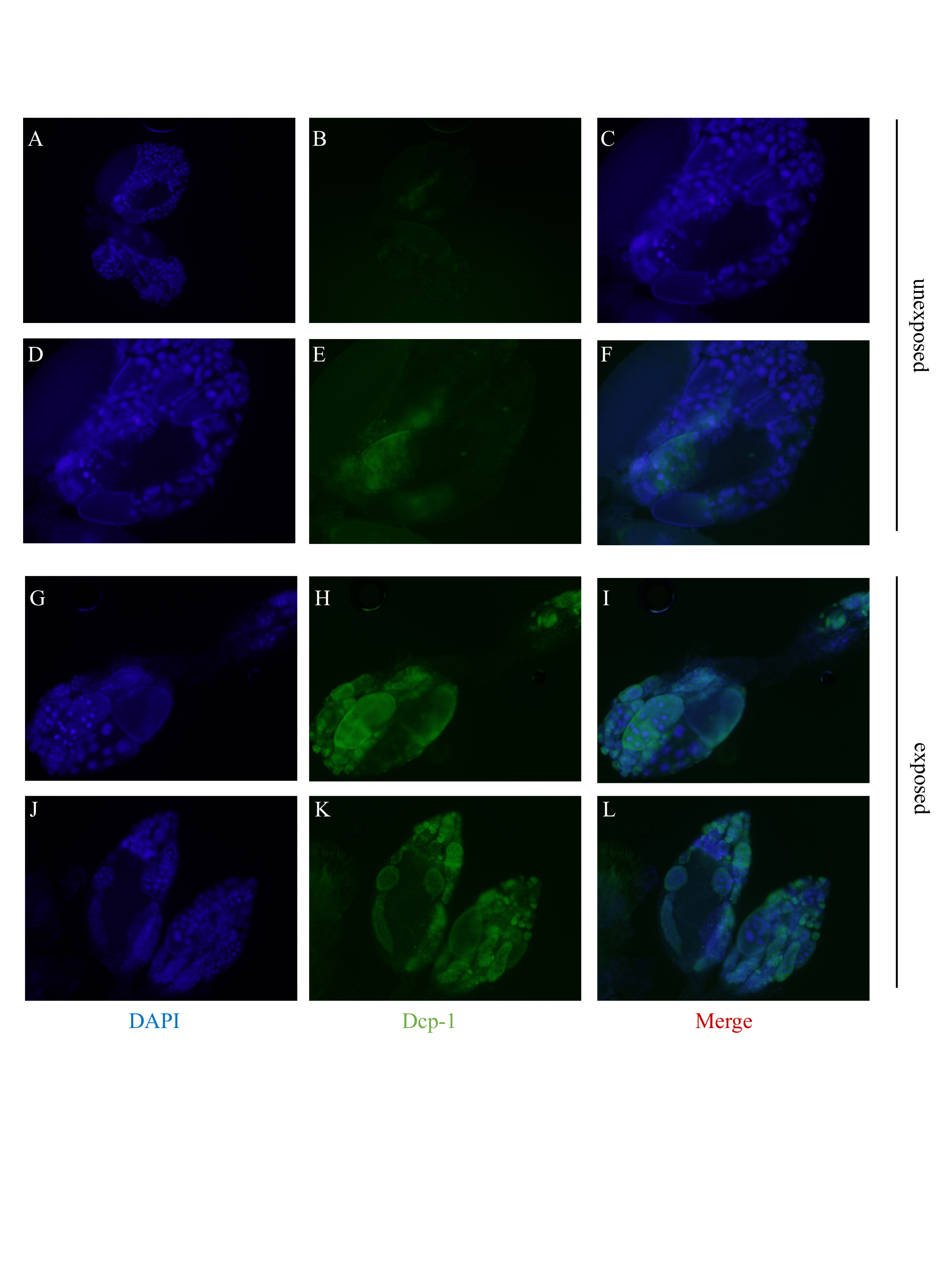

Supplement: S3 Fig — Representative images of unexposed (A-F) and wasp-exposed (G-L) ovaries stained for activated Dcp-1 are shown. DAPI (A,D,G,J), Dcp-1 (B,E,H,K), and the merged images (C,F,I,L) are shown. (TIFF) [file pgen.1007430.s003.tiff]

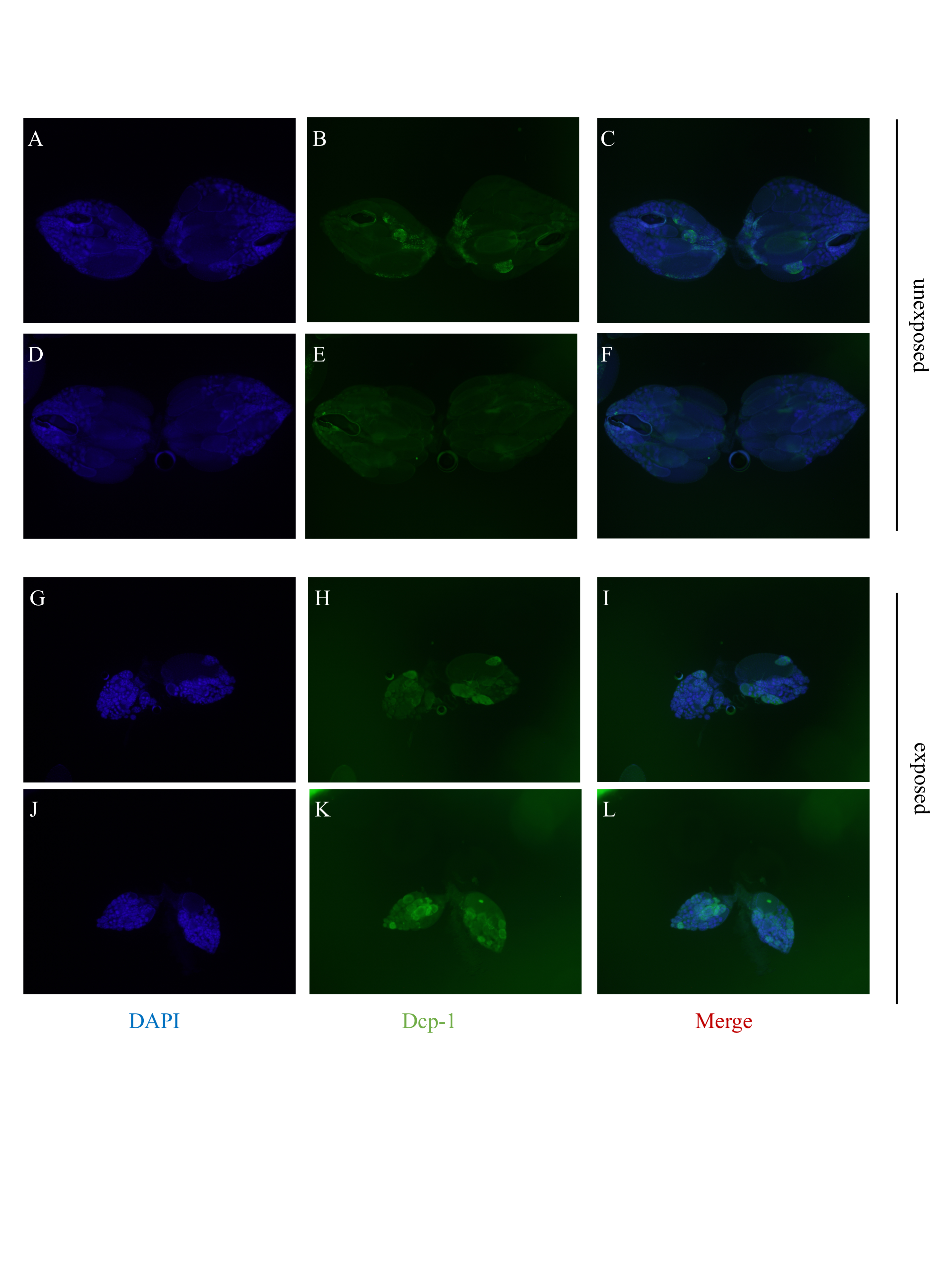

Supplement: S4 Fig — Representative images of unexposed (A-F) and wasp-exposed (G-L) ovaries stained for activated Dcp-1 are shown. DAPI (A,D,G,J), Dcp-1 (B,E,H,K), and the merged images (C,F,I,L) are shown. (TIFF) [file pgen.1007430.s004.tiff]

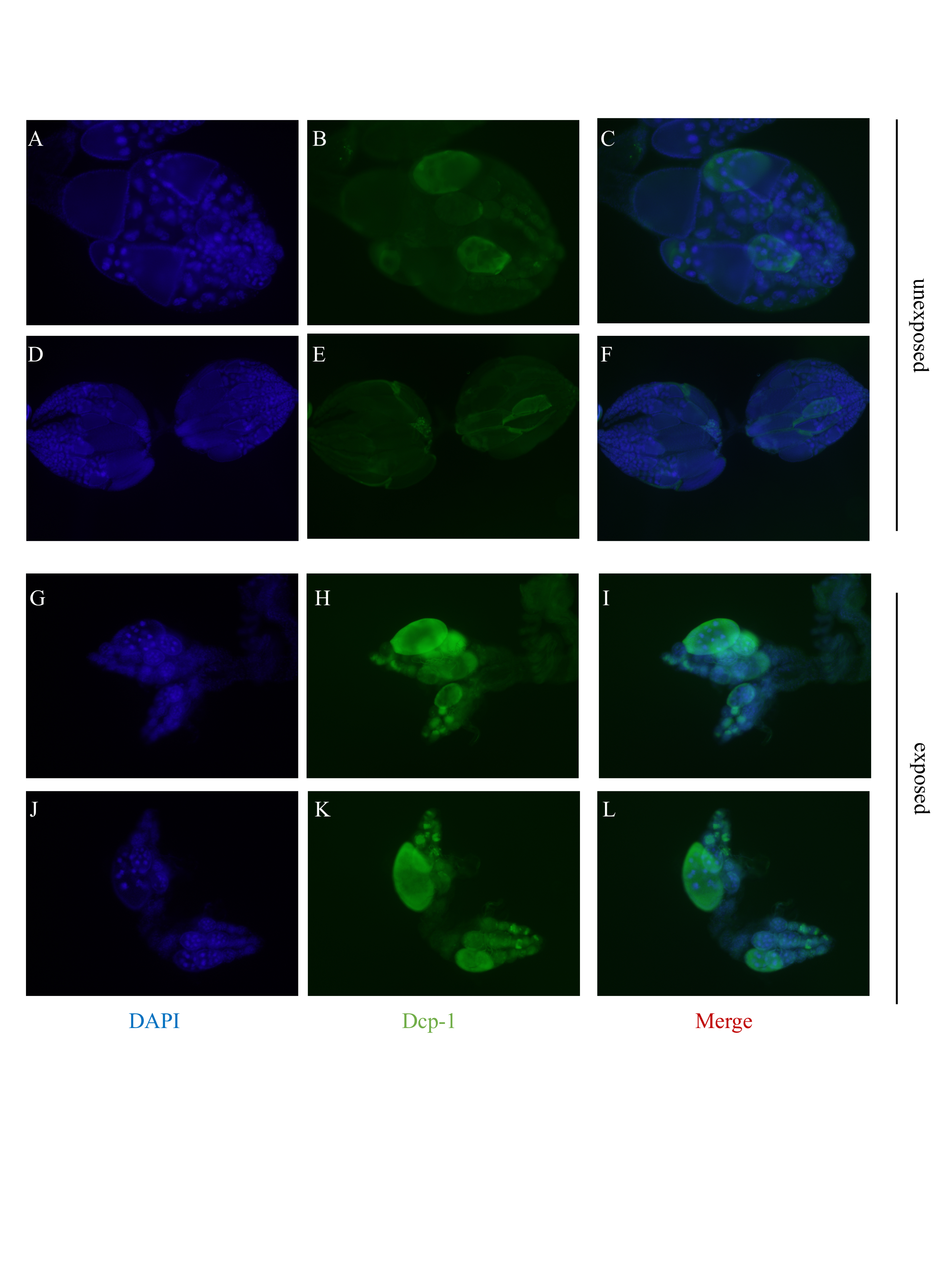

Supplement: S5 Fig — Representative images of unexposed (A-F) and wasp-exposed (G-L) ovaries stained for activated Dcp-1 are shown. DAPI (A,D,G,J), Dcp-1 (B,E,H,K), and the merged images (C,F,I,L) are shown. (TIFF) [file pgen.1007430.s005.tiff]

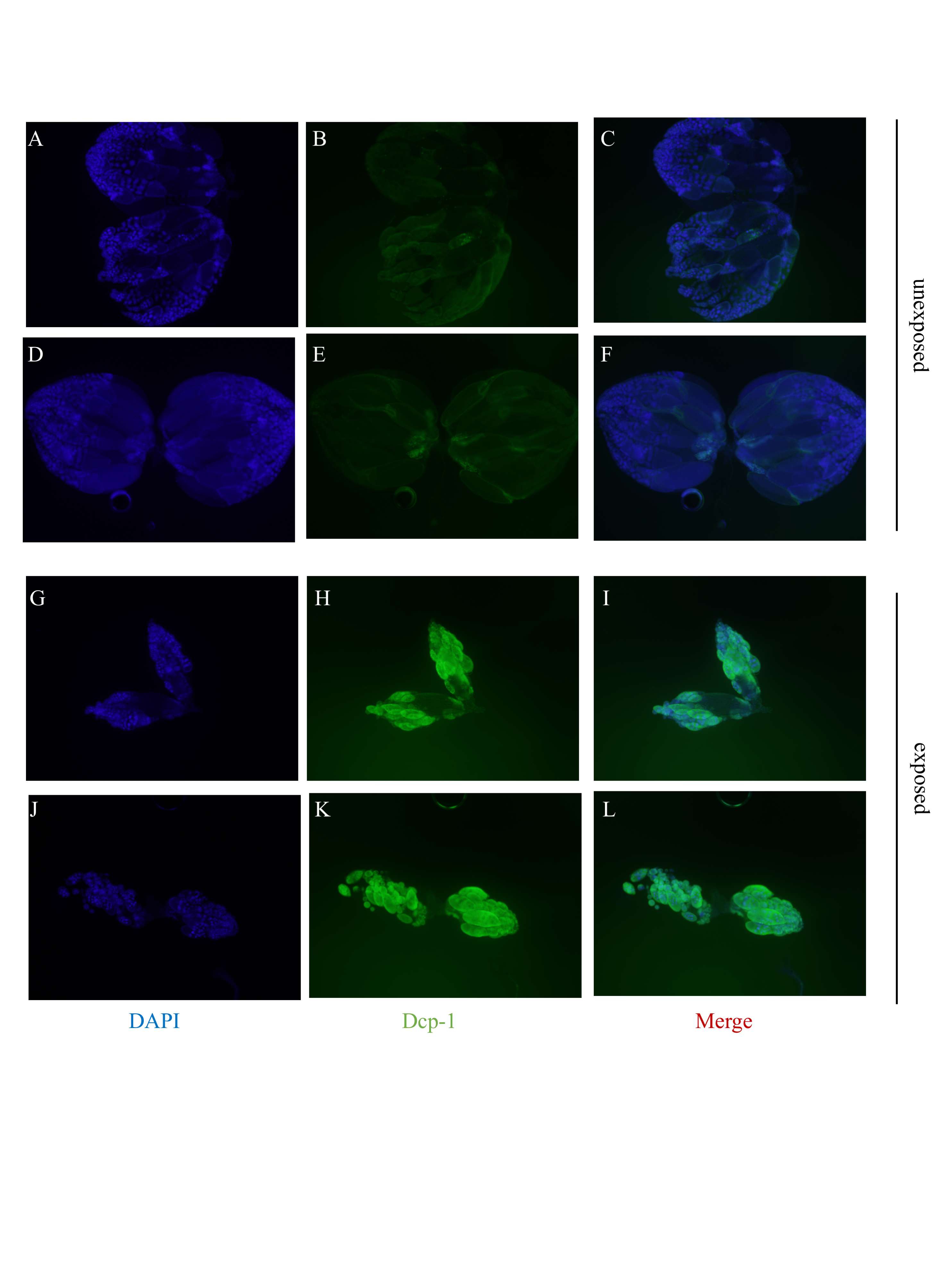

Supplement: S6 Fig — Representative images of unexposed (A-F) and wasp-exposed (G-L) ovaries stained for activated Dcp-1 are shown. DAPI (A,D,G,J), Dcp-1 (B,E,H,K), and the merged images (C,F,I,L) are shown. (TIFF) [file pgen.1007430.s006.tiff]

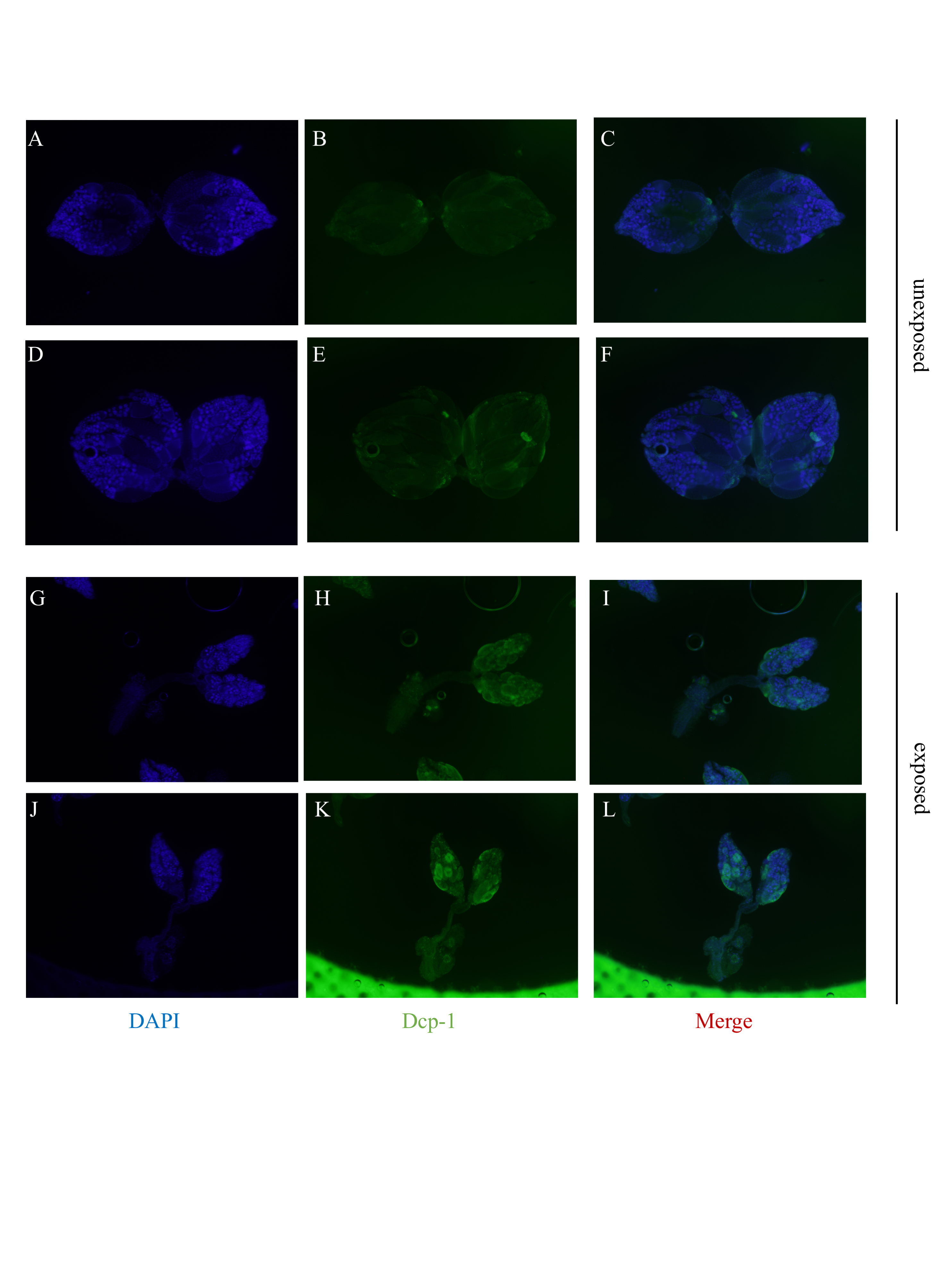

Supplement: S7 Fig — Representative images of unexposed (A-F) and wasp-exposed (G-L) ovaries stained for activated Dcp-1 are shown. DAPI (A,D,G,J), Dcp-1 (B,E,H,K), and the merged images (C,F,I,L) are shown. (TIFF) [file pgen.1007430.s007.tiff]

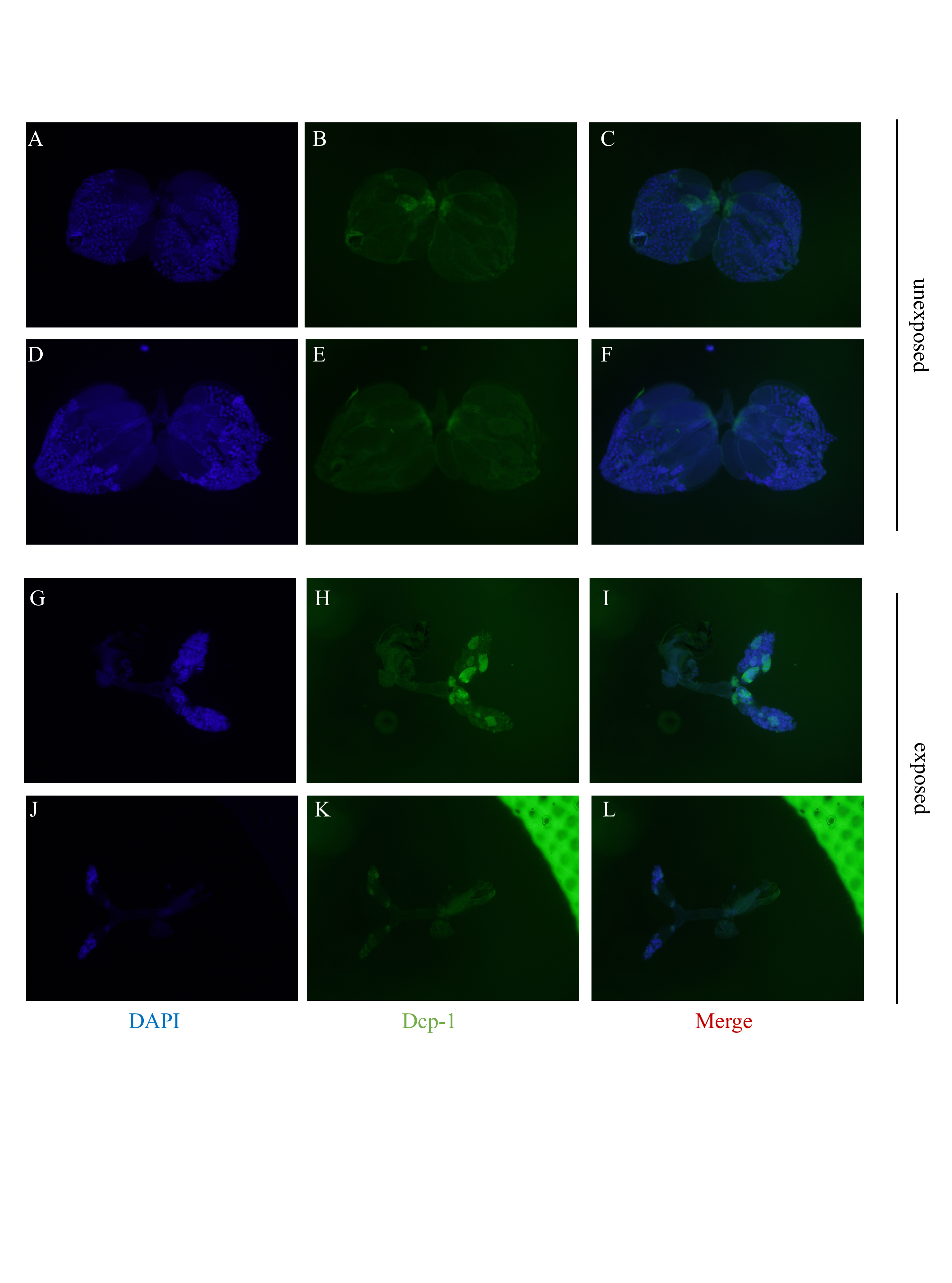

Supplement: S8 Fig — Representative images of unexposed (A-F) and wasp-exposed (G-L) ovaries stained for activated Dcp-1 are shown. DAPI (A,D,G,J), Dcp-1 (B,E,H,K), and the merged images (C,F,I,L) are shown. (TIFF) [file pgen.1007430.s008.tiff]

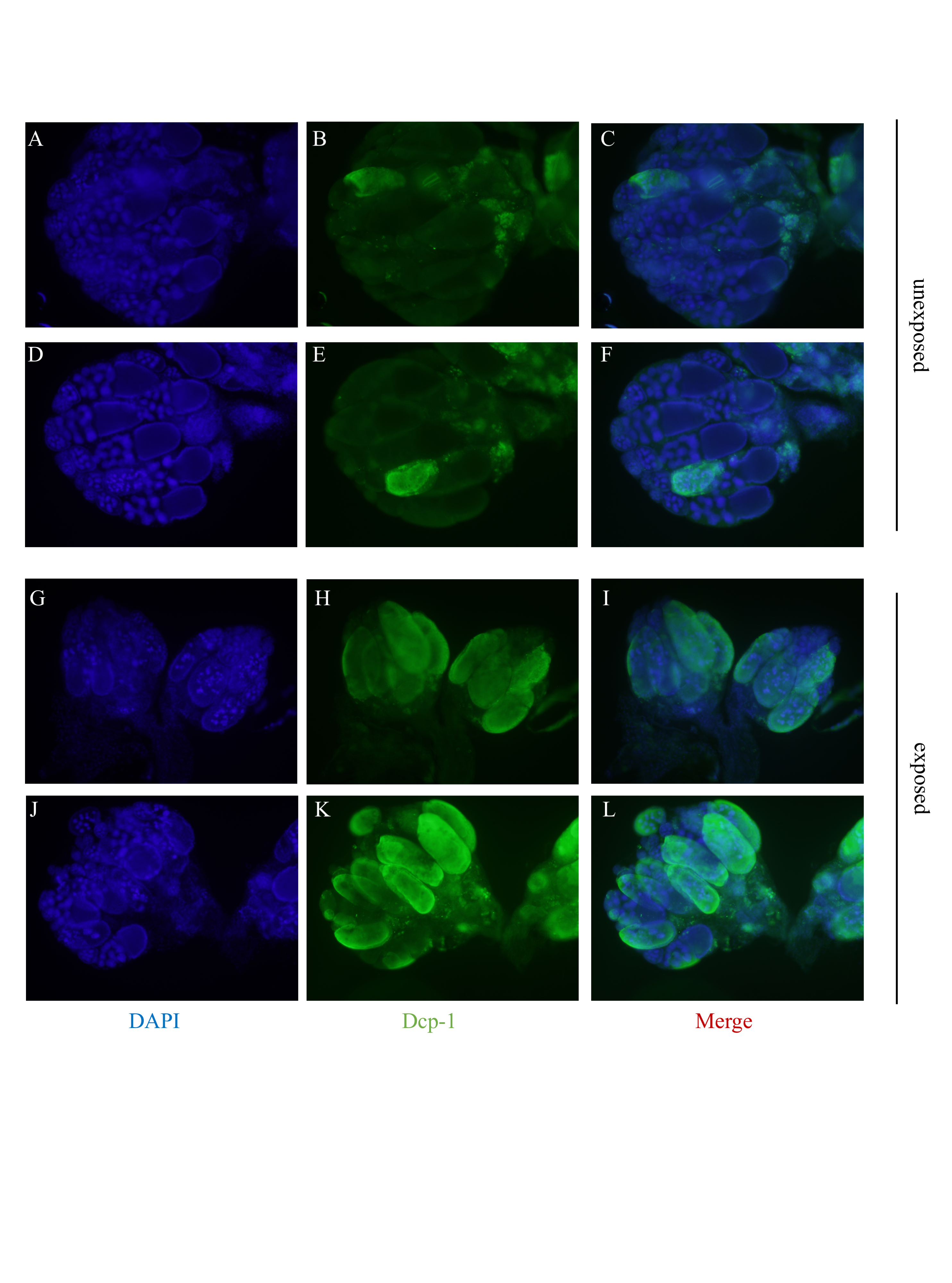

Supplement: S9 Fig — Representative images of unexposed (A-F) and wasp-exposed (G-L) ovaries stained for activated Dcp-1 are shown. DAPI (A,D,G,J), Dcp-1 (B,E,H,K), and the merged images (C,F,I,L) are shown. (TIFF) [file pgen.1007430.s009.tiff]

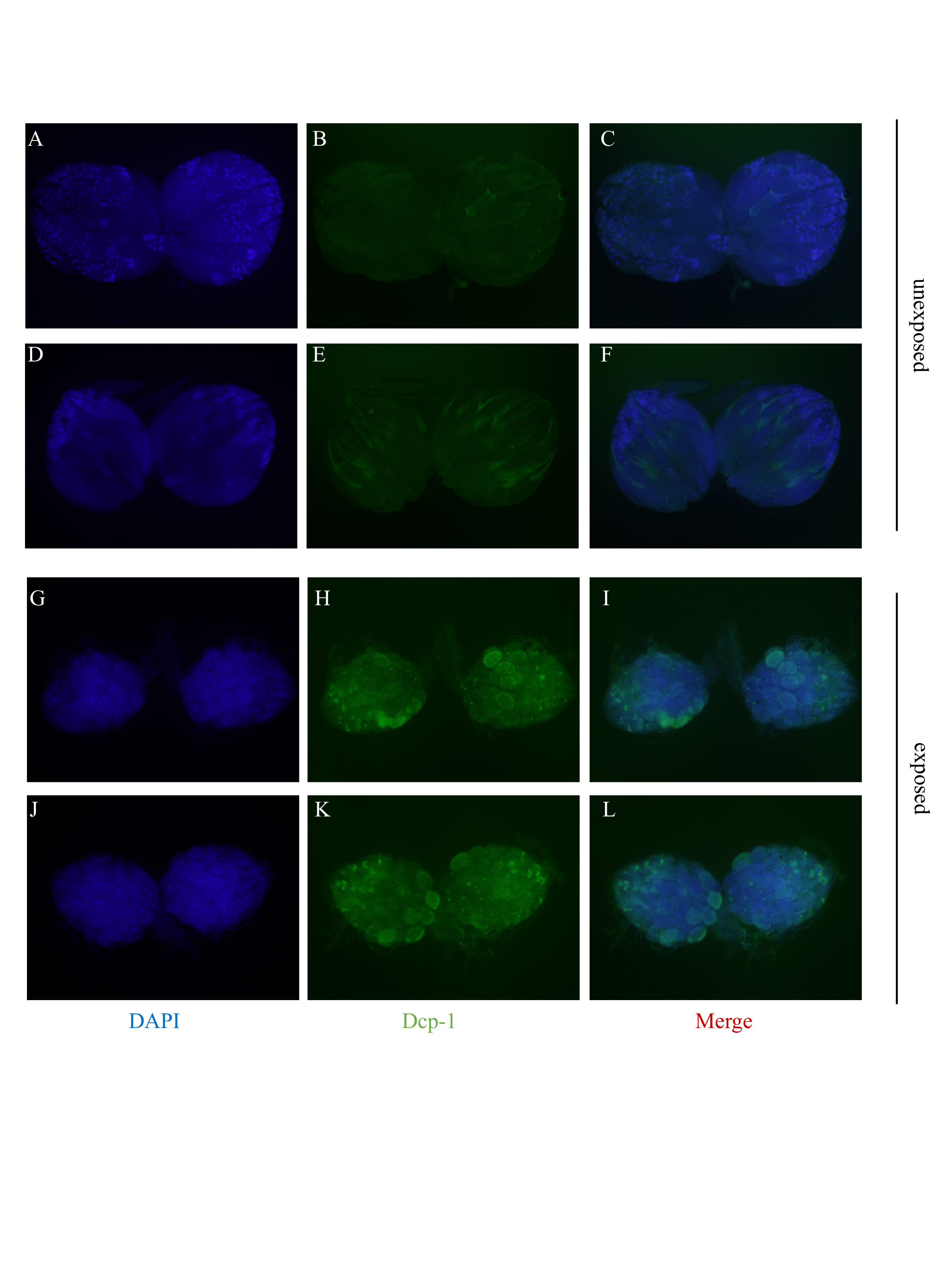

Supplement: S10 Fig — Representative images of unexposed (A-F) and wasp-exposed (G-L) ovaries stained for activated Dcp-1 are shown. DAPI (A,D,G,J), Dcp-1 (B,E,H,K), and the merged images (C,F,I,L) are shown. (TIFF) [file pgen.1007430.s010.tiff]

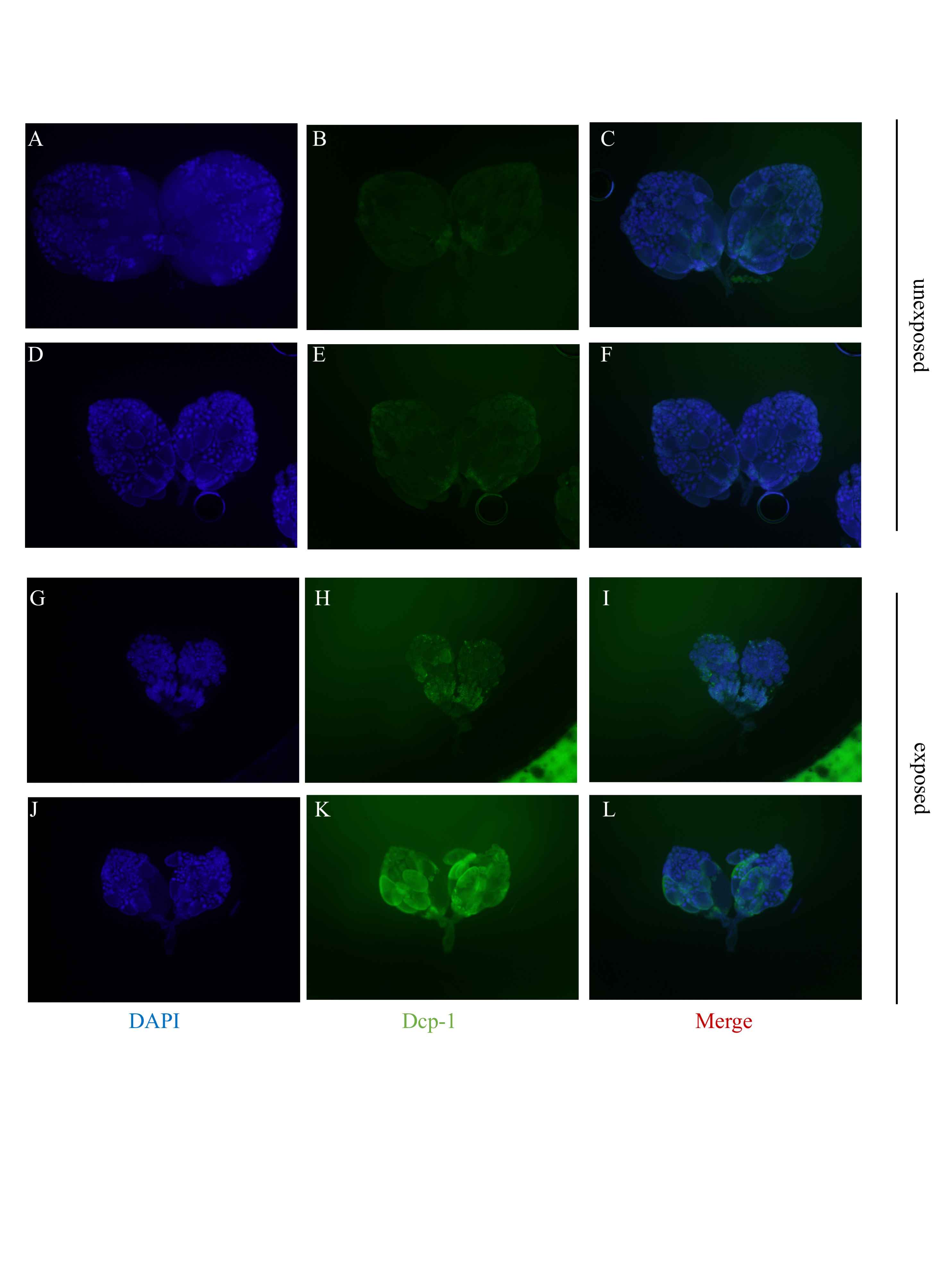

Supplement: S11 Fig — Representative images of unexposed (A-F) and wasp-exposed (G-L) ovaries stained for activated Dcp-1 are shown. DAPI (A,D,G,J), Dcp-1 (B,E,H,K), and the merged images (C,F,I,L) are shown. (TIFF) [file pgen.1007430.s011.tiff]

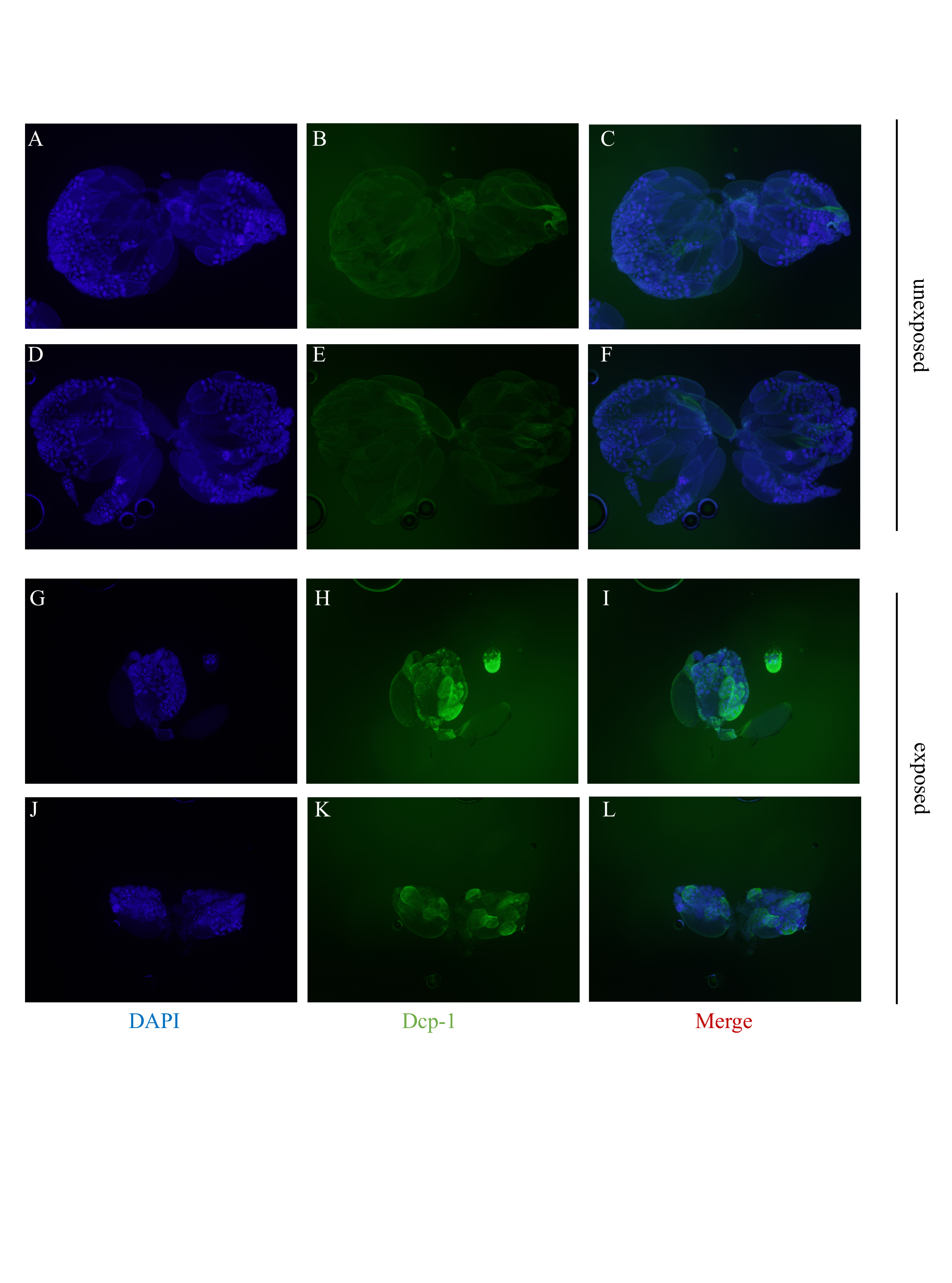

Supplement: S12 Fig — Representative images of unexposed (A-F) and wasp-exposed (G-L) ovaries stained for activated Dcp-1 are shown. DAPI (A,D,G,J), Dcp-1 (B,E,H,K), and the merged images (C,F,I,L) are shown. (TIFF) [file pgen.1007430.s012.tiff]

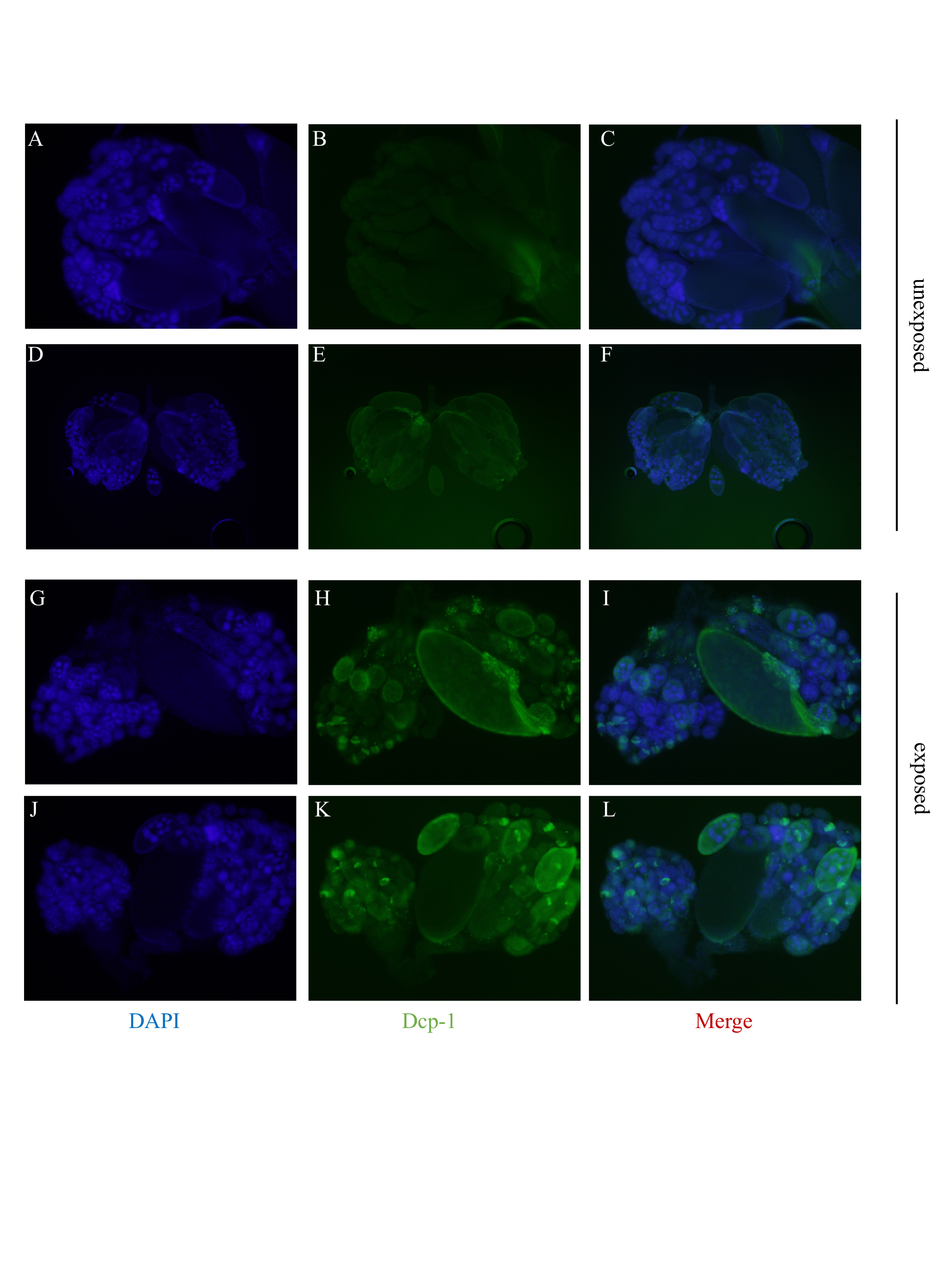

Supplement: S13 Fig — Representative images of unexposed (A-F) and wasp-exposed (G-L) ovaries stained for activated Dcp-1 are shown. DAPI (A,D,G,J), Dcp-1 (B,E,H,K), and the merged images (C,F,I,L) are shown. (TIFF) [file pgen.1007430.s013.tiff]

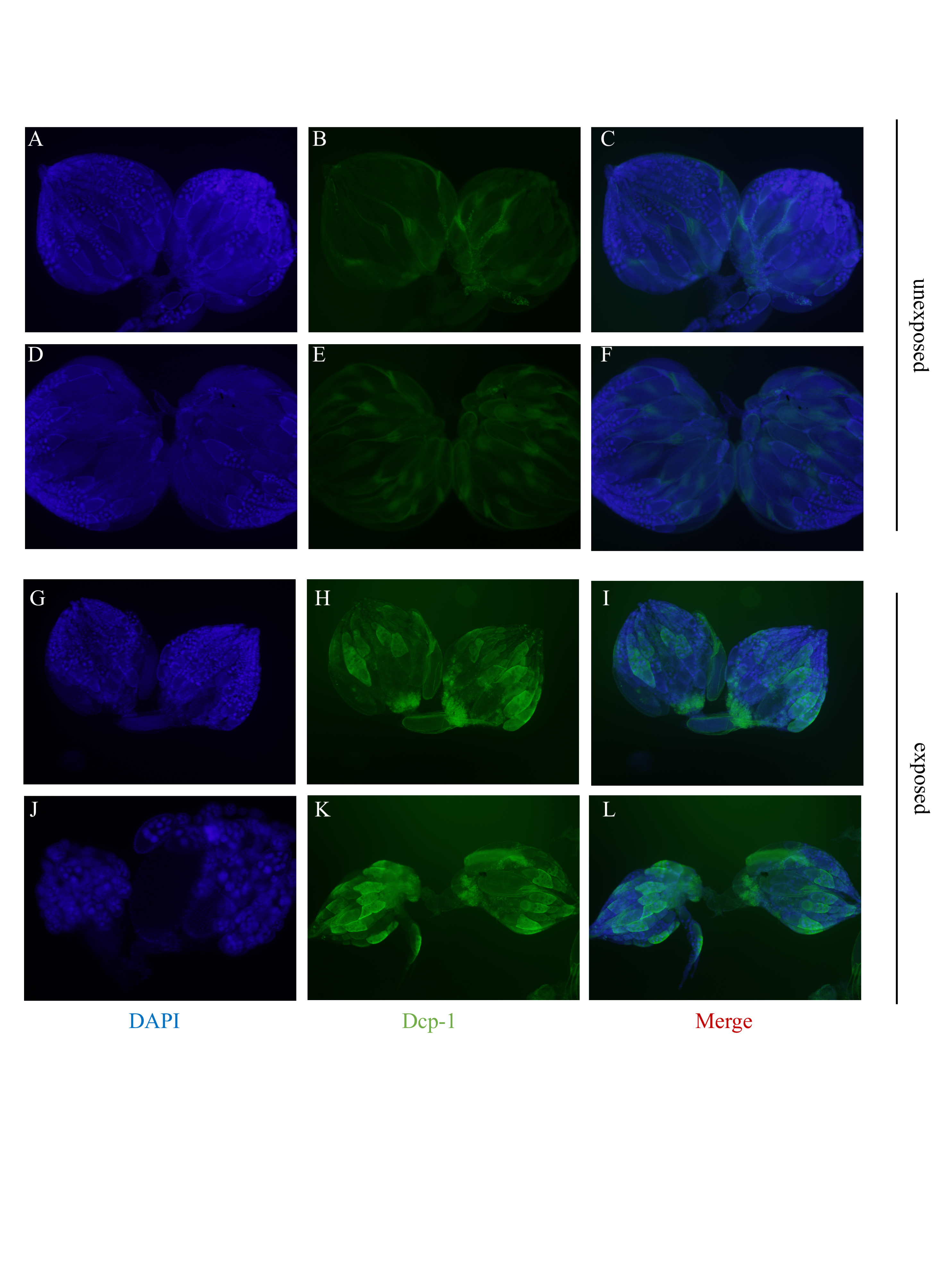

Supplement: S14 Fig — Representative images of unexposed (A-F) and wasp-exposed (G-L) ovaries stained for activated Dcp-1 are shown. DAPI (A,D,G,J), Dcp-1 (B,E,H,K), and the merged images (C,F,I,L) are shown. (TIFF) [file pgen.1007430.s014.tiff]

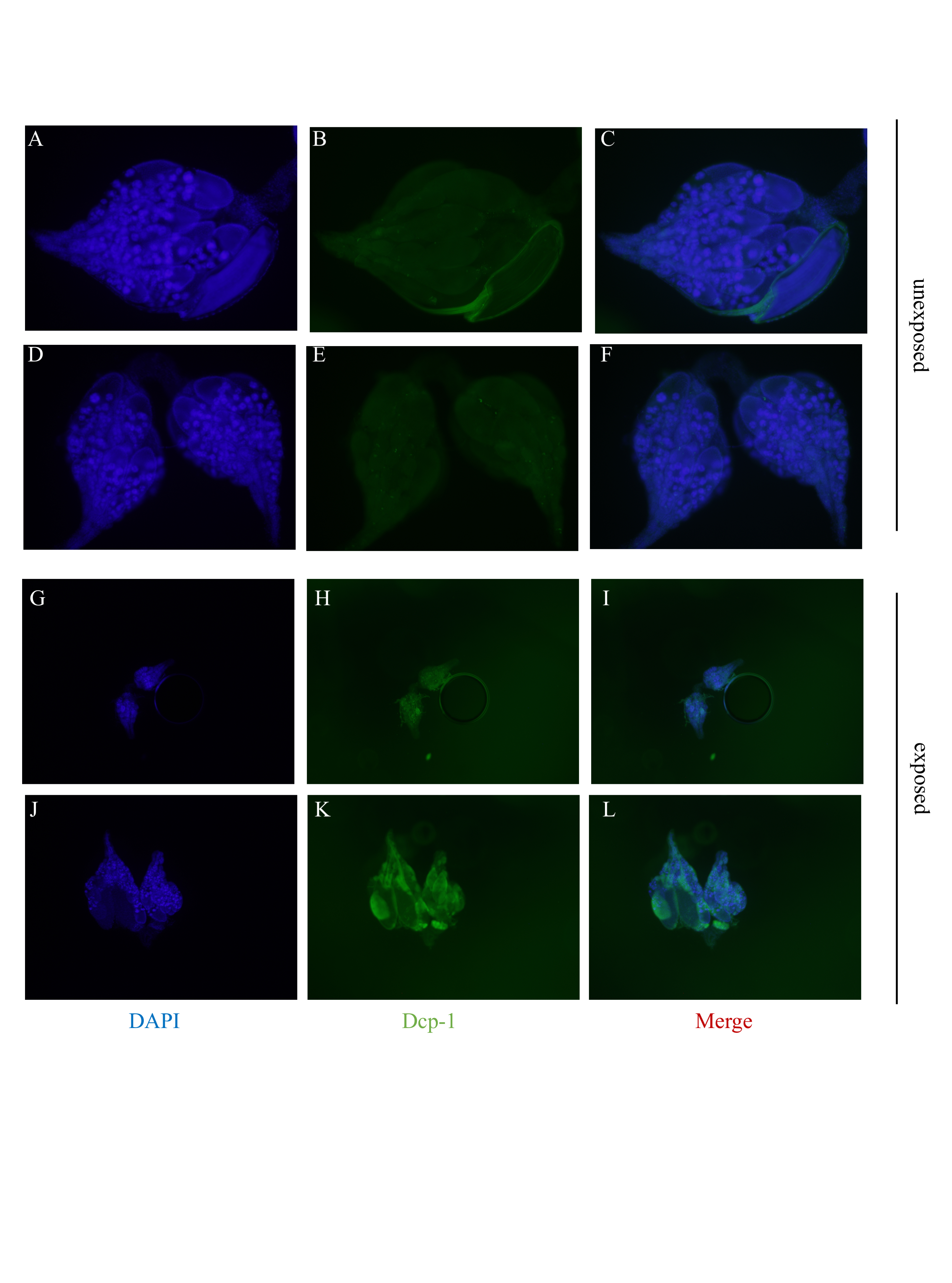

Supplement: S15 Fig — Representative images of unexposed (A-F) and wasp-exposed (G-L) ovaries stained for activated Dcp-1 are shown. DAPI (A,D,G,J), Dcp-1 (B,E,H,K), and the merged images (C,F,I,L) are shown. (TIFF) [file pgen.1007430.s015.tiff]

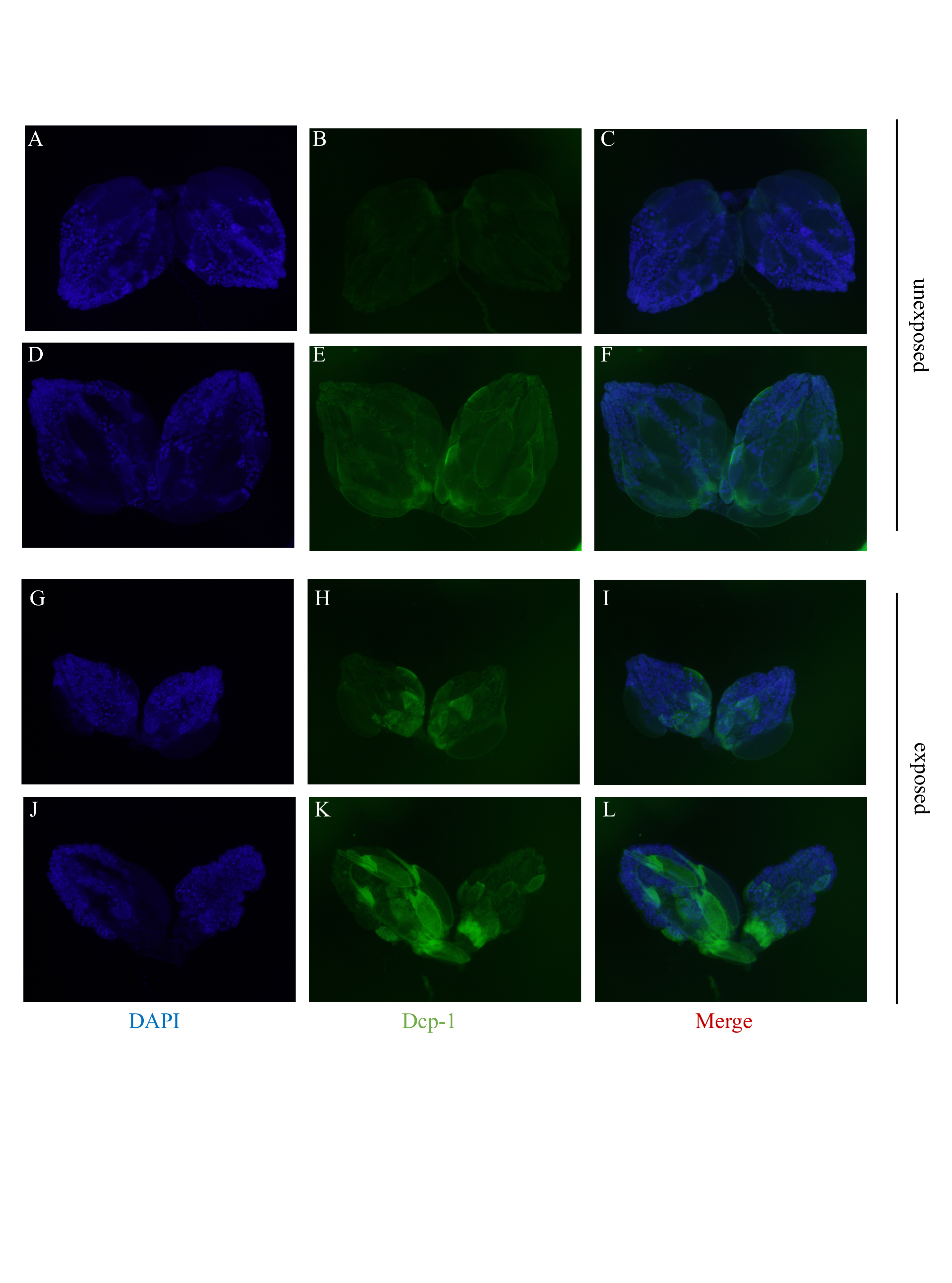

Supplement: S16 Fig — Representative images of unexposed (A-F) and wasp-exposed (G-L) ovaries stained for activated Dcp-1 are shown. DAPI (A,D,G,J), Dcp-1 (B,E,H,K), and the merged images (C,F,I,L) are shown. (TIFF) [file pgen.1007430.s016.tiff]

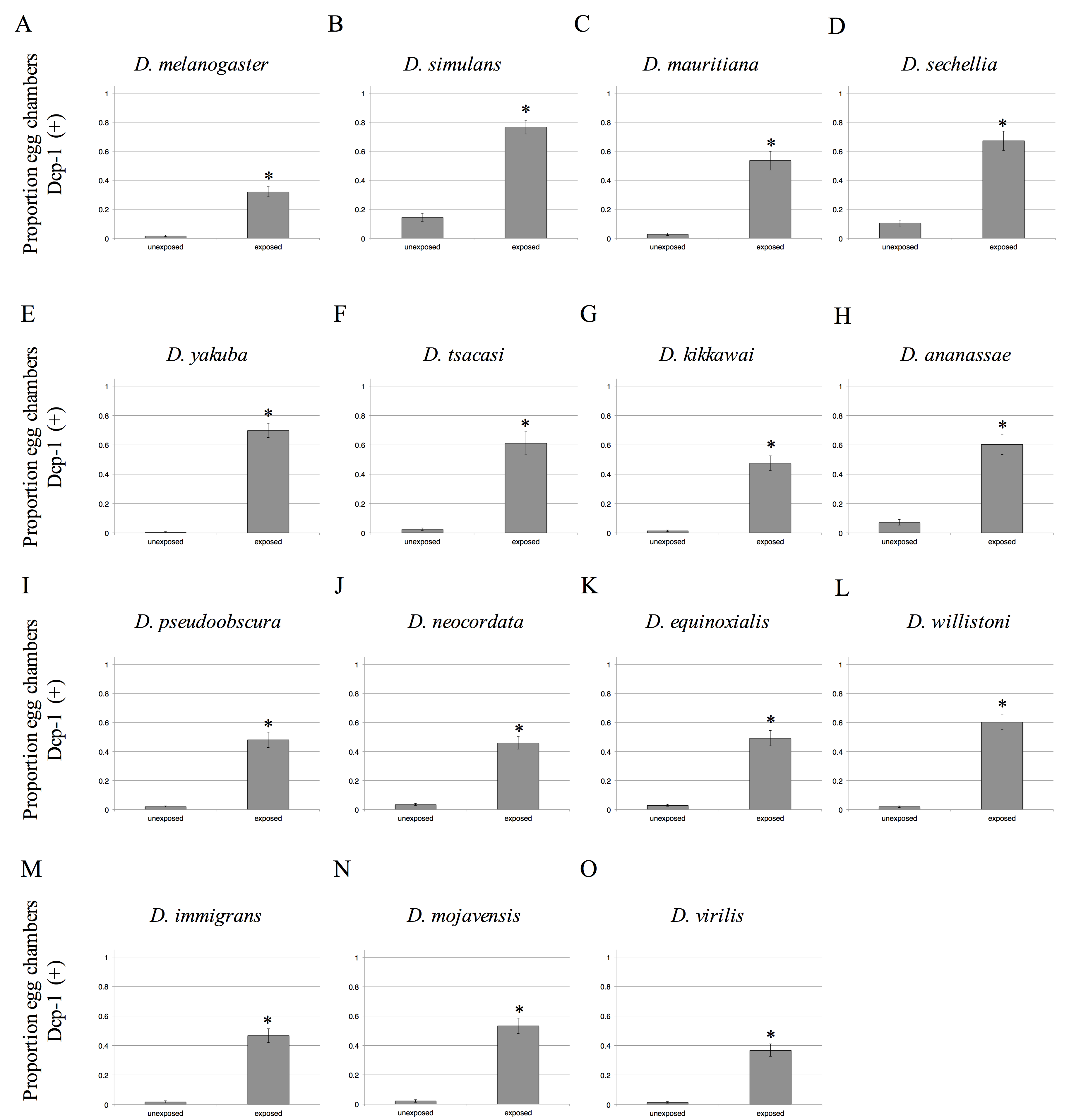

Supplement: S17 Fig — Proportion of egg chambers with Dcp-1 signal shown for (A) D. melanogaster, (B) D. simulans, (C) D. mauritiana, (D) D. sechellia, (E) D. yakuba, (F) D. tsacasi, (G) D. kikkawai, (H) D. ananassae, (I) D. pseudoobscura, (J) D. neocordata, (K) D. equinoxialis, (L) D. willistoni, (M) D. immigrans, (N) D. mojavensis, and (O) D. virilis. Error bars represent standard error (n = 36 ovaries) (*p < 0.05). (TIFF) [file pgen.1007430.s017.tiff]

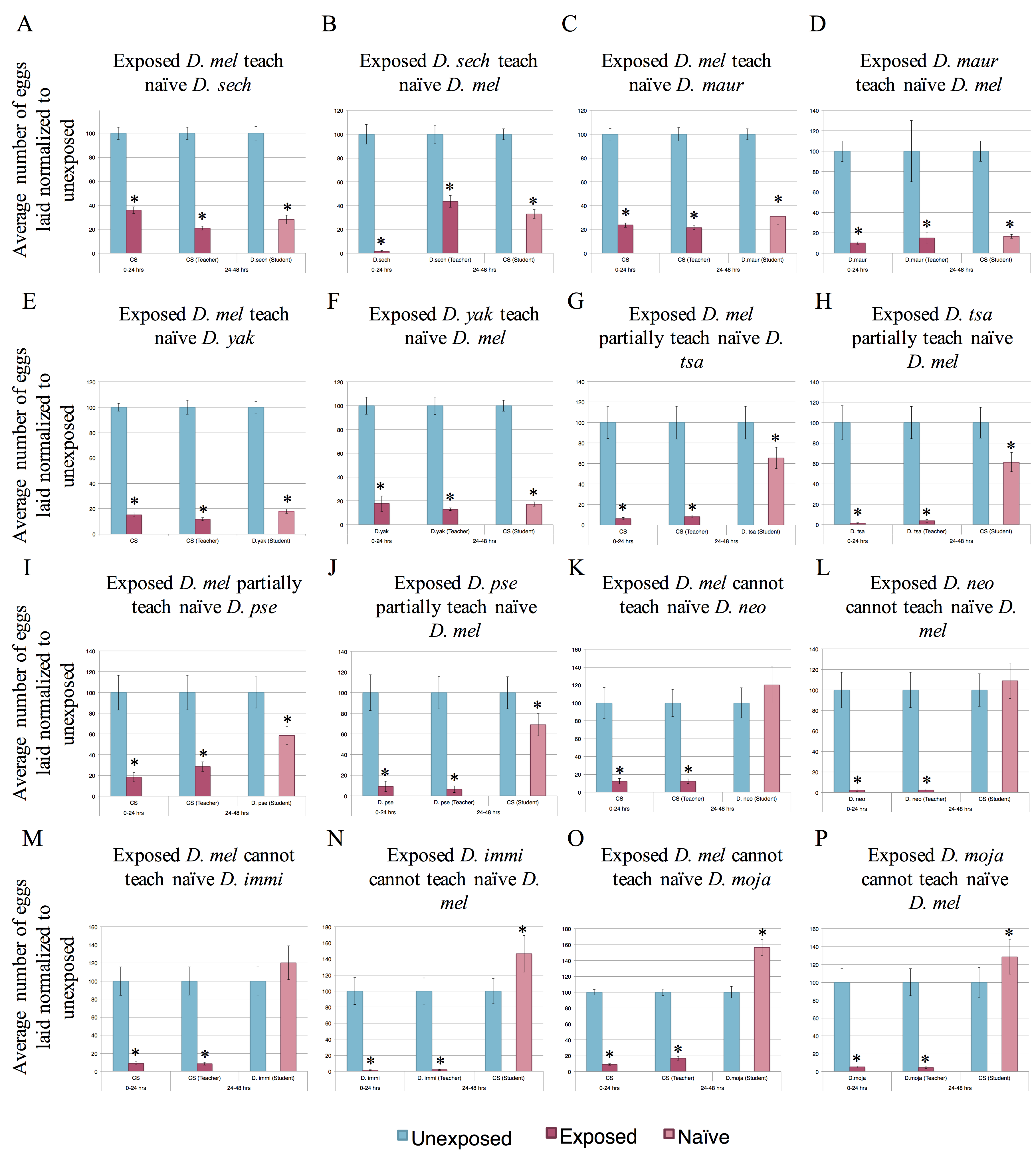

Supplement: S19 Fig — Percentage of eggs laid by exposed flies normalized to eggs laid by unexposed flies is shown. Communication between D. melanogaster and: D. sechellia (A, B), D. mauritianna (C, D), D. yakuba (E, F), D. tsacasi (G, H), D. pseudoobscura (I, J), D. neocordata (K, L), D. immigrans (M, N), and D. mojavensis (O, P), shows varying communication abilities. Error bars represent standard error (n = 12 biological replicates except for (O), n = 24 replicates) (*p < 0.05). (TIFF) [file pgen.1007430.s019.tiff]

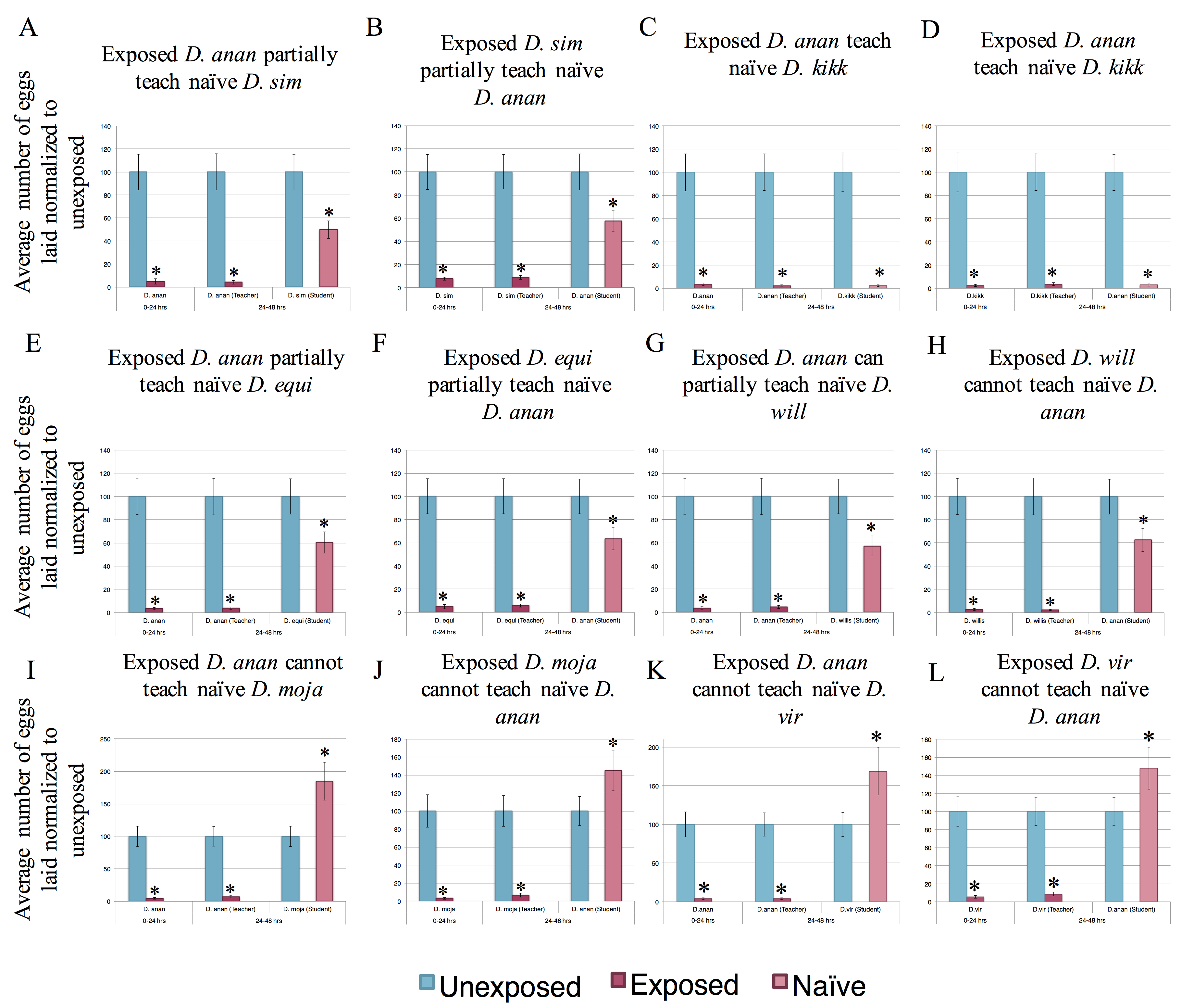

Supplement: S20 Fig — Percentage of eggs laid by exposed flies normalized to eggs laid by unexposed flies is shown. Communication between D. ananassae and: D. simulans (A, B), D. kikkawai (C, D), D. equinoxialis (E, F), D. willistoni (G, H), D. mojavensis (I, J), and D. virilis (K, L), shows varying communication abilities. Error bars represent standard error (n = 12 biological replicates) (*p < 0.05). (TIFF) [file pgen.1007430.s020.tiff]

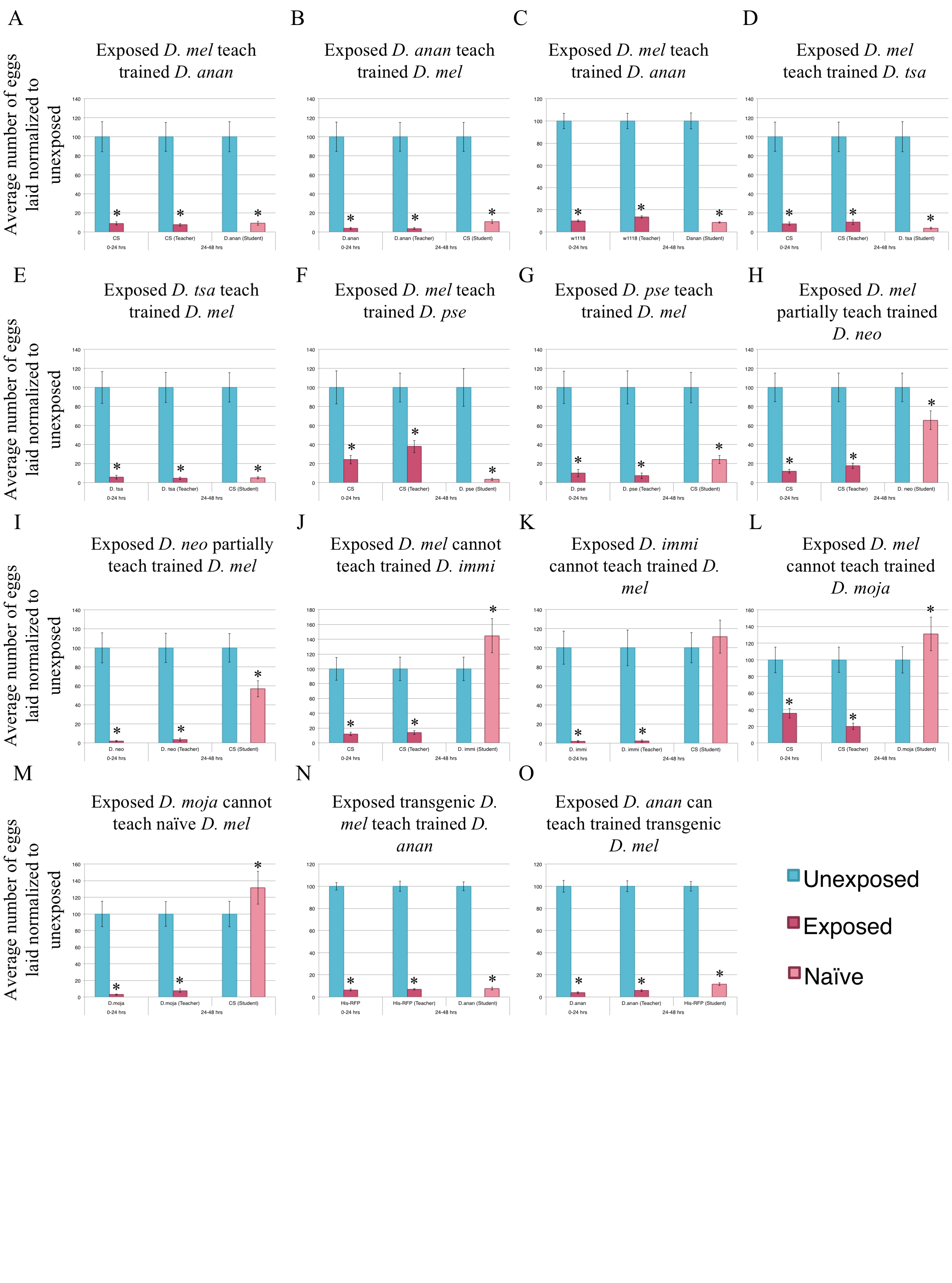

Supplement: S21 Fig — Percentage of eggs laid by exposed flies normalized to eggs laid by unexposed flies is shown. Communication between trained students D. melanogaster and: D. ananassae (second line) (A-C), D. tsacasi (D, E), D. pseudoobscura (F, G), D. neocordata (H, I), D. immigrans (J, K), and D. mojavensis (L, M). (C) An additional D. melanogaster line (w1118) learns from w1118 trained D. ananassae. Communication between D. ananassae and a transgenic D. melanogaster (Histone-RFP) occurs following training period (N, O). Error bars represent standard error (n = 12 biological replicates except for (N,O), n = 24 replicates) (*p < 0.05). (TIFF) [file pgen.1007430.s021.tiff]

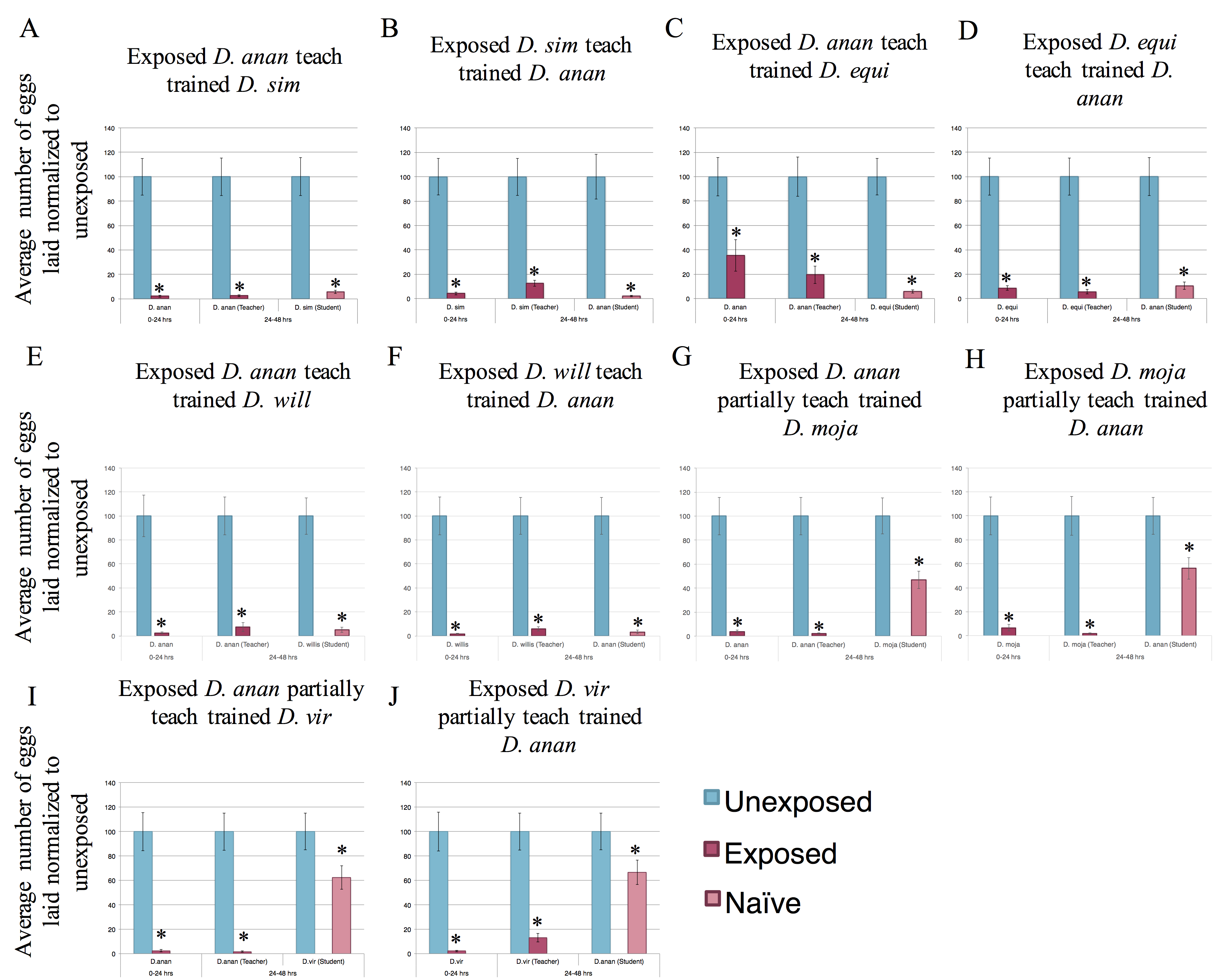

Supplement: S22 Fig — Percentage of eggs laid by exposed flies normalized to eggs laid by unexposed flies is shown. Communication between trained students D. ananassae and: D. simulans (A,B), D. equinoxialis (C,D), D. willistoni (E,F), D. mojavensis (G,H), and D. virilis (I,J). Error bars represent standard error (n = 12 biological replicates) (*p < 0.05). (TIFF) [file pgen.1007430.s022.tiff]

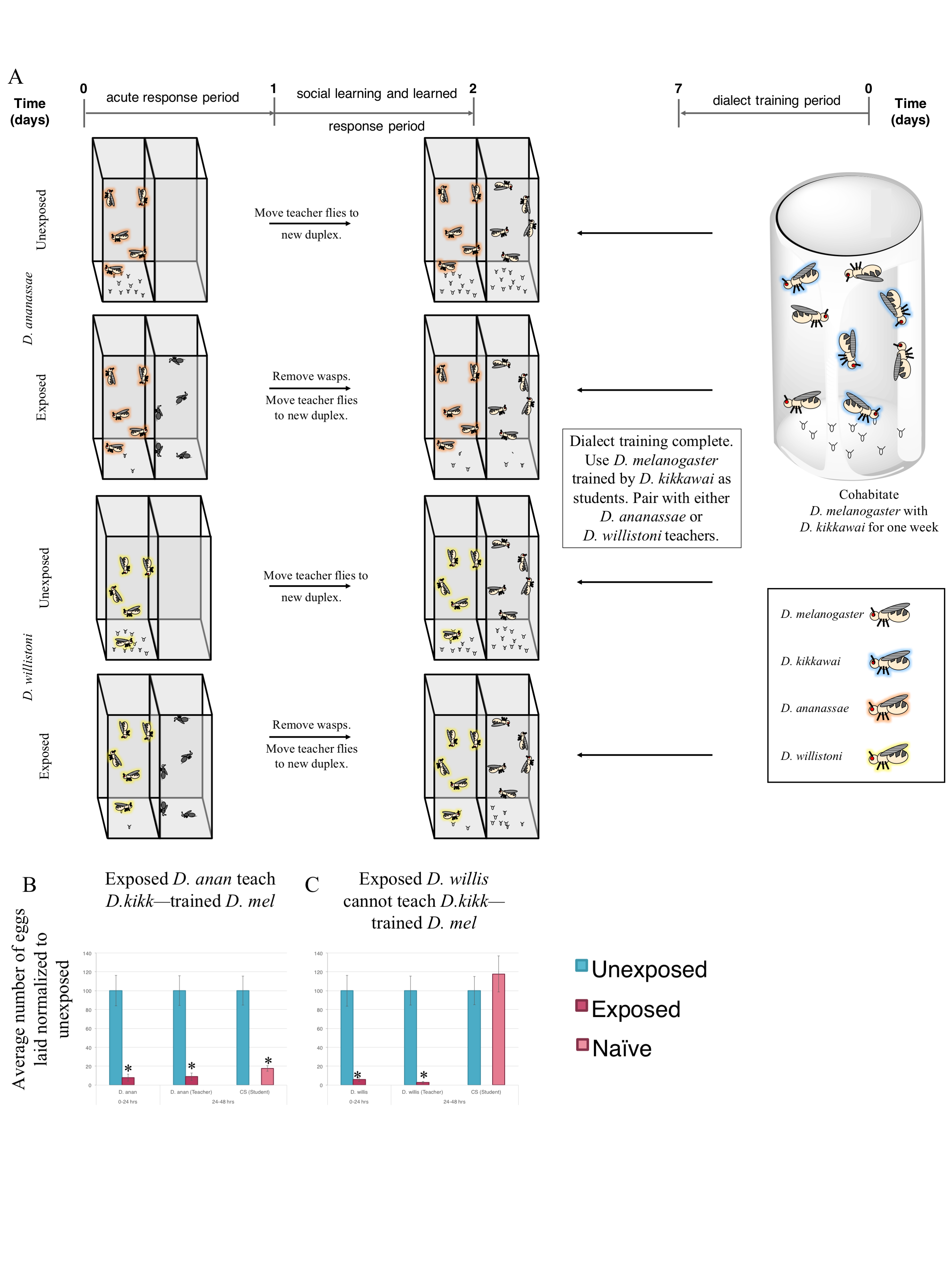

Supplement: S23 Fig — (A) Experimental design of testing specificity and generalizability of dialect. D. melanogaster were dialect trained with the sister species of D. ananassae, D. kikkawai. Following dialect training, D. melanogaster were used as students to either D. ananassae or D. willistoni teachers. Percentage of eggs laid by exposed flies normalized to eggs laid by unexposed flies is shown. Communication between D. kikkawai—trained D. melanogaster students and: D. ananassae teachers show strong communication (B), demonstrating a generalizability of the dialect signal; when paired with D. willistoni teachers, no communication is observed (C), demonstrating a signal specificity. Error bars represent standard error (n = 12 biological replicates) (*p < 0.05). (TIFF) [file pgen.1007430.s023.tiff]

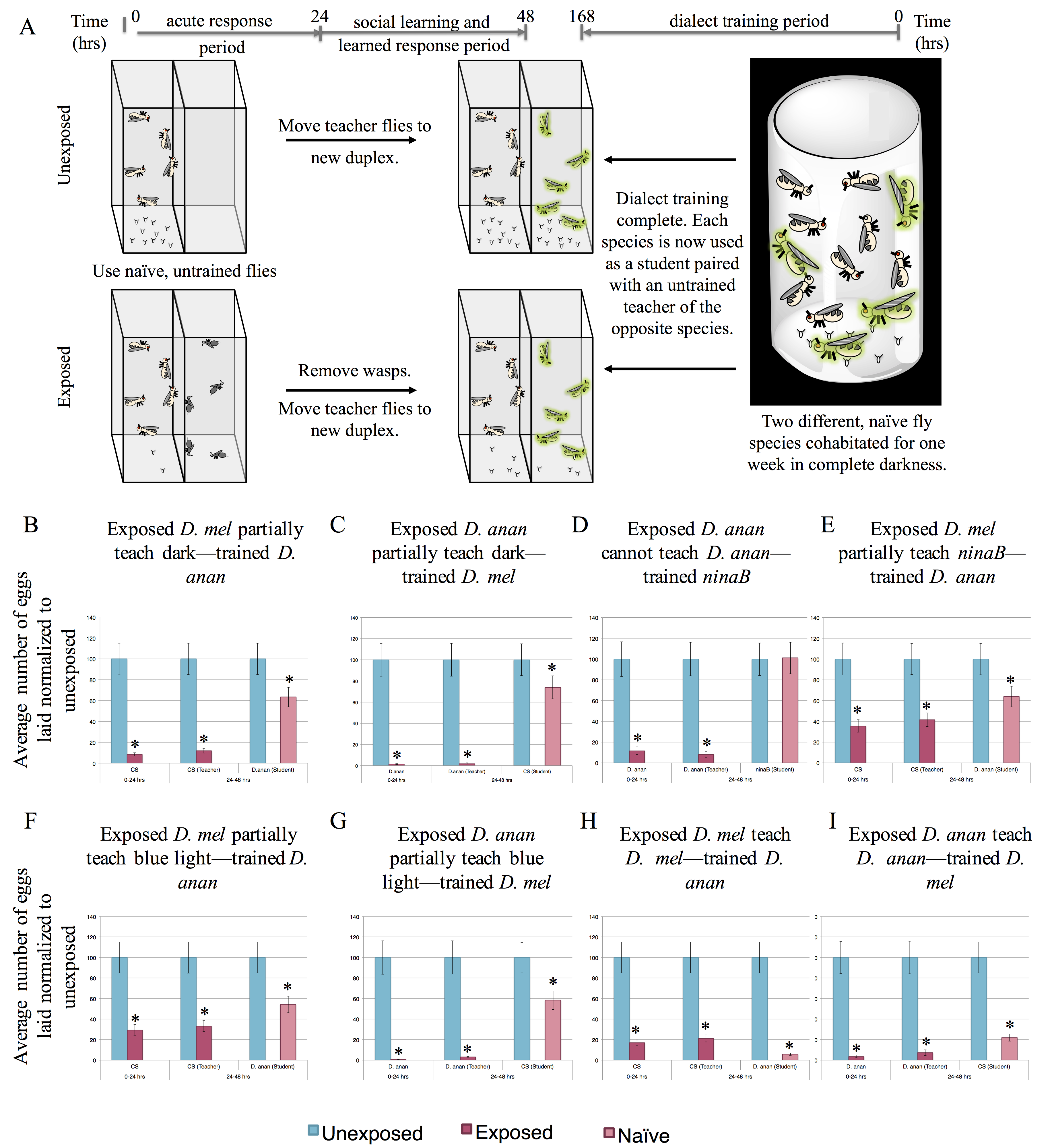

Supplement: S24 Fig — (A) Experimental design of dialect training for flies that are used as students using no visual cues by running the dialect training period in the dark (B,C). Flies do not see each other, but still interact and innervate other sensory inputs. The two species are co-incubated for one week prior to being used as students for naive, untrained teacher flies of the opposite species. Percentage of eggs laid by exposed flies normalized to eggs laid by unexposed flies is shown. Communication between trained students D. melanogaster and D. ananassae with training involving no visual cues (dark-trained), shows that visual cues necessary for dialect learning (B, C). Communication between trained students D. ananassae and the mutant ninaB (D,E). Communication between trained students D. melanogaster and D. ananassae, with training in monochromatic blue light only, shows a lack of dialect training (F, G). Communication between trained students of D. ananassae and D. melanogaster at 4.08 light intensity shows communication (H,I). Error bars represent standard error (n = 12 biological replicates) (*p < 0.05). (TIFF) [file pgen.1007430.s024.tiff]

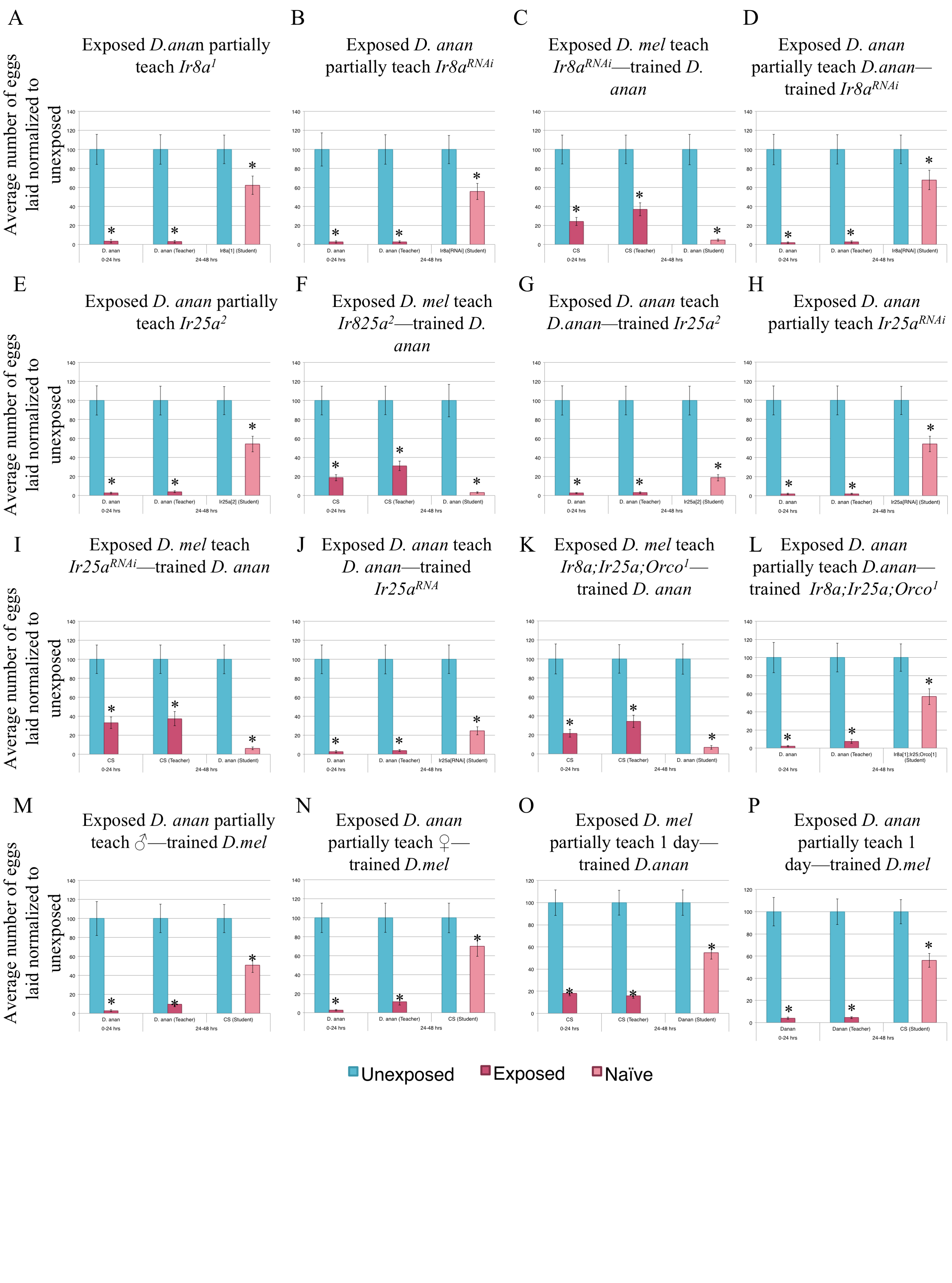

Supplement: S25 Fig — Percentage of eggs laid by exposed flies normalized to eggs laid by unexposed flies is shown. Communication between naïve D. ananassae and Ir8a1 mutant flies shows partial communication (A). Communication between naive students of Ir8a knockdown in Ir8a-expressing neurons and D. ananassae shows partial communication (B). Communication between trained students Ir8aRNAi knockdown in Ir8a expressing neurons and D. ananassae shows that IR8a receptor-mediated cues are necessary (C, D). Communication between naïve Ir25a2 mutants and D. ananassae shows partial communication (E). Communication between trained students Ir25a2 mutants and D. ananassae shows communication suggesting that IR25a receptors are not required for dialect training (F,G). Communication between naïve Ir25a knockdown in Ir25a-expressing neurons and D. ananassae shows partial communication (H). Communication between trained students Ir25aRNAi knockdown in Ir25a-expressing neurons and D. ananassae shows communication suggesting that IR25a receptors are not required for dialect training (I, J). Communication between trained Ir8a1;Ir25a2;Orco1 students and D. ananassae shows that olfactory and IR-receptor mediated cues are necessary (K, L). Communication between students D. melanogaster and D. ananassae, with training by males only or by females only, shows partial communication, suggesting that both male and female flies are required for dialect learning (M, N). Communication between trained students D. melanogaster and D. ananassae, with training for only one day, shows that 24 hours is not sufficient for dialect training (O, P). Error bars represent standard error (n = 12 biological replicates) (*p < 0.05). (TIFF) [file pgen.1007430.s025.tiff]

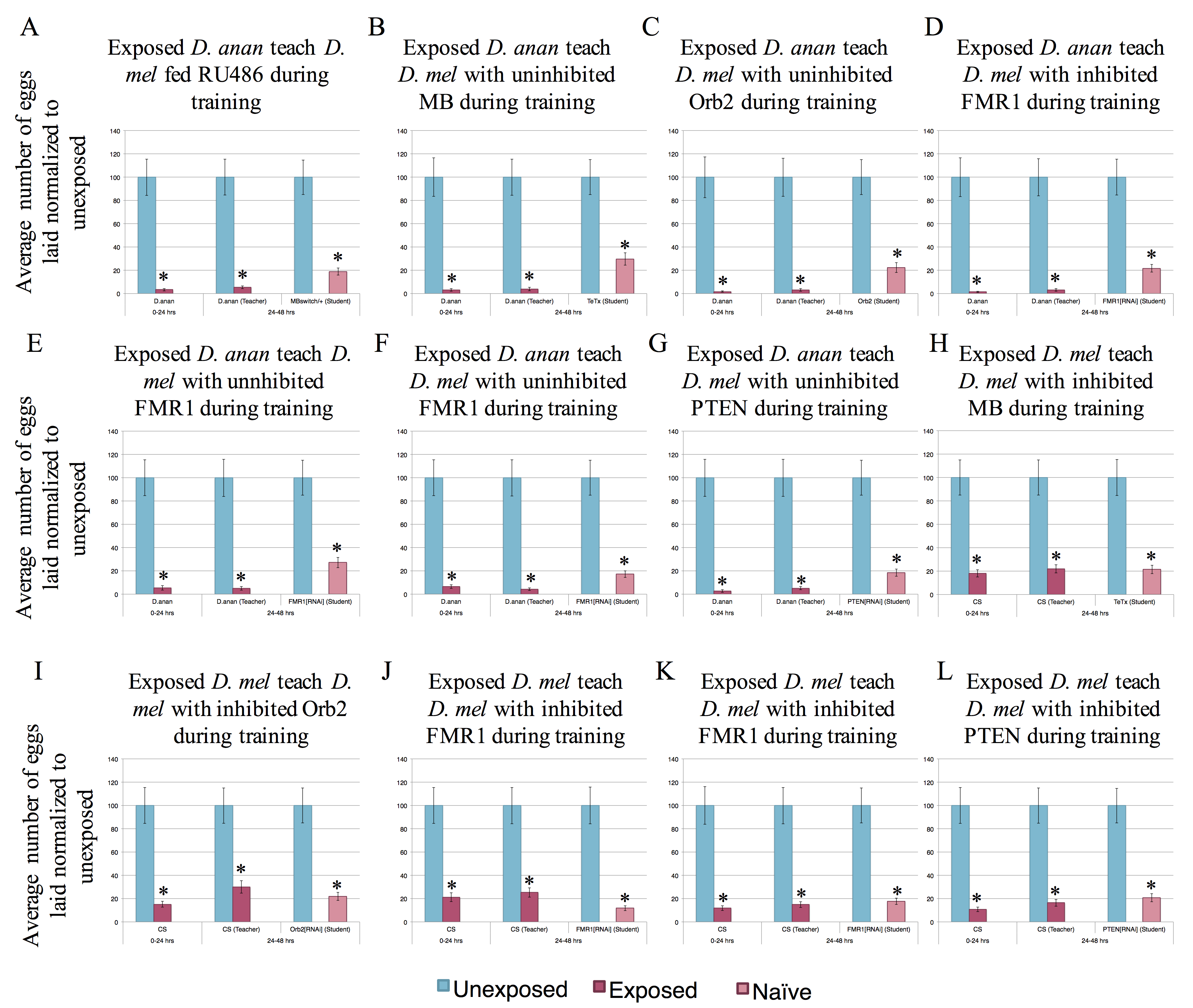

Supplement: S26 Fig — Percentage of eggs laid by exposed flies normalized to eggs laid by unexposed flies is shown. Communication between trained D. melanogaster, MBswitch/+ (outcrossed to Canton S) students and D. ananassae teachers fed RU486 during the training period shows communication between the two species, demonstrating that RU486 feeding does not perturb dialect learning (A). Communication between trained students D. melanogaster and D. ananassae, with training by flies not expressing tetanus toxin (UAS-TeTx) in the mushroom body (MB) (i.e. methanol fed), shows communication between the species (B). Communication between D. ananassae and students trained with D. ananassae with no RNAi-mediated Orb2 knockdown in the MB (i.e. methanol fed) shows communication between the species (C). Communication between D. ananassae and students trained with D. ananassae with RNAi-mediated FMR1 knockdown (strain #34944) in the MB (i.e. RU486 fed) shows that FMR1 is not required in the MB during the training period (D). Communication between D. ananassae and students trained with D. ananassae with no FMR1 knockdown (strain #24944) in the MB (i.e. methanol fed) shows wild-type behavior (E). Communication between D. ananassae and students trained with D. ananassae with no FMR1 knockdown (strain #34944) in the MB (i.e. methanol fed) shows wild-type behavior (F). Communication between D. ananassae and students trained with D. ananassae with no PTEN knockdown in the MB (i.e. methanol fed) shows wild-type behavior (G). Error bars represent standard error (n = 12 biological replicates) (*p < 0.05). Communication between various D. melanogaster lines trained by D. ananassae show wild-type communication with D. melanogaster (Canton S). Lines shown are MB switch expressing TeTx (H), Orb2RNAi (I), FMR1RNAi (strain number 27484) (J), FMR1RNAi (strain number 34944) (K), PTENRNAi (L), and were fed RU486 during cohabitation with D. ananassae. (TIFF) [file pgen.1007430.s026.tiff]

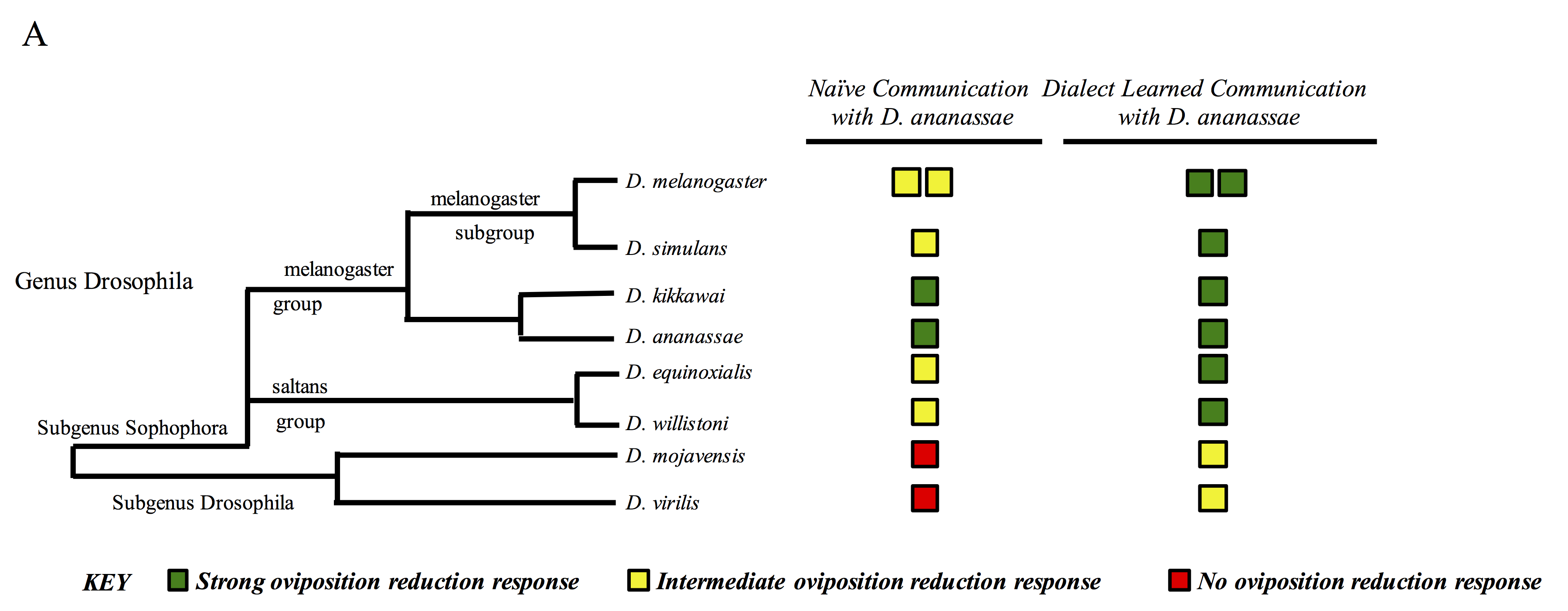

Supplement: S27 Fig — We utilize species across the genus Drosophila to show communication ability of D. ananassae (A). We observe the ability to demonstrate interspecies communication, which varies across the genus, with species closely related to D. ananassae able to communicate without barriers. More distantly related species have difficulty communicating, though the barrier can be alleviated with dialect training. Double boxes in a given row/column indicate multiple wild-type strains tested. (TIFF) [file pgen.1007430.s027.tiff]

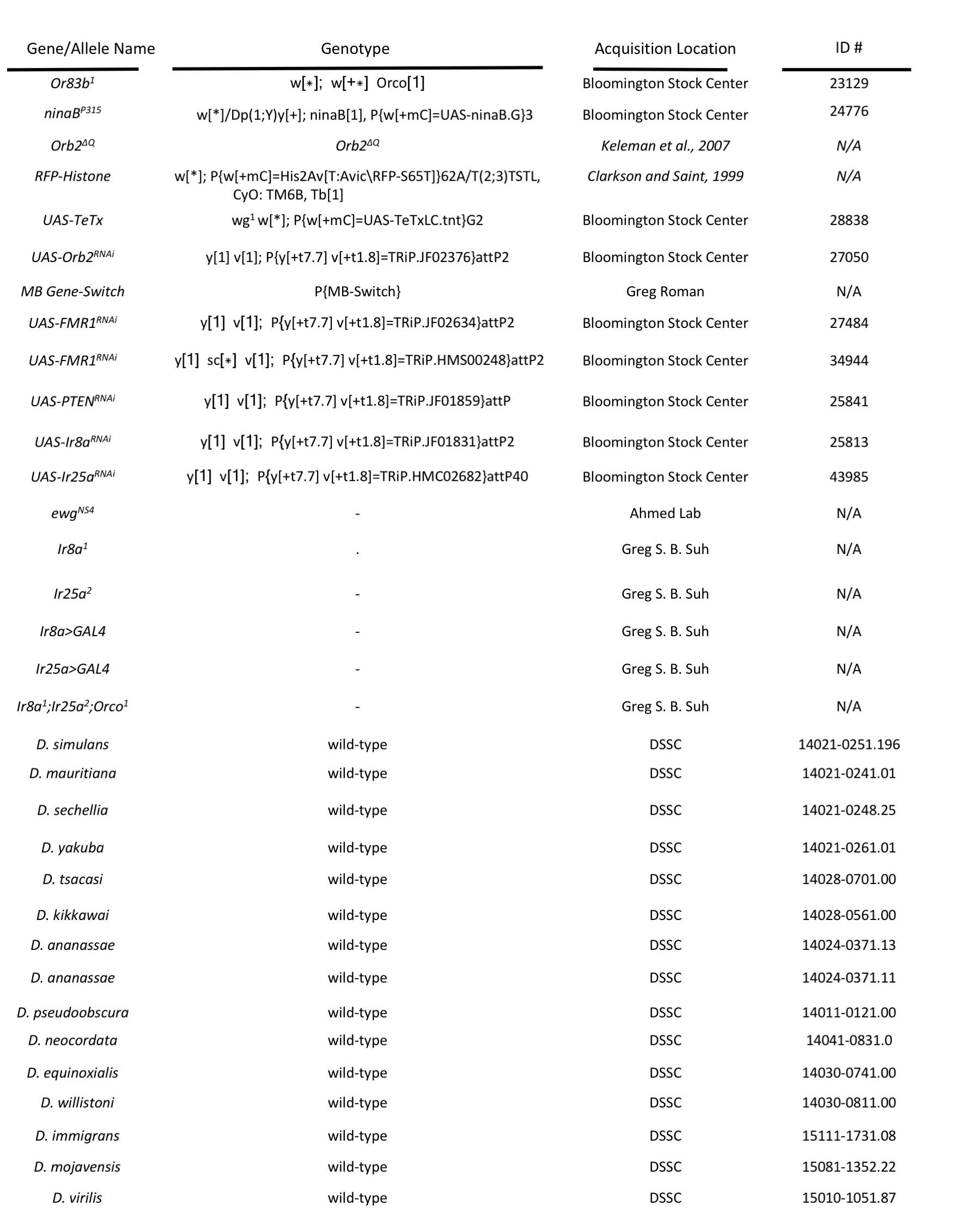

Supplement: S1 Table — (TIFF) [file pgen.1007430.s028.tiff]
